# Comparative effectiveness of lipid-nutrient spread supplementation regimens for moderate acute malnutrition in Ethiopia: The MODAM-MAM individually randomized controlled trial

**DOI:** 10.1371/journal.pmed.1005199

**Published:** 2026-08-11

**Authors:** Bailey A. Clark, Liya A. Legese, Yosef Beyene, Mesfin W. Shellemew, Hiwot Darsene, Stanley Chitekwe, Masresha Tessema, Indi Trehan, Heather C. Stobaugh

**Affiliations:** 1 Action Against Hunger USA, New York, New York, United States of America; 2 Action Against Hunger USA, Addis Ababa, Ethiopia; 3 Ethiopian Public Health Institute, Addis Ababa, Ethiopia; 4 Department of Nutritional Sciences, Oklahoma State University, Stillwater, Oklahoma, United States of America; 5 United Nations Children’s Fund, Addis Ababa, Ethiopia; 6 Ministry of Health, Addis Ababa, Ethiopia; 7 Departments of Pediatrics, Global Health, and Epidemiology, University of Washington, Seattle, Washington, United States of America; Makerere University Medical School, UGANDA

## Abstract

**Background:**

Moderate acute malnutrition (MAM), affecting over 30 million children globally, substantially increases the risk of morbidity and mortality. In 2023, the World Health Organization recommended lipid-based nutrient supplements (LNS) for children requiring supplementation for MAM, either ready-to-use supplementary food (RUSF) or ready-to-use therapeutic food (RUTF). However, evidence on the optimal product (RUTF or RUSF) and dosing strategy for children with MAM remains limited. The Modified Dosages for Acute Malnutrition MAM (MODAM-MAM) trial evaluated whether one- or two-sachet daily RUTF dosing were non-inferior to the *de facto* standard of one RUSF sachet daily for MAM supplementation.

**Methods and findings:**

We conducted a three-arm, parallel, individually randomized controlled trial in Ethiopia. Children aged 6–59 months with MAM (mid-upper arm circumference (MUAC) 11.5–12.4 cm or WHZ between –3 and –2, without edema) were randomized to: one 540-kcal sachet of RUSF/day (“1-RUSF”), one 500-kcal sachet of RUTF/day (“1-RUTF”), or two sachets of RUTF/day (“2-RUTF”). Both intervention arms (1-RUTF and 2-RUTF) were evaluated for non-inferiority to the standard (1-RUSF) using a prespecified 6-percentage-point margin. The primary outcome was anthropometric recovery, defined as both MUAC ≥ 12.5 cm and WHZ ≥ −2 for two consecutive visits within 16 weeks. In total, 2,414 children were analyzed in the intention-to-treat analysis. Recovery proportions were 84.8% (679/801) for 1-RUSF, 85.0% (674/793) for 1-RUTF, and 88.1% (721/818) for 2-RUTF. Risk differences for recovery relative to the standard of care (1-RUSF) were +0.2 percentage points (95% CI [–3.3, 3.7]; *p* = 0.944) for 1-RUTF and +3.4 percentage points (95% CI [0.0, 6.7]; *p* = 0.050) for 2-RUTF, with both intervention arms meeting the non-inferiority criteria. Anthropometric gains and median length of stay (56 days) were similar across arms. Ration use was comparable for 1-RUSF and 1-RUTF, while 2-RUTF required 1.6-fold more product. Study limitations include evaluation of a single RUSF formulation and earlier-than-typical case-finding due to intensive screening.

**Conclusions:**

Both experimental arms, one RUTF sachet daily and two RUTF sachets daily, are non-inferior to the standard dosing of one RUSF sachet daily for MAM supplementation. Although two sachets of RUTF daily yielded slightly higher recovery, the marginal benefits do not justify substantially greater resource demands. Because one RUTF and one RUSF sachet daily yielded similar treatment outcomes, either product can be considered an effective option for supplementation. Product choice may therefore be guided by supply, cost, and operational considerations.

**Trial registration:**

clinicaltrials.gov NCT06056089.

## Author summary

**Why was the study done?:**

- Moderate acute malnutrition (MAM) affects millions of children globally and is commonly treated with specialized nutritious food products, including ready-to-use supplementary food (RUSF).
- A similar product—ready-to-use therapeutic food (RUTF)—is widely used to treat severe acute malnutrition (SAM). If the same product could be used for both conditions, programs could simplify supply chains and harmonize treatment protocols.
- Recent World Health Organization guidance allows for the use of either RUSF or RUTF to treat MAM, but there is limited direct evidence comparing these approaches.
- The evidence around which children with MAM to prioritize for supplementation remains limited.

**What did the researchers do and find?:**

- We conducted a randomized controlled trial in Ethiopia comparing three treatment approaches for children with MAM: the standard one sachet per day of RUSF, one sachet per day of RUTF, and two sachets per day of RUTF.
- We found that children receiving one sachet of RUTF per day had similar recovery outcomes to those receiving standard RUSF, whereas providing two sachets of RUTF per day did not improve outcomes enough to justify the substantially greater quantity of food required.
- We also found that children with the lowest arm circumference (MUAC 11.5–12.0 cm) were at greatest risk of poor treatment outcomes. Younger age and low weight-for-age were not associated with higher risk in this study.

**What do these findings mean?:**

- These results suggest that programs can use either RUSF or RUTF at a standard daily dose to treat MAM without compromising effectiveness, while simplifying logistics and improving continuity of care between severe and moderate acute malnutrition.
- Increasing the dose of supplementary food is unlikely to provide meaningful additional benefits and is not a cost-effective strategy.
- Low MUAC alone may be sufficient to identify most children at highest risk of poor treatment outcomes, potentially simplifying decisions about who to prioritize for supplementary feeding when resources are limited.
- Study limitations included intensive community screening to improve recruitment, testing only one formulation of supplementary food, and lack of biological data to better explain the findings.

## Abstract 3

In a randomized controlled trial, Bailey Clark and colleagues investigate whether one- or two-sachet daily ready-to-use therapeutic food dosing were non-inferior to the de facto standard of one ready-to-use supplementary food sachet daily for moderate acute malnutrition supplementation in Ethiopia.

## Introduction

Acute malnutrition (AM) remains a leading cause of child morbidity and mortality worldwide, with one model suggesting it contributes to an estimated 11.5% of all deaths among children under five [1]. At any given time, approximately 43 million children under five globally are affected by AM, with over 70% presenting with moderate acute malnutrition (MAM) [2]. Although children with severe acute malnutrition (SAM) face the highest risk of near-term death, those with MAM have a 3-fold higher risk of mortality compared to well-nourished peers, along with increased vulnerability to infectious diseases and long-term deficits in physical and cognitive development [3]. Mortality risk is accentuated when MAM is combined with other risk factors such as stunting, low-birthweight, preterm birth, and not being breastfed [4].

Also referred to as moderate wasting [5], MAM is typically defined as a weight-for-height z-score (WHZ) between 2 and 3 standard deviations below the median of the 2006 World Health Organization (WHO) Child Growth Standards [6] or a mid-upper arm circumference (MUAC) of 11.5 to 12.4 cm, in the absence of bilateral pitting edema [7]. A variety of approaches have been used to manage MAM, with one of the most common involving the provision of specially formulated foods (SFFs) to supplement children’s home diets [8]. The two major types of SFFs for MAM supplementation are fortified blended flours (FBFs) and lipid-nutrient spreads (LNSs) [9].

The most commonly used LNS is ready-to-use supplementary food (RUSF), derived from the previously developed ready-to-use therapeutic food (RUTF) that has established itself as the standard of care for nutritional treatment of uncomplicated SAM [10,11]. RUSF has lower milk content, higher micronutrient content, and slightly higher calories (540 kcal versus 500 kcal) in a typical sachet (100 g versus 92 g) than RUTF. While RUTF was originally designed to serve as a complete nutritional treatment for children with SAM, RUSF was designed as a supplement to a home diet for children with MAM [12]. Historically, RUSF has had a significantly lower unit cost than RUTF, although this difference has decreased over time [13,14]. The use of different products (RUSF and RUTF) for two similar clinical conditions (MAM and SAM) has raised the practical question of whether there are operational and logistical advantages to using a single food (RUTF) for both severities of AM.

In recent years, experimental treatment protocols have explored this possibility. Trials such as ComPAS [15] and OptiMA [16] have tested unified MAM and SAM treatment approaches using RUTF for both AM diagnoses and have incorporated simplified or reducing dosage schemes. These models aim to reduce complexity for frontline health workers, streamline logistics and supply chains, and improve program efficiency. By standardizing treatment across the continuum of care for AM, novel protocols may also enhance coverage, continuity of care, and cost-effectiveness in resource-constrained settings. Although promising, these approaches rely on assumptions regarding dose adequacy for MAM and provide limited direct evidence comparing RUSF and RUTF at different dosages.

In 2023, WHO released its first formal guidance on the management of MAM [7], including the recommendation to prioritize LNS over FBF [17]. Both RUSF and RUTF were deemed equivalent options, though this recommendation was based on relatively limited evidence [18]. The WHO recommendation for the optimal dosing of supplementary food was also based on theoretical calculations, in the context of the *de facto* practice of providing children with one sachet of RUSF per day [18]. Still, it remains unknown whether a higher dose of LNS supplementation would lead to improved outcomes. Robust clinical trial evidence is thus needed to validate or refine WHO guidance and to inform operational practice.

The Modified Dosages for Acute Malnutrition Treatment (MODAM) study is a series of three concurrently conducted randomized controlled clinical trials in Ethiopia designed to provide practical guidance on a number of these questions [19]. Ethiopia suffers from a heavy burden of acute malnutrition, with a nationwide prevalence of 11% in 2023 [20] and an estimated 57% of all child deaths due to malnutrition [21]. The years of life lost due to acute malnutrition is estimated to be highest in Somali Region (where Gode is located) and third highest in Oromia (where Teltele is located). Despite many operational challenges [22], community-based management programs for acute malnutrition in Ethiopia have had commendable success, helping avert an average of 34,000 deaths per year at an average cost of $762 each [23]. Optimizing treatment programs by improving coverage and cost-efficiency remains an ongoing public health priority as the Ethiopian healthcare system confronts funding and structural challenges [24,25].

In the MODAM-MAM trial, we address key evidence gaps highlighted in the 2023 WHO wasting guidelines by directly comparing the use of RUSF, the standard LNS product for MAM supplementation, with two operationally relevant RUTF regimens. The primary objective was to determine whether one sachet of RUTF per day (1-RUTF) and two sachets per day (2-RUTF) are non-inferior to the standard one sachet of RUSF regimen (1-RUSF) in achieving anthropometric recovery. These dosing strategies were selected to represent a relatively lower and a relatively higher dosage of RUTF, reflecting operationally feasible options for programs considering integrated management of acute malnutrition. By jointly evaluating both the product type and dosage of supplementary food within the same randomized trial, this study fills major evidence gaps identified by WHO and national programs and aims to inform more effective, efficient, and scalable approaches to MAM management in high-burden settings.

## Methods

### Ethics statement

Informed consent was obtained from parents or legal guardians through verbal and written consent. For non-literate caregivers, written consent was documented using fingerprints. Ethical approval was obtained from the Ethiopia Public Health Institute (EPHI-IRB-4982023) and additional approval was received from the Ethiopia Federal Ministry of Health.

### Study design and participants

The MODAM-MAM trial was an individually randomized, three-arm, parallel-group controlled trial with equal allocation (1:1:1). The primary objective was to evaluate whether two RUTF regimens—one RUTF sachet per day and two RUTF sachets per day—were non-inferior to the current standard of care (*i.e.,* one sachet of RUSF per day) for MAM management. Children were randomized to one of three regimens: one sachet (100 g; 540 kcal) of RUSF per child per day (“1-RUSF”), one sachet (92 g; 500 kcal) of RUTF per child per day (“1-RUTF”), or two sachets (1,000 kcal) of RUTF per child per day (“2-RUTF”). Each RUTF arm was evaluated for non-inferiority in achieving anthropometric recovery relative to the standard 1-RUSF using a prespecified 6-percentage-point margin. All three arms were implemented concurrently in a parallel-group design.

The trial was registered on clinicaltrials.gov as NCT06056089 (registered September 20, 2023) and a detailed protocol has been published previously [19]. There were no significant deviations from the study protocol. This study is reported as per CONSORT 2025 guideline (S1 Checklist). Additional information regarding the ethical, cultural, and scientific considerations specific to inclusivity in global research is included in the Supporting Information (S2 Checklist).

The trial was conducted in two geographically and contextually distinct *woredas* (districts) in Ethiopia: Teltele Woreda in Oromia Region and Gode Woreda in Somali Region. Study locations were selected due to high food insecurity, burden of acute malnutrition, and other contextual vulnerabilities. Study activities were implemented by trained nutrition researchers and embedded within the existing public health infrastructure managed by the Ethiopia Federal Ministry of Health across 17 rural health posts in Teltele and 10 health posts, three health centers, and two *kebele*-level (village-level) locations in Gode.

During the study period, services at participating sites were delivered by trained roving study teams working alongside Health Extension Workers (HEWs). This approach was used to ensure adherence to the study protocol and execute the additional responsibilities involved in study implementation. In Ethiopia, targeted supplementary feeding program (TSFP) services are typically provided on a designated day each week at health posts. Study teams staffed these same scheduled clinic days as they rotated across facilities. Health Extension Workers continued to support community screening, referral, and clinic activities throughout the trial. All participants received standard TSFP services in accordance with national guidelines, and routine care was not influenced by study arm and was provided equally across groups.

Children were eligible if they were aged 6–59 months, resided in the study catchment areas, and met the diagnostic criteria for MAM. Exclusion criteria included prior treatment for acute malnutrition within the past three months, signs of severe illness requiring inpatient care, or chronic conditions or disabilities that could interfere with feeding or treatment adherence. Participants were recruited through a combination of routine facility-based screening and targeted community mobilization.

Eligibility was assessed using standard field anthropometric and clinical measures including weight, length, MUAC, and presence of bilateral pitting edema. WHZ was determined using gender-specific look-up tables based on the 2006 WHO Growth Standards [6] and later verified in Stata SE 18 (StataCorp LLC, College Station, TX, USA). Children were classified as MAM if they had a WHZ between ≥ –3 and <–2, and/or a MUAC ≥ 11.5 cm and < 12.5 cm, with no edema.

### Randomization and masking

Participants were randomly assigned to one of three study arms using block randomization, with a fixed block size of 18. For each block, researchers prepared 18 paper allocations (six per study arm) and inserted into small opaque envelopes to ensure allocation concealment. At enrollment, caregivers selected one envelope containing an intervention code corresponding to the assigned study arm from a larger opaque envelope, which the study staff then opened and recorded. Blocks were not linked to specific facilities or study locations. Instead, each *woreda*’s roving research team carried a set of envelopes and used them sequentially as they moved across facilities. After all 18 allocations within a block had been used, the team proceeded to the next block. Randomization was fully unstratified and occurred at the individual child level.

The three intervention arms were: (1) Control (1-RUSF group): one 100-g sachet of RUSF providing 540 kcal per day; (2) 1-RUTF group: one 92-g sachet of RUTF providing 500 kcal per day; (3) 2-RUTF group: two 92-g sachets of RUTF providing a total of 1,000 kcal per day.

Due to the nature of the intervention, masking of participants and caregivers was not feasible. However, data collection and the implementation of the intervention (*i.e.*, dosing of the various trial foods) were conducted in a separate process to keep the field-level researchers masked to study assignments during anthropometry, data collection, and outcome determination. Outcomes were based solely on standardized anthropometric criteria, and data analysts remained masked to group allocation through coded datasets until after primary analyses were complete.

### Procedures

Upon enrollment, a brief questionnaire was administered to caregivers to collect child and household-level information, including demographics, food security, asset ownership, maternal education, recent medical history, and breastfeeding status.

Children were reassessed at the health facility every 14 days for up to 16 weeks following enrollment, in accordance with national guidelines. At each follow-up visit, anthropometry was repeated, recent illness symptoms and appetite were queried, and an assessment as made of how much intervention food the child consumed at home in the prior two weeks based on verbal recall and a count of returned empty sachets.

Safety was monitored throughout the study through routine clinical assessments conducted at each follow-up visit, documentation of clinical deterioration, hospitalization, progression to SAM, and death, active tracing of children who missed scheduled visits where feasible, and regular review by an independent Data and Safety Monitoring Board (DSMB).

Weight was measured using Seca 354 digital infant scales (precision 5 g) or ADE M321600 standing scales (precision 100 g), length using PKP MB31 rigid pediatric length boards (precision 0.1 cm), and MUAC using standard non-stretch color-coded MUAC tapes (precision 0.1 cm). All measurement supplies were procured through the United Nations Children’s Fund (UNICEF). Field staff had prior CMAM experience and completed a comprehensive training on study procedures, followed by periodic on-site supervision and refresher training.

At each visit, children were classified as having MAM, SAM, or no AM based on their anthropometry. After ensuring that they were not suffering from any clinical complications warranting referral to a medical facility, they were then provided with a 14-day supply of the assigned dosage and food type according to their randomized group. Children who met recovery criteria (MUAC ≥ 12.5 cm and WHZ ≥ –2 for two consecutive visits) were discharged from care. In accordance with national practice, recovered children received one final 14-day ration at the visit when recovery was confirmed, after which no additional supplementation was provided. Children who missed a scheduled visit did not receive that fortnight’s ration, consistent with standard practice where supplementary foods are distributed only during scheduled clinic visits. All rations dispensed were recorded, enabling calculation of the total number of sachets provided per child. Nutrition counselling, including the correct use of the specially formulated foods, was provided to caregivers at every visit.

### Outcomes

The primary outcome was recovery from AM by 16 weeks. Recovery was defined as achieving a WHZ ≥ −2, MUAC ≥ 12.5 cm, and no edema for two consecutive follow-up visits, as defined by the 2023 WHO guidelines [7]. Children who met these criteria at only a single visit, including the final (16-week) visit, were classified as non-recovered for the primary outcome. Non-recovery was further disaggregated into the following secondary outcomes: deterioration to SAM at any point during treatment, remaining with MAM (*i.e.*, failure to reach recovery criteria by 16 weeks), referral to inpatient care for medical or nutritional complications, default (*i.e.*, absent for three consecutive visits), and death. Children who fulfilled recovery criteria for two consecutive visits and subsequently discharged were considered recovered for the primary outcome. Any deterioration after discharge was captured separately as a post-exit outcome but did not alter the primary recovery classification.

Additional secondary outcomes included length of stay in treatment (number of days from admission to the final visit at which the final outcome occurred—*i.e.,* recovery, default, death, referral, or completion of 16-weeks of supplementation), total number of sachets of LNS distributed per child, and changes in anthropometric indices (MUAC, height, weight, WHZ, weight-for-age z-score [WAZ], height-for-age z-score [HAZ]) during treatment.

All protocol-specified primary and secondary treatment outcomes are reported in this manuscript, with the exception of morbidity during treatment. Visit-level morbidity data were collected as repeated clinical monitoring measures rather than a single interpretable trial outcome and are therefore not reported. Medium-term follow-up outcomes will be reported separately. Planned ancillary outcomes related to body composition and immunological function were not collected because funding for those studies was not obtained.

### Statistical analysis

#### Sample size.

Sample size was calculated to assess whether the primary outcome of anthropometric recovery in each RUTF arm was non-inferior to the control 1-RUSF regimen, using a pre-specified −6-percentage-point non-inferiority margin, 80% power, a one-sided alpha of 0.05, and an expected baseline recovery proportion of 75%. An additional 10% was added to account for potential exclusions due to incorrect enrollments or data errors, resulting in a target sample size of approximately 800 children per arm (2,400 total) [19].

#### Data management.

Primary data were collected on paper-based forms and double-entered daily into Microsoft Access Databases (Microsoft Corp., Redmond, WA, USA). Data were inspected regularly for plausibility, completeness, and internal consistency. Implausible anthropometric values—such as implausible z-scores (*e.g.,* WHZ <–5 or >+5; HAZ <–6 or >+6; WAZ <–6 or >+5), extreme changes between visits, or internally inconsistent height, weight, and MUAC recordings—were flagged, verified against paper records, and corrected when appropriate. All analyses were conducted using Stata SE 18 (StataCorp LLC, College Station, TX, USA).

#### Analysis populations.

The primary analysis used an intention-to-treat (ITT) population. This population included all randomized children who met MAM eligibility criteria at admission and contributed at least one post-enrollment anthropometric assessment. Children who were subsequently determined to be non-malnourished or have severe wasting at admission, to have irreconcilable anthropometric data at enrollment or at the first follow-up visit, or to have been enrolled at one health facility that became inaccessible during follow-up were excluded from the ITT population because their primary outcome could not be meaningfully defined.

A per-protocol (PP) analysis was conducted *post hoc*, consistent with analytical approaches used in comparable acute malnutrition treatment trials [26] and as often recommended for non-inferiority studies. The PP analysis included only children who (1) received the correctly allocated study product and dosage at >80% of attended visits, (2) did not default, and (3) were not incorrectly discharged (*i.e.,* discharged as recovered without meeting recovery criteria).

#### Outcome analysis.

The primary analysis consisted of unadjusted comparisons between randomized groups. For both RUTF comparisons (1-RUTF versus 1-RUSF and 2-RUTF versus 1-RUSF), we estimated the risk difference (RD) in recovery with corresponding two-sided 95% confidence intervals (CIs). Non-inferiority was concluded if the lower bound of the CI exceeded the prespecified non-inferiority margin of −6 percentage points. Although the study was powered assuming a one-sided α of 0.05 (equivalent to a two-sided 90% confidence interval), non-inferiority was evaluated using two-sided 95% CIs (equivalent to a one-sided α of 0.025), representing a more conservative inferential criterion.

We did not apply formal multiplicity corrections (*e.g.*, Bonferroni adjustment) because the number of planned comparisons was small and such corrections would substantially reduce power for detecting clinically important differences, in alignment with CONSORT guidance for multi-arm randomized trials [27]. Instead, we emphasize the magnitude and precision of effect estimates, and treated secondary and subgroup analyses as exploratory, without formal hypothesis testing.

Binary outcomes (deterioration to SAM, remained MAM, referred to hospital, defaulted, died) were compared using Fisher’s exact test with absolute risk differences (RD) and Newcombe-Wilson 95% CIs. The non-parametric Mann–Whitney *U* test was used to compare medians and distributions of continuous variables since most were not normally distributed. Accordingly, continuous variables were summarized as medians with interquartile ranges (IQRs). Median differences with 95% confidence intervals were estimated using the Hodges-Lehmann method, a nonparametric estimate of the median difference between groups. There was no missing data for the primary outcome. For longitudinal measures, data were not recorded for visits that participants did not attend; analyses were conducted using available observations without imputation.

Time-to-recovery was analyzed using Kaplan–Meier curves, with differences assessed using the log-rank test. Separately, length of stay (LOS) was summarized using medians and interquartile ranges and compared between study arms using the Mann–Whitney *U* test, with median differences estimated using the Hodges-Lehmann method. As a secondary sensitivity analysis, covariate-adjusted differences in LOS were estimated using a Poisson regression model with robust standard errors, including the same covariates as the adjusted recovery analysis.

Growth trajectory analyses were conducted to examine patterns of anthropometric change over time while accounting for repeated measurements and varying follow-up durations. MUAC and WHZ trajectories were modeled using mixed-effects models with random intercepts for child and flexible (non-linear) functions of time, including interactions between study arm and time, and adjusted for the same anthropometric, demographic, clinical, and geographic covariates as the primary analyses. Growth velocity analyses were additionally performed by calculating MUAC gain (mm/week) and weight gain (g/kg/day) between consecutive visits and relating these to nutritional status at the start of each interval.

#### Sensitivity analyses.

As a secondary sensitivity analysis, we fit multivariate logistic and linear regression models to assess whether adjustment for prognostic baseline characteristics materially altered treatment estimates and to evaluate the potential influence of chance baseline imbalances arising despite randomization. Because treatment allocation was individually randomized, the primary analysis was based on unadjusted comparisons between study arms.

Candidate covariates were identified *a priori* based on biological plausibility, programmatic relevance, and prior research. These included child age and sex; study location (*woreda*, modeled as fixed effects); admission anthropometry; breastfeeding status; recent illness (diarrhea, fever, cough); household demographic characteristics (female-headed household, number of children under 10 years); household displacement status; household livelihoods; and food insecurity. Only variables collected in the enrollment survey and available in the analytic dataset were considered.

Collinearity among candidate covariates was formally assessed using variance inflation factors (VIFs) and pairwise correlation matrices. A conservative threshold of VIF > 5 was used to identify problematic multicollinearity. Baseline height, weight, and height-for-age z-score (HAZ) exhibited extreme multicollinearity (VIFs >70–200) and were therefore excluded from adjusted models. After removal of these highly collinear measures, baseline MUAC, weight-for-height z-score (WHZ), and weight-for-age z-score (WAZ) demonstrated acceptable collinearity (all VIFs < 2) and were retained, as each captures a distinct physiological dimension of nutritional status at enrollment.

Treatment effect estimates were highly consistent across multiple model specifications. The final model was selected based on interpretability and stability of treatment effect estimates rather than statistical significance of individual covariates. The final adjusted model included study arm, child age and sex, *woreda*, baseline MUAC, WHZ, and WAZ, breastfeeding status, recent diarrhea, and the number of children under 10 years in the household. Results are presented as odds ratios with 95% confidence intervals.

#### Risk factor and sub-group analyses.

In addition to the primary analyses, we conducted a series of *post hoc*, exploratory analyses motivated by the 2023 WHO guideline, which highlights younger age, lower MUAC, and low WAZ as potential criteria for prioritizing children for specially formulated foods when resources are limited (recommendation B13) [17,18]. These analyses were not pre-specified in the protocol as the guideline was published after study initiation. No adjustments were made for multiple comparisons and findings should be considered exploratory and hypothesis generating.

The exploratory analyses addressed two distinct questions. First, we conducted a prognostic analysis to examine whether children meeting WHO-defined “higher-risk” criteria experienced different outcomes than other enrolled children, irrespective of treatment assignment. This analysis was intended to assess whether the risk factors highlighted in recommendation B13 were associated with poorer outcomes within the trial population. To do so, we created binary indicators for MUAC < 12.0 cm, WAZ < –3, and age <24 months. Recovery, length of stay, total ration use, and growth outcomes were summarized across categories of each characteristic.

To further explore the prognostic value of these enrollment characteristics, we examined outcomes according to combinations of low age, low MUAC, and low WAZ. While WHO 2023 wasting guidelines (recommendation B13) [7] consider these factors *individually* when identifying children who may be prioritized for nutritional supplementation [17], we additionally evaluated the cumulative effect of possessing multiple risk factors. We fit a logistic regression model including the three binary indicators to estimate recovery probabilities across combinations of these risk factors and used marginal predictions to estimate the probability of recovery for each risk factor profile.

Second, we explored potential effect measure modification by age, MUAC, and WAZ to assess whether the relative effects of the three study regimens differed within these higher-risk groups. We repeated the primary unadjusted treatment comparisons within strata defined by age (6–23 versus 24–59 months), MUAC (11.5–11.9 versus 12.0–12.4 cm), and WAZ (<–3 versus ≥–3). Within each stratum, primary and secondary outcomes were compared using the same methods as in the main analysis. Interaction testing was also conducted using logistic regression models including treatment arm, subgroup indicator, and treatment-by-subgroup interaction terms to assess whether treatment effects differed across subgroups.

### Use of artificial intelligence

ChatGPT (OpenAI) was used on a limited basis to assist with select editing and refinement of manuscript drafting. Suggestions generated by ChatGPT were reviewed by the authors and incorporated only when deemed accurate and appropriate. Artificial Intelligence (AI) was not used for study conceptualization, study design, data collection, data management, statistical analyses, or generation of study results. All hypotheses, interpretations, results, conclusions, limitations, and implications presented in this manuscript represent the authors’ own work and ideas. The authors retain full responsibility for the content of the manuscript.

## Results

### Study population

**Fig 1:**
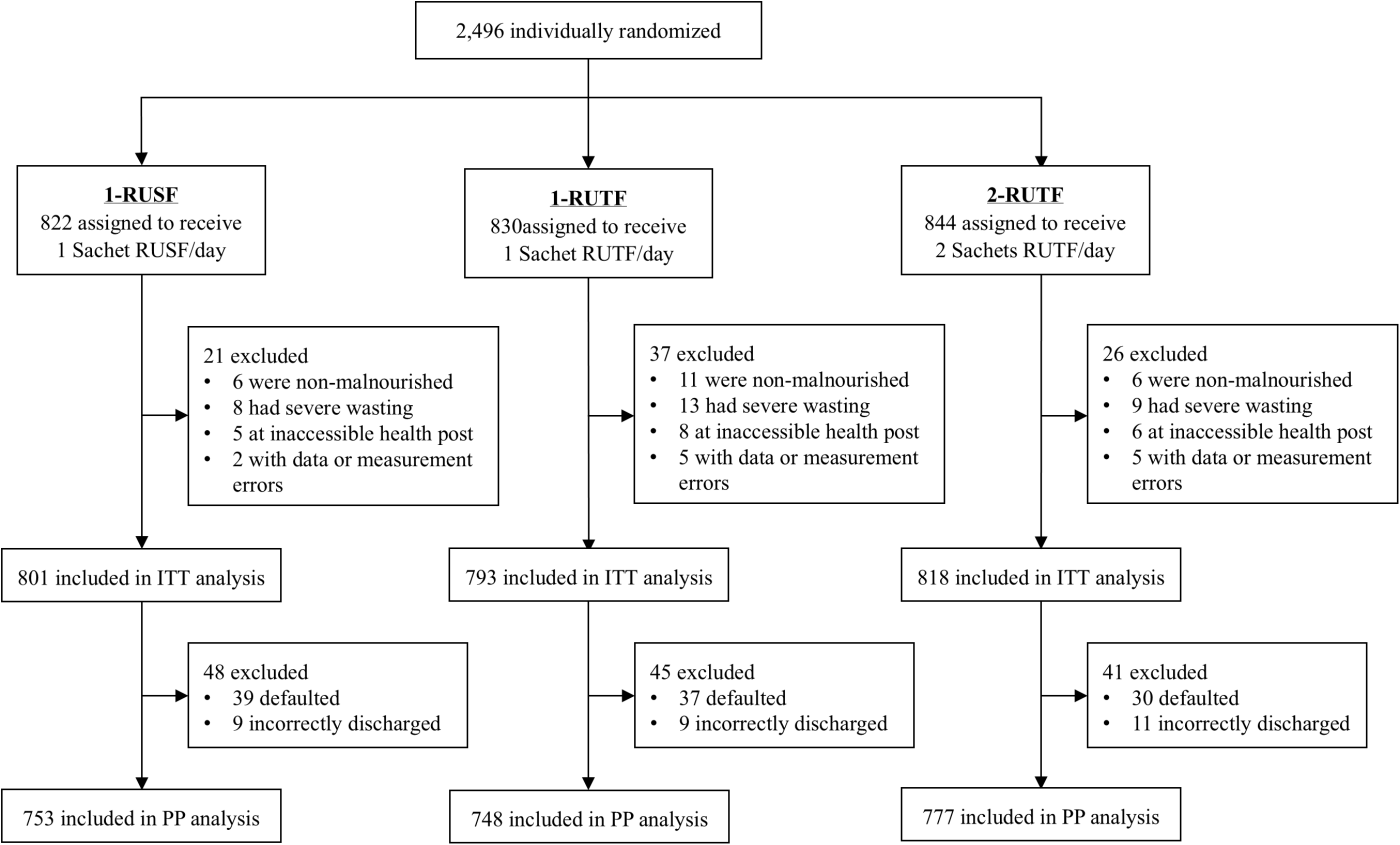
Participant flowchart. The diagram shows enrollment, randomization, exclusions, and inclusion in intention-to-treat (ITT) and per-protocol (PP) analyses across the three treatment groups (1-RUSF, 1-RUTF, and 2-RUTF). Default = absent for three consecutive visits. Incorrectly discharge = discharged with WHZ <−2 *or* MUAC <12.5 cm on either the discharge visit or the visit before discharge. ITT, intention to treat. PP, per protocol.

A total of 2,496 children were randomized between September 25, 2023, and August 30, 2024. Following exclusion due to non-eligibility, data errors, or the inability to complete study participation, 2,414 children were included in the final ITT analysis. Of these, 801 were randomized to the control group of one sachet of RUSF per day (1-RUSF), 793 were randomized to the intervention group of one sachet of RUTF per day (1-RUTF), and 818 were randomized to the intervention group of two sachets of RUTF per day (2-RUTF). PP analysis included 2,278 children (Fig 1).

At enrollment, 1,322/2,412 (55%) of participants were under 24 months of age (Table 1), with a median age of 21.1 months across all study arms. Sixty-two percent (1,482/2,412) of all children had a low MUAC at enrollment, while 1,558/2,412 (65%) had low WHZ. Forty-two percent (1,013/2,412) of participants were stunted on admission (HAZ <−2) and 727/2,412 (30%) were severely underweight (WAZ <−3). Additional enrollment characteristics are summarized in Tables 1 and S1.

**Table 1:** Enrollment characteristics of children upon admission to treatment for moderate acute malnutrition included in the intention-to-treat analysis, by treatment group.

	1-RUSF	1-RUTF	2-RUTF
Characteristic	*n* = 801	*n* = 793	*n* = 818
**Child Characteristics**			
Female	390 [48.7%]	406 [51.2%]	419 [51.2%]
Age (months)	20.8 [12.0, 35.5]	23.7 [12.1, 36.0]	19.9 [12.0, 35.3]
<24 months	449 [56.1%]	407 [51.3%]	476 [58.2%]
MUAC (cm)	12.3 [12.0, 12.8]	12.4 [12.1, 12.9]	12.3 [12.1, 12.8]
Height (cm)	76.4 [69.0, 87.3]	77.8 [70.5, 88.7]	75.6 [69.2, 88.4]
Weight (kg)	8.0 [6.9, 9.9]	8.2 [7.1, 10.3]	7.9 [6.9, 10.2]
WHZ	−2.19 [−2.50, −1.78]	−2.20 [−2.47, −1.84]	−2.15 [−2.46, −1.74]
HAZ	−2.05 [−3.02, −0.96]	−1.84 [−2.89, −0.67]	−1.90 [−2.96, −0.76]
WAZ	−2.55 [−3.18, −1.97]	−2.43 [−3.07, −1.77]	−2.45 [−3.09, −1.83]
**Health**			
Diarrhea in the previous 7 days	197 [24.7%]	185 [23.4%]	201 [24.6%]
Fever in the previous 7 days	228 [28.6%]	172 [21.8%]	183 [22.4%]
Cough in the previous 7 days	247 [31.0%]	210 [26.7%]	232 [28.4%]
Currently breastfed	407 [50.8%]	366 [46.2%]	371 [45.4%]
**Caregiver Characteristics**			
Primary caregiver is mother	774 [96.6%]	771 [97.2%]	791 [96.7%]
Mother has no formal education	613 [76.5%]	626 [78.9%]	641 [78.4%]
**Household Characteristics**			
Household currently displaced	28 [3.5%]	36 [4.5%]	20 [2.4%]
Head of household is female	93 [11.6%]	98 [12.4%]	105 [12.8%]
Number of children under 10 years old living at home	3 [2, 4]	3 [2, 4]	3 [2, 5]
Child is a twin	39 [4.9%]	23 [2.9%]	29 [3.6%]
Primary livelihood of household			
Agriculture	347 [43.3%]	308 [38.8%]	338 [41.3%]
Rearing livestock	38 [4.7%]	46 [5.8%]	45 [5.5%]
Mixed (livestock and agriculture)	41 [5.1%]	43 [5.4%]	61 [7.5%]
Casual labor	264 [33.0%]	256 [32.3%]	246 [30.1%]
Salaried work	33 [4.1%]	52 [6.6%]	42 [5.1%]
Other	74 [9.3%]	82 [10.4%]	81 [10.0%]
Enrolled in Gode District, Somali Region	403 [50.3%]	387 [48.8%]	405 [49.5%]
Enrolled in Teltele District, Oromia Region	398 [49.7%]	406 [51.2%]	413 [50.5%]

Data are *n* [%] or median [IQR], as appropriate.

MUAC, mid-upper arm circumference; WHZ, weight-for-height z-score; HAZ, height-for-age z-score; WAZ, weight-for-age z-score; RUSF, ready-to-use supplementary food; RUTF, ready-to-use therapeutic food.

When assessing enrollment characteristics disaggregated by *woreda* (S2 Table), notable differences were observed, namely that children enrolled in Gode were generally older (median 23.2 versus 18.1 months), taller (78.0 versus 75.4 cm), and heavier (8.2 versus 7.9 kg) than those in Teltele. Gode participants were also more likely to be enrolled by WHZ rather than MUAC (77.2% versus 52.3%), while Teltele had a higher proportion of children admitted by MUAC (75.0% versus 48.0%). Household livelihoods varied substantially, with most Teltele households relying on agriculture (64.8% versus 17.1%), whereas Gode households more often reported casual labor as their main source of income (52.0% versus 11.9%).

Enrollment characteristics for children in the PP analysis were similar to children in the ITT analysis and are provided by treatment group in S3 Table.

### Recovery, duration of therapy, and ration usage

**Fig 2:**
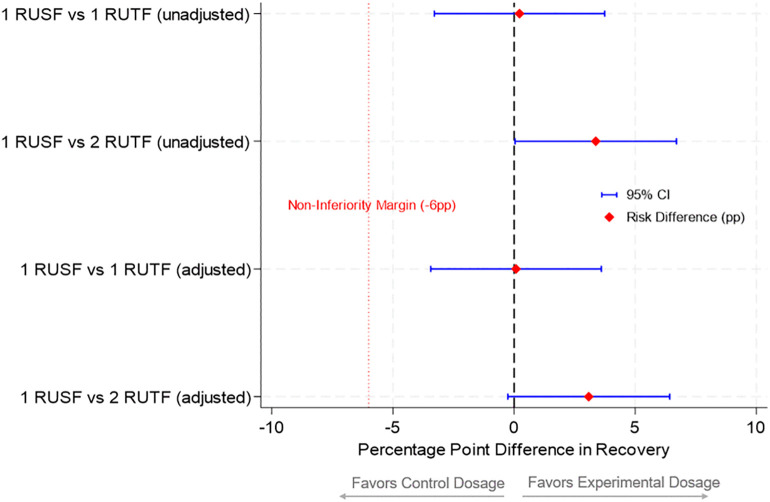
Forest plot of differences in recovery proportions between study arms in the intention-to-treat analysis, including after adjustment for enrollment variables. Points represent risk differences in recovery (percentage points) comparing each experimental group to the control (1-RUSF), and horizontal lines indicate 95% confidence intervals. The dashed vertical line indicates no difference, and the dotted vertical line indicates the pre-specified non-inferiority margin (−6 percentage points). Adjusted estimates account for baseline characteristic including age, sex, study location, MUAC, WHZ, WAZ, breastfeeding status, recent diarrhea, and the number of children under 10 years in the household. CI, confidence interval; RUTF, ready-to-use therapeutic food; RUSF, ready-to-use supplementary food; NI, non-inferiority margin; pp, percentage points.

In the ITT analysis, anthropometric recovery was achieved by 679/801 (84.8%) of children receiving 1-RUSF, 674/793 (85.0%) receiving 1- RUTF, and 721/818 (88.1%) receiving 2-RUTF (Table 2 and Fig 2). Recovery was 0.2 percentage points higher in the 1-RUTF group than in the control 1-RUSF group (95% CI [−3.3, 3.7]; *p* = 0.944) and 3.4 percentage points higher in the 2-RUTF group compared with the control 1-RUSF group (95% CI [0.0, 6.7]; *p* = 0.050). For both comparisons, the lower bound of the CI remained above the pre-specified −6-percentage-point non-inferiority margin, indicating that both experimental RUTF regimens were non-inferior to the standard RUSF regimen. In an exploratory post-hoc comparison between the two RUTF regimens, recovery was modestly higher among children receiving two sachets per day compared with one sachet per day (721/818 [88.1%] versus 674/793 [85.0%]; risk difference 3.1 percentage points, 95% CI [−0.2, 6.5]; *p* = 0.068).

**Table 2:** Primary outcome (proportion recovered) and secondary outcomes among all children treated for moderate acute malnutrition by treatment group in intention-to-treat and per protocol analyses.

	1-RUSF	1-RUTF	2-RUTF	1-RUSF vs. 1-RUTF		1-RUSF vs. 2-RUTF	
				Risk Difference	*p*-value*	Risk Difference	*p*-value*
Intention to Treat (ITT) Sample	*n* = 801	*n* = 793	*n* = 818				
Recovered	679 [84.8%]	674 [85.0%]	721 [88.1%]	0.2 [−3.3, 3.7]	0.944	3.4 [0.0, 6.7]	0.050
Not recovered	122 [15.2%]	119 [15.0%]	97 [11.9%]	−0.2 [−3.7, 3.3]	0.944	−3.4 [−6.7, 0.0]	0.050
Deteriorated to SAM	16 [2.0%]	13 [1.6%]	10 [1.2%]	−0.4 [−1.7, 1.0]	0.709	−0.8 [−2.0, 0.5]	0.240
Referred to hospital	6 [0.8%]	12 [1.5%]	16 [2.0%]	0.8 [−0.3, −1.8]	0.163	1.2 [0.1, 2.3]	0.051
Remained with MAM	59 [7.4%]	55 [6.9%]	39 [4.8%]	−0.4 [−3.0, 2.1]	0.771	−2.6 [−4.9, −0.3]	0.029
Defaulted	39 [4.9%]	37 [4.7%]	30 [3.7%]	−0.2 [−2.3, 1.9]	0.907	−1.2 [−3.2, 0.8]	0.268
Died	2 [0.3%]	2 [0.3%]	2 [0.2%]	0.0 [−0.5, 0.5]	1.000	0.0 [−0.5, 0.5]	1.000
Length of stay (d)	56 [42, 98]	56 [42, 84]	56 [28, 84]	0 [0, 0]	0.556	0 [0, 0]	0.008
Total sachets provided	70 [42, 98]	70 [42, 98]	112 [84, 196]	0 [0, 0]	0.446	−56 [−56, −56]	<0.001
**Per Protocol (PP) Sample**	***n* = 753**	***n* = 748**	***n* = 777**				
Recovered	670 [89.0%]	666 [89.0%]	710 [91.4%]	0.1 [−3.1, 3.2]	1.000	2.4 [−0.6, 5.4]	0.122
Not recovered	83 [11.0%]	82 [11.0%]	67 [8.6%]	−0.1 [−3.2, 3.1]	1.000	−2.4 [−5.4, 0.6]	0.122
Deteriorated to SAM	16 [2.1%]	13 [1.7%]	10 [1.3%]	−0.4 [−1.8, 1.0]	0.708	−0.8 [−2.1, 0.5]	0.238
Referred to hospital	6 [0.8%]	12 [1.6%]	16 [2.1%]	0.8 [−0.3, 1.9]	0.163	1.3 [0.1, 2.4]	0.051
Remained with MAM	59 [7.8%]	55 [7.4%]	39 [5.0%]	−0.5 [−3.2, 2.2]	0.770	−2.8 [−5.3, −0.4]	0.028
Died	2 [0.3%]	2 [0.3%]	2 [0.3%]	0.0 [−0.5, 0.5]	1.000	0.0 [−0.5, 0.5]	1.000
Length of stay (d)	56 [42, 98]	56 [37, 84]	56 [28, 84]	0 [0, 0]	0.552	0 [0, 0]	0.005
Total sachets provided	70 [42, 98]	70 [42, 98]	112 [84, 196]	0 [0, 0]	0.377	−56 [−56, −42]	<0.001

Data are *n* [%] or median [IQR] as appropriate. Effect estimates are presented as risk differences (percentage points) or median differences with 95% confidence intervals. Risk differences are calculated as experimental arm minus control (1-RUSF). Non-inferiority conclusions are based on confidence intervals relative to the pre-specified −6 percentage point margin.

RUTF, ready-to-use therapeutic food; RUSF, ready-to-use supplementary food; SAM, severe acute malnutrition; MAM, moderate acute malnutrition.

*P-values were calculated using Fisher’s exact test for binary outcomes (e.g., recovered/non-recovered, deteriorated to SAM, remained MAM, referred to hospital, defaulted, died) and Mann-Whitney U test for continuous variables (e.g., length of stay and total sachets provided). *P*-values are primarily shown for descriptive purposes. Non-inferiority conclusions are based on confidence intervals relative to pre-specified margins.

The most common reason for non-recovery was remaining moderately malnourished after 16 weeks of treatment, which occurred in 59/801 (7.4%), 55/793 (6.9%), and 39/818 (4.8%) of children in the 1-RUSF, 1-RUTF, and 2-RUTF groups, respectively. A modest difference in remaining malnourished was observed between the 1-RUSF and 2-RUTF groups (+2.6 percentage points in 1-RUSF, 95% CI [0.3, 4.9]; *p* = 0.029). Among the 153 children classified as remaining with MAM at week 16, 47 (31%) met the anthropometric recovery threshold at their final visit, but not for the required two consecutive visits. Other non-recovery sub-categories—including default, referral to hospital, deterioration to SAM, and death—were relatively uncommon and did not differ meaningfully across intervention arms (Table 2).

The median length of stay in the program was 56 days in all three groups, with similar interquartile ranges (Table 2). Although medians were identical, the distribution of treatment skewed right, with a subset of children remaining in treatment for extended periods. A higher proportion of children in the 2-RUTF arm recovered somewhat earlier in the course of treatment than those in the 1-RUSF and 1-RUTF arms, creating small but systematic differences in the upper tail of the distribution, which was visible in the time-to-event analysis (S1 Fig). However, the absolute differences are too small to have a practical impact on programmatic implementation. Given the comparable length of stay across groups, children in the 2-RUTF group received significantly more product, with a median consumption of 112 sachets, compared to 70 sachets in both the 1-RUSF and 1-RUTF groups (Table 2). Missed visits were uncommon overall: 2,027/2,412 (84.0%) of participants attended all scheduled visits; 219/2,412 (9.1%) missed one visit; and 166/2,412 (6.9%) missed two or more visits. The distribution of missed visits was similar across treatment arms and study sites.

Results estimated from the adjusted regression models (S4 Table) were not meaningfully different than the unadjusted findings (Table 2). Estimated recovery proportions differed from crude values by only 0.8–1.2 percentage points and the direction and magnitude of treatment effects were unchanged (S4 Table). Adjusted regression models yielded odds ratios of recovery of 1.01 (95% CI [0.76, 1.34]) for 1-RUTF versus 1-RUSF and 1.31 (95% CI [0.98, 1.76]) for 2-RUTF versus 1-RUSF, consistent with the small absolute differences observed in the primary analysis (S5 Table).

PP results were broadly consistent with the ITT analysis, albeit with slightly higher recovery proportions due to the exclusion of defaulters (67/753 [89.0%] for 1-RUSF, 666/748 [89.01%] for 1-RUTF, and 710/777 [91.4%] for 2-RUTF) (Table 2). Non-recovery subcategories, length of stay, and total sachets received all followed similar patterns to those observed in the ITT analysis.

### Growth outcomes

Growth trajectories, including changes in weight, MUAC, length, WHZ, HAZ, and WAZ, were clinically similar across the three study arms without any clear pattern of superiority of any of the MAM supplementation approaches tested (Table 3). Weight gain was highest during the first two weeks of treatment, with a median rate of 1.65 g/kg/d. Over the course of treatment, children gained a median of 0.5 cm in length and were discharged at a median MUAC of 13.0 cm and a median WHZ of −1.42, with comparable values across all study arms. HAZ scores declined slightly over the treatment period, resulting in greater stunting at discharge than enrollment.

**Table 3:** Growth outcomes among all children treated for moderate acute malnutrition by treatment group in intention-to-treat analysis.

	1-RUSF	1-RUTF	2-RUTF	1-RUSF vs. 1-RUTF	1-RUSF vs. 2-RUTF
	*n* = 801	*n* = 792	*n* = 818	Difference	Difference
**Weight**					
Change in weight from admission to discharge (kg)	0.7 [0.5, 1.0]	0.7 [0.4, 0.9]	0.7 [0.4, 1.0]	0.03 [0.00, 0.10]	0.00 [0.00, 0.08]
Rate of weight gain (g/kg/d)	1.43 [0.83, 2.32]	1.28 [0.72, 2.14]	1.41 [0.84, 2.42]	0.13 [0.03, 0.24]	−0.02 [−0.13, 0.09]
Rate of weight gain in the first 2 weeks (g/kg/d)	1.65 [0.00, 3.48]	1.47 [0.00, 3.00]	1.77 [0.09, 3.57]	0.12 [−0.05, 0.38]	−0.08 [−0.36, 0.09]
Rate of weight gain in the first 4 weeks (g/kg/d)	1.47 [0.57, 2.53]	1.41 [0.46, 2.48]	1.51 [0.53, 2.71]	0.07 [−0.07, 0.23]	0.00 [−0.17, 0.14]
**MUAC**					
Change in MUAC from admission to discharge (cm)	0.6 [0.3, 0.9]	0.6 [0.3, 0.9]	0.7 [0.4, 0.9]	0.0 [−0.0, 0.0]	0.0 [−0.1, 0.0]
Rate of MUAC gain (mm/d)	0.11 [0.05, 0.18]	0.11 [0.05, 0.18]	0.12 [0.06, 0.19]	0.0 [−0.01, 0.01]	−0.02 [−0.02, −0.00]
MUAC at discharge (cm)	13.0 [12.7, 13.4]	13.0 [12.7, 13.5]	13.0 [12.8, 13.5]	0.0 [−0.1, 0.0]	−0.1 [−0.1, 0.0]
**Length**					
Change in length from admission to discharge (cm)	0.5 [0.2, 1.1]	0.5 [0.2, 1.3]	0.5 [0.2, 1.1]	0.0 [−0.0, 0.0]	0.0 [0.0, 0.1]
Rate of length gain (mm/day)	0.09 [0.04, 0.18]	0.09 [0.04, 0.18]	0.09 [0.04, 0.19]	0.0 [−0.01, 0.01]	0.00 [−0.01, 0.01]
**WHZ**					
Change in WHZ from admission to discharge	0.76 [0.43, 1.16]	0.69 [0.37, 1.05]	0.73 [0.38, 1.13]	0.09 [0.03, 0.15]	0.03 [−0.03, 0.09]
WHZ at discharge	−1.40 [−1.70, −0.91]	−1.47 [−1.79, −1.03]	−1.39 [−1.71, −0.90]	0.09 [0.04, 0.15]	0.00 [−0.06, 0.06]
**HAZ**					
Change in HAZ from admission to discharge	−0.25 [−0.49, −0.11]	−0.22 [−0.48, −0.08]	−0.25 [−0.47, −0.09]	−0.03 [−0.06, 0.0]	−0.02 [−0.05, 0.01]
HAZ at discharge	−2.42 [−3.36, −1.34]	−2.14 [−3.20, −0.97]	−2.24 [−3.33, −1.14]	−0.28 [−0.44, −0.12]	−0.17 [−0.33, −0.01]
Proportion discharged with HAZ ≥ −2	320 [40.0%]	374 [47.2%]	359 [43.9%]	7.2 [2.4, 12.1]	0.0 [−0.0, 0.1]
**WAZ**					
Change in WAZ from admission to discharge	0.37 [0.14, 0.64]	0.34 [0.11, 0.58]	0.37 [0.12, 0.65]	0.04 [0.00, 0.08]	0.00 [−0.04, 0.04]
WAZ at discharge	−2.15 [−2.77, −1.57]	−2.10 [−2.71, −1.44]	−2.05 [−2.73, −1.40]	−0.08 [−0.17, 0.01]	−0.11 [−0.20, −0.02]
Proportion discharged with WAZ ≥ −3	642 [80.2%]	661 [83.4%]	679 [83.0%]	0.0 [−0.0, 0.1]	0.0 [−0.0, 0.1]

Data are *n* [%] or median [IQR] as appropriate. Outcomes reflect change from admission to discharge unless otherwise specified. Effect estimates are presented as risk difference (percentage points) for binary outcomes and median differences for continuous outcomes, with 95% confidence intervals. Risk differences are calculated as experimental arm minus control (1-RUSF) and were estimated using the Hodges-Lehmann method, which calculates the median of all pairwise differences between groups, with values reflecting distributional shifts rather than simple differences in medians.

MUAC, mid-upper arm circumference; WHZ, weight-for-height z-score; HAZ, height-for-age z-score; WAZ, weight-for-age z-score; RUTF, ready-to-use therapeutic food; RUSF, ready-to-use supplementary food.

**Fig 3:**
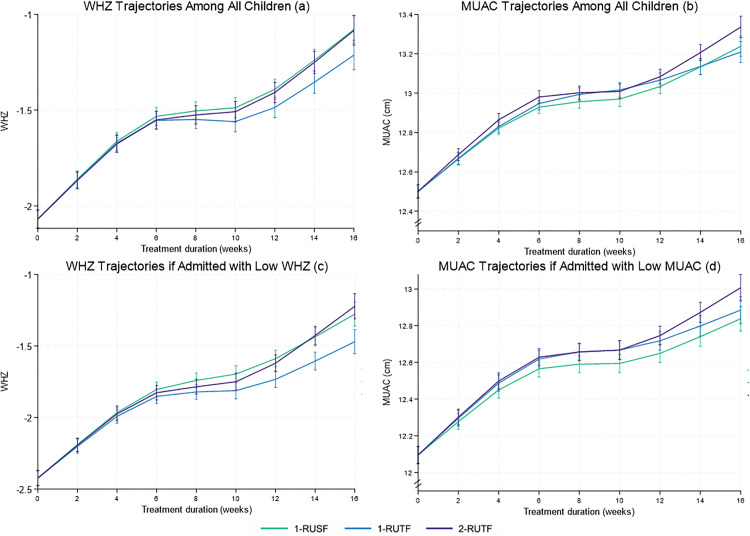
Adjusted anthropometric trajectories during treatment, by study arm. **(a)** WHZ trajectories and **(b)** mid–upper arm circumference (MUAC) trajectories over the treatment period in the overall sample. **(c)** WHZ trajectories and **(d)** MUAC trajectories among children with anthropometric deficits at admission (WHZ <−2 or MUAC <12.5 cm). Lines represent adjusted mean trajectories, and error bars indicate 95% confidence intervals. Estimates are derived from mixed-effects models with flexible (non-linear) functions of time. Models were adjusted for age, sex, study location, baseline anthropometry, breastfeeding status, recent diarrhea, and household composition. MUAC, mid–upper arm circumference; WHZ, weight-for-height z-score; RUTF, ready-to-use therapeutic food; RUSF, ready-to-use supplementary food.

Results from regression-adjusted growth models (S6 Table) were consistent with unadjusted results. Fig 3 shows highly similar adjusted trajectories of WHZ and MUAC over the course of treatment across study arms in both the overall sample and low-admission subgroups, with comparable slopes over time and no clear differences in rates of anthropometric recovery. Analysis of growth velocity conditional on nutritional status also yielded consistent findings across arms (S2 Fig) and when considering only children who recovered from MAM (S7 Table).

### Outcomes by WHO prioritization criteria

The strength of association between enrollment characteristics and treatment outcomes differed substantially across WHO-defined prioritization criteria of age, MUAC, and WAZ (Table 4). Younger children (6–23 months) had a modestly lower proportion recovered than older children (24–59 months) (1,122/1,332 [84.2%] versus 952/1,080 [88.2%]). However, they also demonstrated higher rates of weight gain during treatment, particularly in the first two weeks (1.6 versus 1.2 g/kg/day).

**Table 4:** Outcomes among all children treated for moderate acute malnutrition disaggregated by enrollment characteristics in intention-to-treat analysis.

	Age			MUAC			WAZ		
	6–23 mo	24–59 mo	*p*-value*	11.5–11.9 cm	≥12.0 cm	*p*-value*	WAZ <−3	WAZ ≥−3	*p*-value*
	*n* = 1,332	*n* = 1,080		*n* = 355	*n* = 2,057		*n* = 727	*n* = 1,685	
Recovered	1,122 [84.2%]	952 [88.2%]	0.007	268 [75.5%]	1,806 [87.8%]	<0.001	618 [85.0%]	1,456 [86.4%]	0.371
Not recovered	210 [15.8%]	128 [11.9%]	0.007	87 [24.5%]	251 [12.2%]	<0.001	109 [15.0%]	229 [13.6%]	0.371
Deteriorated to SAM	29 [2.2%]	10 [0.9%]	0.015	15 [4.2%]	25 [1.3%]	<0.001	13 [1.8%]	26 [1.5%]	0.725
Referred to hospital	30 [2.3%]	4 [0.4%]	<0.001	13 [3.7%]	21 [1.0%]	0.001	15 [2.1%]	19 [1.1%]	0.089
Remained with MAM	80 [6.0%]	73 [6.8%]	0.451	44 [12.4%]	109 [5.3%]	<0.001	43 [5.9%]	110 [6.5%]	0.649
Defaulted	66 [5.0%]	40 [3.7%]	0.162	14 [3.9%]	92 [4.5%]	0.779	33 [4.5%]	73 [4.3%]	0.829
Died	5 [0.4%]	1 [0.1%]	0.233	1 [0.3%]	5 [0.2%]	1.000	5 [0.7%]	1 [0.1%]	0.011
Length of stay (d)	56 [42, 84]	56 [28, 98]	0.663	70 [42, 112]	56 [28, 84]	<0.001	56 [42, 84]	56 [28,84]	0.104
Total sachets provided	84 [56, 112]	84 [56, 112]	0.372	98 [70, 126]	84 [56, 112]	<0.001	84 [56, 112]	84 [56, 112]	0.639
Weight gain (kg)	0.7 [0.4, 0.9]	0.8 [0.5, 1.0]	<0.001	0.8 [0.4, 1.1]	0.7 [0.4, 1.0]	0.003	0.8 [0.5, 1.0]	0.7 [0.4, 1.0]	<0.001
Rate of weight gain (g/kg/d)	1.59 [0.86, 2.57]	1.19 [0.74, 1.91]	<0.001	1.61 [0.84, 2.47]	1.36 [0.80, 2.27]	0.055	1.66 [1.00, 2.61]	1.28 [0.73, 2.15]	<0.001
Rate of weight gain in the first 2 weeks (g/kg/d)	1.93 [0.00, 3.83]	1.35 [0.00, 2.80]	<0.001	2.07 [0.02, 3.90]	1.57 [0.00, 3.25]	0.009	2.01 [0.61, 4.02]	1.37 [0.00, 3.04]	<0.001
MUAC gain (cm)	0.7 [0.3, 0.9]	0.6 [0.4, 0.9]	0.887	1.0 [0.8, 1.2]	0.6 [0.3, 0.8]	<0.001	0.7 [0.4, 1.0]	0.6 [0.3, 0.9]	0.062
MUAC at discharge (cm)	12.8 [12.6, 13.1]	13.3 [12.9, 13.9]	<0.001	12.7 [12.5, 12.9]	13.0 [12.8, 13.5]	<0.001	12.9 [12.7, 13.2]	13.0 [12.7, 13.6]	<0.001
Length gain (cm)	0.5 [0.2, 1.2]	0.5 [0.2, 1.1]	0.078	0.7 [0.3, 1.5]	0.5 [0.2, 1.1]	<0.001	0.5 [0.2, 1.2]	0.5 [0.2, 1.2]	0.954
Change in WHZ	0.81 [0.37, 1.24]	0.66 [0.40, 0.96]	<0.001	0.92 [0.31, 1.48]	0.70 [0.39, 1.07]	<0.001	0.94 [0.51, 1.33]	0.65 [0.35, 1.02]	<0.001
WHZ at discharge	−1.21 [−1.64, −0.72]	−1.58 [−1.78, −1.26]	<0.001	−1.08 [−1.67, −0.64]	−1.45 [−1.73, −1.01]	<0.001	−1.38 [−1.74, −0.90]	−1.43 [−1.73, −0.96]	0.407
Change in HAZ	−0.41 [−0.68, −0.23]	−0.13 [−0.23, −0.02]	<0.001	−0.42 [−0.77, −0.19]	−0.22 [−0.44, −0.09]	<0.001	−0.20 [−0.43, −0.05]	−0.26 [−0.49, −0.11]	<0.001
HAZ at discharge	−2.53 [−3.40, −1.49]	−1.88 [−3.05, −0.65]	<0.001	−3.05 [−3.98, −2.15]	−2.12 [−3.14, −0.99]	<0.001	−3.72 [−4.45, −3.12]	−1.64 [−2.43, −0.64]	<0.001
Change in WAZ	0.36 [0.04, 0.68]	0.36 [0.18, 0.56]	0.585	0.44 [0.02, 0.79]	0.35 [0.13,0.59]	0.012	0.50 [0.22, 0.75]	0.32 [0.08, 0.55]	<0.001
WAZ at discharge	−2.11 [−2.71, −1.52]	−2.09 [−2.77, −1.37]	0.405	−2.43 [−3.07, −1.84]	−2.05 [−2.68, −1.40]	<0.001	−3.02 [−3.42, −2.63]	−1.76 [−2.20, −1.24]	<0.001

Data are *n* [%] or median [IQR], as appropriate.

*P-values were calculated using Fisher’s exact test for binary outcomes (e.g., recovered/non-recovered, deteriorated to SAM, remained MAM, referred to hospital, defaulted, died) and Mann-Whitney U test for continuous variables (e.g., length of stay, total sachets provided, weight-gain, etc.). *P*-values are provided for descriptive purposes only to aid interpretation of patterns across subgroups. No adjustment was made for multiple comparisons, and results should be interpreted cautiously.

MUAC, mid-upper arm circumference; WHZ, weight-for-height z-score; HAZ, height-for-age z-score; WAZ, weight-for-age z-score; SAM, severe acute malnutrition; MAM, moderate acute malnutrition.

MUAC at admission was more strongly associated with outcomes during treatment. Children with lower MUAC (11.5–11.9 cm) had a lower proportion recovered than those with MUAC ≥12.0 cm (268/355 [75.5%] versus 1,806/2,057 [87.8%]), and were more likely to deteriorate to SAM, be referred to the hospital, or remain with moderate malnutrition at discharge. Despite poorer recovery, children with lower MUAC experienced greater r early weight gain (2.1 versus 1.6 g/kg/day) and larger absolute anthropometric gains during treatment.

WAZ at admission was not strongly associated with overall recovery (618/727 [85.0%] versus 1,456/1,685 [86.4%] for WAZ <–3 versus ≥–3). Mortality was higher among severely underweight children (0.7% versus 0.1%), although the absolute numbers were very small (5 children versus 1 child), and therefore these findings should be interpreted with caution. Severely underweight children also experienced higher early weight gain (2.0 versus 1.4 g/kg/day) during treatment.

**Fig 4:**
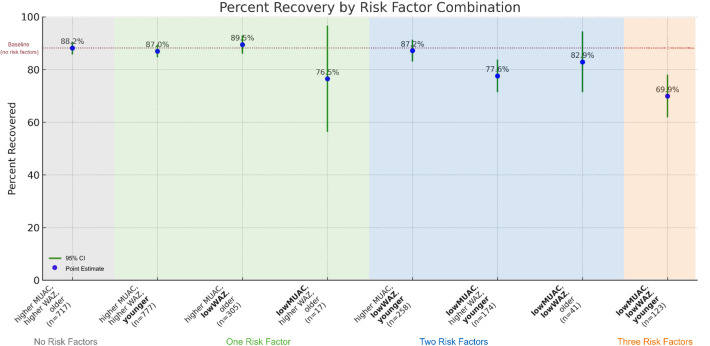
Recovery rates by combination of risk factors at enrollment. Points represent the proportion of children who recovered within each subgroup defined by combinations of enrollment characteristics, and error bars indicate 95% confidence intervals. Subgroups are categorized by number of risk factors present (none, one, two, or three), where risk factors include lower MUAC (<12.0 cm), lower WAZ (<−3), and younger age (6–23 months) at enrollment. The dashed horizontal line represents the recovery rate among children with no risk factors. “Higher MUAC” was defined as MUAC ≥ 12 cm and “lower MUAC” as MUAC 11.5–11.9 cm. “Higher WAZ” was defined as WAZ ≥ −3 and “lower WAZ” as WAZ <−3. “Older” was defined as ≥ 24 months old at enrollment and “younger” as 6–23 months old at enrollment.

We next examined whether recovery varied according to the cumulative burden of prioritization criteria, or “risk factors” present at enrollment. Among children with no pre-specified risk factor at admission (*i.e.*, older age at 24–59 months, higher MUAC at ≥12.0, and higher WAZ at ≥−3), the proportion recovered was 88.2% (95% CI [85.8%, 90.5%]) (Fig 4). Recovery remained high among children with isolated younger age (87.0%) or isolated low WAZ (89.6%) in the absence of other risk factors. In contrast, children with low MUAC without other risk factors had a markedly lower proportion recovered of 76.5% (95% CI [56.3%, 96.6%]), although the sample size for this group was smaller.

Recovery was even lower when two risk factors were present. For example, children with both low MUAC and younger age had a substantially lower proportion recovered of 77.6% (95% CI [71.4%, 83.78%]). Children with *all three* risk factors—age 6–23 months, MUAC 11.5–11.9 cm, and WAZ <−3—had the lowest observed recovery, at 69.9% (95% CI [61.8%, 78.0%]). Across combinations, low MUAC emerged as the strongest and most consistent predictor of failure to recover, whereas age and WAZ provided comparatively limited additional prognostic information.

### Effect measure modification

Among children aged 6–23 months, recovery was higher in the 2-RUTF group (416/476 [87.4%]) than the 1-RUTF group (333/407 [81.8%]), corresponding to an absolute difference of 5.6 percentage points (95% CI: 0.8–10.4), while recovery proportions were similar across treatment arms among children aged 24–59 months (S8 Table). Younger children in the 2-RUTF arm also had lower proportions remaining with MAM at discharge than children receiving either 1-RUSF or 1-RUTF. Although subgroup analyses identified differences between treatment groups within the younger age subgroup formal interaction testing found no statistical evidence that age modified the treatment effect (S12 Table). Growth trajectories were also broadly similar across intervention arms when stratified by age (S8b Table).

Differences in recovery and growth outcomes by treatment arm within MUAC strata were modest and not clinically meaningful (S9 Table). Among children with MUAC ≥ 12.0 cm, recovery was slightly higher in the 2-RUTF group compared to both 1-RUTF and 1-RUSF, but the absolute differences were small (S9 Table). No meaningful differences were observed among children with MUAC 11.5–11.9 cm, although the smaller sample size in this subgroup may have limited precision of estimates. Consistent with these findings, interaction testing found no statistical evidence that treatment effects differed by MUAC category, despite the poorer overall outcomes observed among children with lower MUAC (Tables 4 and S12).

Among children with WAZ ≥ –3, recovery was higher in the 2-RUTF group (516/578 [89.3%]) compared to both 1-RUSF (453/535 [84.7%]) and 1-RUTF (487/572 [85.1%]), with additional benefits in weight gain and WHZ gain (S10 Table). In contrast, among children with WAZ < –3, recovery and growth outcomes were similar across arms, with no statistically significant differences observed. Despite the within-group differences observed among children with WAZ ≥ –3, interaction testing showed no evidence that treatment effects differed between WAZ categories (S12 Table).

Subgroup results within each *woreda* largely mirrored the overall study findings. However, the overall recovery was slightly lower in Teltele compared to Gode (1,030/1,217 [84.0%] versus 1,044/1,195 [87.4%]; S11 Table). Among children who did not recover, Teltele had a significantly higher proportion of non-response (117/1,217 [9.6%] versus 36/1,195 [3.0%]), while Gode had a significantly higher proportion of default (74/1,195 [6.2%] versus 32/1,217 [2.6%]). Treatment effects across the three study arms were similar in direction and magnitude at each site, with overlapping confidence intervals for risk differences (S11 Table). Formal interaction testing found no evidence that treatment effects differed by study site (S12 Table).

## Discussion

In this individually randomized, investigator-blinded, controlled trial, we found that two different RUTF regimens—one sachet per day (1-RUTF) and two sachets per day (2-RUTF)—were non-inferior to the *de facto* standard regimen of one sachet of RUSF per day (1-RUSF) for the management of MAM. Recovery proportions, length of stay, and growth outcomes were highly similar across all three groups, and are consistent with the 2023 WHO recommendation that either LNS formulation can be used for MAM supplementation [7]. Although the higher-dose RUTF regimen (2-RUTF) resulted in slightly higher recovery and marginally faster time to recovery, the absolute differences were small in magnitude and resulted in the use of 1.6 times more product than both the 1-RUSF and 1-RUTF groups. In an exploratory *post hoc* comparison between the two RUTF regimens, recovery was modestly higher with the double-dose regimen, but the difference was small and not statistically significant. Taken together, these findings provide no clear evidence that increasing the daily RUTF dose improves treatment outcomes. Short-term growth indicators similarly showed only limited benefit from increased RUTF dosage. Given the substantially greater product requirements associated with the higher-dose regimen, our results do not support the routine use of two sachets of RUTF per day for MAM management.

Several hypotheses may explain why larger differences in outcomes were not observed between the low and high RUTF dosage study arms. First, the higher-dose regimen may have increased the likelihood of household sharing, reducing the amount actually consumed by the index child—an effect observed in prior MAM and SAM treatment studies [28–30]. Second, the additional RUTF may have displaced some home foods, resulting in no net increase in total energy intake. This is particularly relevant for smaller children, for whom two sachets of RUTF per day resulted in substantially higher kcal/kg/day than physiologically required, as shown in S13 Table, and far exceeds the 40%–60% of nutrient needs that WHO recommends are to be met through specialized nutritious foods [7]. In such cases, excess supplementation may simply surpass physiological intake capacity rather than accelerate recovery or growth. These findings also align with several recent SAM and MAM trials that demonstrated comparable recovery and growth outcomes despite large dosage reductions [15,16], suggesting that beyond a certain threshold, additional calories confer diminishing marginal benefit. Finally, it is possible that factors unrelated to total caloric intake—such as inflammation, immune status, or gut microbiota composition—may play a notable role in determining recovery trajectories. These biological mechanisms were not assessed in this trial and remain important areas for future research.

Our findings are consistent with prior trials evaluating different formulations or dosing strategies for MAM treatment [8,31], which have generally shown that once children receive any form of appropriate supplementary food, relatively minor changes within the product formulation or dosage yield limited additional benefits. For example, a 2017 study in Burkina Faso observed no meaningful differences in recovery, non-response, and risk of deterioration to SAM when comparing products with different soy quality and varying levels of milk-derived protein [32]. Similarly, a 2019 trial in Sierra Leone found nearly equivalent proportion recovered amongst children receiving Super Cereal Plus with amylase (65%), Corn-Soy Blend Plus with oil (64%), Corn-Soy-Whey Blend with oil (62%), and RUSF (62%) [33].

With regards to LNS comparisons, the ComPAS trial in Kenya and South Sudan tested an experimental protocol for treating both MAM and SAM but found no significant differences in the proportion recovered between children receiving RUTF or RUSF, when stratified by admissions nutritional status [15]. Finally, a 2,019 trial in Malawi comparing two milk-based RUSFs of different protein quality found similar proportions of recovery, rates of weight and MUAC gain, and time to recovery in children receiving either product [34]. These patterns are further supported by a recent systematic review and meta-analysis, which found little difference in recovery between FBFs (with enhanced micronutrient and/or milk content) and LNS [12].

Consistent with these findings, our trial found no significant and practical differences in the proportion recovered among children receiving RUSF or varying doses of RUTF. Together, this body of evidence suggests that the provision of an adequate, nutrient-dense supplement itself may be more critical than minor variations in product formulations or dosing to achieve recovery for most children in the management of MAM. Given the many logistical and financial challenges involved in supplying LNS products for MAM and SAM, we conclude that either RUSF or RUTF should be considered equivalently effective for supplementing children with MAM and that the choice of product can be guided by supply chain and pragmatic decisions.

Unlike most prior MAM studies, which defined recovery on the basis of MUAC *or* WHZ, and sometimes for only one single visit, our trial required children to meet both MUAC ≥12.5 cm and WHZ ≥–2 with no edema for two consecutive visits, consistent with updated WHO guidelines [7]. This more stringent, multidimensional definition may increase the time that children remain classified as MAM despite clinically meaningful improvements, particularly among those who present with discordant indicators. Thus, the relatively higher non-response proportion observed here, as compared to previous MAM studies [35,36], may reflect these new recovery criteria rather than true biological non-response. As a large multicenter trial conducted using the updated WHO recommended diagnostic and recovery criteria for AM, these findings add important context to the emerging evidence base.

Results from our subgroup analyses also provide timely evidence to inform the recent WHO 2023 recommendation regarding specially formulated foods being prioritized for children with MAM when resources are constrained. Of the eight risk factors listed in the guideline, we had sufficient data to assess the first three individually and in combination: MUAC 11.5–11.9 cm, WAZ <–3, and age <24 months. In our analyses, children admitted with lower MUAC were significantly less likely to recover than those with higher MUAC values. This threshold (MUAC < 12.0 cm) identifies a particularly vulnerable subgroup, including many of whom are younger children since MUAC is not adjusted for age and therefore disproportionately selects those under 24 months [37,38]. These findings contrast with recent results from Mali, where low MUAC did not strongly differentiate recovery outcomes among children with MAM receiving supplementation in a MUAC-only program [39]. One plausible explanation is that MUAC-only admission criteria reduce heterogeneity in enrollment MUAC, attenuating observable differences in response across MUAC strata. In our trial—where children entered with either low MUAC or WHZ—enrollment anthropometric variability was greater and differences in recovery by MUAC were more pronounced. We did not investigate whether a different MUAC threshold would better stratify risk.

We found that younger age was only a weak predictor of recovery. While younger children had slightly lower recovery proportions than older children, the difference was small and largely explained by their overlap with the low MUAC group. This aligns with findings from the Mali cohort which similarly observed limited differences in recovery between younger and older children when both groups received LNS supplementation [39,40]. Combined with existing evidence showing no difference in mortality risk across age groups among children with wasting [41], these findings do not support the age-focused approach that has gained recent backing [42,43].

Similarly, low WAZ did not correlate with recovery, either alone or in combination with other risk factors. While low WAZ was associated with higher mortality in our cohort—indicating that it captures an important dimension of clinical vulnerability—this relationship did not translate into improved responsiveness to supplementary feeding in our trial. Lack of association with recovery contrasts with a previous analysis showing lower odds of recovery amongst moderately wasted children with WAZ <−3, when treatment intensity varied widely across programs [44]. That pooled analysis showed children with low WAZ benefited most when receiving “high-intensity” or “mid-intensity” LNS, compared with fortified flour or energy-dense home foods. In our trial, all children received therapeutic or supplementary doses of LNS, potentially reducing observed differences in recovery by WAZ. This interpretation is supported by findings from the large Mali cohort of moderately wasted children receiving supplemental doses of RUTF, which also reported no meaningful difference in recovery between those with low and higher WAZ [39].

As with WHO guideline on this matter [7], it is important to emphasize that our results reflect treatment response among children receiving SFFs and do not speak directly to the risk of deterioration or mortality in the absence of treatment [18]. Where available, longitudinal follow-up from untreated cohorts would be most informative for guiding decisions about which children should be prioritized to receive SFFs in resource-limited settings. Across such studies, low MUAC consistently emerges as one of the strongest predictors of near-term mortality, reinforcing its value as a targeting criterion for SFFs [45–47]. Based on our findings from individual and combined-risk factor analyses, results from untreated cohorts, and operational feasibility, MUAC alone appears to be the most practical and relevant indicator for identifying children with MAM who are more likely to experience poor outcomes with age and WAZ providing comparatively limited prognostic value. Similar findings were recently observed from a multi-country cohort of children with MAM in West and Central Africa treated using a MUAC-only based screening and treatment protocol [48].

Despite differences in overall outcomes across several risk-factor strata, we found no evidence that age, MUAC, or WAZ modified the relative effectiveness of the three supplementation regimens. Although some subgroup analyses suggested modest advantages of the higher-dose RUTF regimen among younger children or children with higher WAZ, formal interaction testing did not support differential treatment effects across any of the prespecified subgroups. These findings suggest that WHO higher-risk criteria may be useful for identifying children at greater risk of poor outcomes, but do not appear useful for selecting among alternative supplementary feeding regimens.

Although children with a lower MUAC had a higher risk of non-recovery; they also exhibited faster MUAC and weight gain, indicating robust physiological response. This suggests that these children are responding well to supplementation, but may require either more time to reach pre-defined recovery thresholds, a higher dosage of supplementary food, or an alternative indicator to better assess treatment response. Illustratively, children with low MUAC receiving a higher dose of RUTF (2-RUTF) experienced lower proportions of non-response (i.e., failing to meet the discharge criteria within the maximum length of stay) compared with those receiving 1-RUSF; however, subgroup analyses showed no statistical evidence that the treatment effects differed by MUAC category. Moreover, increasing the dose or extending the length of stay would introduce protocol complexity and increase resource demands, with potential implications for program cost. Indeed, recent evidence suggests that the recovery thresholds for these smallest children may not accurately reflect what is actually a comparable level of nutritional recovery [49]. An alternative strategy under discussion in some programming contexts is to integrate MAM children with particularly low MUAC into outpatient SAM treatment protocols by raising MUAC threshold for SAM admission from 11.5 to 12.0 cm [50–52].

This study has several notable strengths. The individually randomized, multi-site design minimized potential sources of bias and enhanced the generalizability of findings across diverse programmatic settings. The trial enrolled a heterogeneous population of children across multiple regions in Ethiopia, spanning a range of agroecological zones, socioeconomic contexts, and anthropometry. For example, the relatively high levels of consumption of camel milk in Somali Region is associated with lower incidence of stunting and provides context for pastoralist communities [53]. In particular, the consistency of results across such diversity in sites supports the external validity of our findings to other high-burden contexts in sub-Saharan Africa. With a large sample size and a low non-inferiority margin, the trial was sufficiently powered to detect even modest differences in recovery outcomes across treatment arms, allowing for robust comparisons. Importantly, we also address a key evidence gap highlighted in WHO’s 2023 guidelines by directly comparing both the type and dosage of LNS for the management of MAM.

Some study limitations should be noted. First, the intensive community screening and regular mass mobilizations used to support study recruitment likely led to earlier identification of MAM cases then would occur in routine program conditions, arguably skewing the enrolled participants towards the less wasted end of the MAM spectrum, which may not be representative of a typical program setting. While this may influence *absolute* recovery proportions observed, it is unlikely to affect *relative* differences between study arms given the results from the stratified MUAC analysis showed similar treatment responses across all regimens, even amongst children enrolled with MUAC 11.5–11.9 cm. Additionally, the trial evaluated only a single formulation of RUSF, despite substantial variation in product composition across manufacturers [54,55]. However, given the growing body of evidence showing that small differences in product composition rarely translate to meaningful differences in recovery among MAM outpatients, this is unlikely to have impacted the findings significantly. Finally, we did not collect biological markers or body composition assessments, which could have provided insight into whether changes in weight reflected differential accretion of fat versus lean mass, particularly in the higher-dose RUTF arm. This could be a valuable area for future research.

Our study provides robust randomized trial evidence to support the interchangeable use of one sachet of RUSF or RUTF in the management of MAM. This evidence supports increased flexibility for programs to make context-specific decisions based on whichever product is more readily available or less costly to maximize program efficiency without compromising effectiveness. Furthermore, our study concludes that while a relatively higher dose of RUTF results in small improvements in outcomes that do not justify the use of substantially larger amounts of supplementary products.

## Supporting information

S1 Table Additional enrollment characteristics of children included in the intention-to-treat analysis, by treatment group. Data are *n* (%) or median (IQR), as appropriate. Indented rows show the distribution of anthropometric measures among children within each subgroup at admission. MUAC, mid-upper arm circumference; WHZ, weight-for-height z-score; HAZ, height-for-age z-score; WAZ, weight-for-age z-score; RUSF, ready-to-use supplementary food; RUTF, ready-to-use therapeutic food.
(DOCX)

S2 Table Enrollment characteristics of children included in the intention-to-treat analysis, by study site. Data are *n* [%] or median [IQR], as appropriate. Characteristics were measured at enrollment and are presented by study site. Differences between sites were estimated using the Hodges-Lehmann method, which calculates the median of all pairwise differences between groups; values reflect distributional shifts, rather than simple differences in medians. MUAC, mid-upper arm circumference; WHZ, weight-for-height z-score; HAZ, height-for-age z-score; WAZ, weight-for-age z-score.
(DOCX)

S3 Table Enrollment characteristics of children included in the per-protocol population, by treatment group. Data are *n* [%] or median [IQR], as appropriate. Characteristics were measured at enrollment prior to initiation of treatment. MUAC, mid-upper arm circumference; WHZ, weight-for-height z-score; HAZ, height-for-age z-score; WAZ, weight-for-age z-score; RUSF, ready-to-use supplementary food; RUTF, ready-to-use therapeutic food.
(DOCX)

S4 Table Regression-adjusted primary outcomes among children treated for moderate acute malnutrition, by treatment group in intention-to-treat analysis. Values are adjusted percentages or means with 95% confidence intervals derived from regression models. Binary outcomes were modeled using logistic regression and continuous outcomes using Poisson or linear models, adjusting for child age, sex, anthropometry, breastfeeding status, diarrhea, children in the household, and study location. All values are model-based estimates, and in some cases, the 95% confidence intervals may include values outside the plausible range (e.g., negative percentages) due to statistical uncertainty at very low outcome probabilities. Differences represent adjusted contrasts between study arms and are defined as experimental arm − control arm (1-RUSF). RUTF, ready-to-use therapeutic food; RUSF, ready-to-use supplementary food; SAM, severe acute malnutrition; MAM, moderate acute malnutrition.
(DOCX)

S5 Table Adjusted odds ratios for recovery among all children treated for moderate acute malnutrition (*n* = 2,338 observations). Values are adjusted odds ratios with 95% confidence intervals derived from logistic regression models. Models were adjusted for prespecified enrollment covariates, including age, sex, district, baseline anthropometry (MUAC, WHZ, WAZ), breastfeeding status, recent diarrhea, and number of children under age 10 in the household. Only intervention effect estimates are presented, consistent with recommendations for reporting adjusted analyses in randomized trials. ^a^Reference group: 1-RUSF.
(DOCX)

S6 Table Regression-adjusted growth outcomes among children treated for moderate acute malnutrition by treatment group in intention-to-treat analysis. Values are adjusted means or proportions with 95% confidence intervals derived from regression models. Continuous outcomes were modeled using linear regression and binary outcomes using logistic regression, adjusting for child age, sex, anthropometry, breastfeeding status, diarrhea, children in the household, and study location. All values are model-based estimates. Differences represent adjusted contrasts between study arms on the outcome scale (e.g., kg, cm, z-score units, or percentage points) and are defined as experimental arm minus control (1-RUSF). MUAC, mid-upper arm circumference; WHZ, weight-for-height z-score; HAZ, height-for-age z-score; WAZ, weight-for-age z-score; RUTF, ready-to-use therapeutic food; RUSF, ready-to-use supplementary food.
(DOCX)

S7 Table Growth outcomes among children *recovered* from moderate acute malnutrition by treatment group in intention-to-treat analysis. Data are *n* [%] or median [IQR], as appropriate. Differences are presented as median difference with 95% confidence intervals and were estimated using the Hodges–Lehmann method, which calculates the median of all pairwise differences between groups. Values reflect distributional shifts, not simple differences in medians. Risk difference is defined as experimental arm minus control (1-RUSF). MUAC, mid-upper arm circumference; WHZ, weight-for-height z-score; HAZ, height-for-age z-score; WAZ, weight-for-age z-score; RUTF, ready-to-use therapeutic food; RUSF, ready-to-use supplementary food.
(DOCX)

S8 Table (a) Primary outcomes for children treated for moderate acute malnutrition, stratified by age group in intention-to-treat analysis. Data are *n* [%] or median [IQR], as appropriate. Effect estimates are presented as risk differences (percentage points) or median differences with 95% confidence intervals. Risk differences are calculated as experimental arm minus control (1-RUSF). Results are stratified by age group and should be interpreted as sub-group analyses. MAM, moderate acute malnutrition; SAM, severe acute malnutrition; RUTF, ready-to-use therapeutic food; RUSF, ready-to-use supplementary food. **(b) Growth indicators for children treated for moderate acute malnutrition, stratified by age group in intention-to-treat analysis.** Data are median [IQR]. Differences are presented as median difference with 95% confidence intervals. Differences are defined as experimental arm minus control (1-RUSF). Results are stratified by age group and should be interpreted as sub-analyses. MUAC, mid-upper arm circumference; WHZ, weight-for-height z-score; HAZ, height-for-age z-score; WAZ, weight-for-age z-score; RUTF, ready-to-use therapeutic food; RUSF, ready-to-use supplementary food.
(DOCX)

S9 Table (a) Primary Outcomes for children treated for moderate acute malnutrition, stratified by MUAC on admission in intention-to-treat analysis. Data are *n* [%] or median [IQR], as appropriate. Effect estimates are presented as risk differences (percentage points) or median differences with 95% confidence intervals. Risk difference is calculated as experimental arm minus control (1-RUSF). Results are stratified by MUAC at admission and should be interpreted as sub-analyses. MAM, moderate acute malnutrition; SAM, severe acute malnutrition; RUTF, ready-to-use therapeutic food; RUSF, ready-to-use supplementary food. **(b) Growth indicators for children treated for moderate acute malnutrition, stratified by MUAC on admission in intention-to-treat analysis.** Data are median [IQR]. Differences are presented as median difference with 95% confidence intervals. Differences are defined as experimental arm minus control (1-RUSF). Results are stratified by MUAC at admission and should be interpreted as sub-analyses. MUAC, mid-upper arm circumference; WHZ, weight-for-height z-score; HAZ, height-for-age z-score; WAZ, weight-for-age z-score; RUTF, ready-to-use therapeutic food; RUSF, ready-to-use supplementary food.
(DOCX)

S10 Table (a) Primary Outcomes for children treated for moderate acute malnutrition, stratified by WAZ on admission in intention-to-treat analysis. Data are *n* [%] or median [IQR], as appropriate. Effect estimates are presented as risk differences (percentage points) or median differences with 95% confidence intervals. Risk difference is calculated as experimental arm minus control (1-RUSF). Results are stratified by WAZ at admission and should be interpreted as sub-analyses. MAM, moderate acute malnutrition; SAM, severe acute malnutrition; RUTF, ready-to-use therapeutic food; RUSF, ready-to-use supplementary food. **(b) Growth indicators for children treated for moderate acute malnutrition, stratified by WAZ on admission in intention-to-treat analysis.** Data are median [IQR]. Differences are presented as median difference with 95% confidence intervals. Differences are defined as experimental arm minus control (1-RUSF). Results are stratified by WAZ at admission and should be interpreted as sub-analyses. MUAC, mid-upper arm circumference; WHZ, weight-for-height z-score; HAZ, height-for-age z-score; WAZ, weight-for-age z-score; RUTF, ready-to-use therapeutic food; RUSF, ready-to-use supplementary food.
(DOCX)

S11 Table (a) Outcomes among children treated for moderate acute malnutrition by study site across all treatment arms in intention-to-treat analysis. Data are *n* (%) or median (IQR), as appropriate. Differences are presented as risk differences (percentage points) or median differences with 95% confidence intervals and are calculated as Gode minus Teltele. MUAC, mid-upper arm circumference; WHZ, weight-for-height z-score; HAZ, height-for-age z-score; WAZ, weight-for-age z-score; MAM, moderate acute malnutrition; SAM = severe acute malnutrition. **(b) Primary Outcomes for children treated for moderate acute malnutrition, stratified by study site in intention-to-treat analysis.** Data are *n* [%] or median [IQR], as appropriate. Effect estimates are presented as risk differences (percentage points) or median differences with 95% confidence intervals. Risk difference is calculated as experimental arm minus control (1-RUSF). Results are stratified by study site and should be interpreted as sub-analyses. MAM, moderate acute malnutrition; SAM, severe acute malnutrition; RUTF, ready-to-use therapeutic food; RUSF, ready-to-use supplementary food. **(c) Growth indicators for children treated for moderate acute malnutrition, stratified by study site in intention-to-treat analysis.** Data are median [IQR]. Differences are presented as median difference with 95% confidence intervals. Differences are defined as experimental arm minus control (1-RUSF). Results are stratified by study site and should be interpreted as sub-analyses. MUAC, mid-upper arm circumference; WHZ, weight-for-height z-score; HAZ, height-for-age z-score; WAZ, weight-for-age z-score; RUTF, ready-to-use therapeutic food; RUSF, ready-to-use supplementary food.
(DOCX)

S12 Table Tests for interaction between treatment arm and sub-groups on recovery. Interaction tests were conducted using logistic regression models with recovery as the dependent variable and including treatment arm, subgroup indicators, and treatment-by-subgroup interaction terms. *P*-values were obtained from joint Wald tests of the interaction terms. Subgroup-specific treatment effect estimates and outcome distributions are presented in S8–S11 Tables. MUAC, mid-upper arm circumference; WAZ, weight-for-age z-score.
(DOCX)

S13 Table Estimated contribution of study rations (RUSF and RUTF) to recommended daily energy requirements among children with moderate acute malnutrition. Values are estimated using midpoints of each weight range and assumed energy requirements of 100–130 kcal/kg/day, consistent with international guidance for children with moderate acute malnutrition. The target contribution of supplementary foods is assumed to be approximately 40%–60% of total daily energy requirements. Energy content per sachet was assumed to be 535 kcal for RUSF and 500 kcal for RUTF. These values are illustrative and intended to contextualize dosing strategies rather than represent precise individual requirements. RUTF, ready-to-use therapeutic food; RUSF, ready-to-use supplementary food; kcal, kilocalories.
(DOCX)

S1 Fig Time to recovery among all children treated for moderate acute malnutrition by treatment group. Kaplan–Meier curves show time to recovery by treatment group. Differences between treatment groups were assessed using log-rank tests. Numbers at risk are shown below the figure. Log-rank: across all groups, *p* = 0.015; between 1 sachet RUSF/day vs. 1 sachet RUTF/day, *p* = 0.620; between 1 sachet RUSF/day vs. 2 sachets RUTF/day, *p* = 0.006; between 1 sachet RUTF/day vs. 2 sachets RUTF/day, *p* = 0.028. RUTF, ready-to-use therapeutic food; RUSF, ready-to-use supplementary food.
(DOCX)

S2 Fig Growth velocity by baseline MUAC and treatment group. Panel A shows weight gain velocity (g/kg/day) and Panel B shows MUAC gain velocity (mm/week), each plotted against MUAC at the start of the visit interval. Lines represent smoothed estimates of growth velocity across the observed range of MUAC values using locally weighted regression (LOESS), with shaded bands indicating 95% confidence intervals. MUAC, mid–upper arm circumference; RUTF, ready-to-use therapeutic food; RUSF, ready-to-use supplementary food.
(DOCX)

S1 Checklist CONSORT 2025 checklist. Completed Consolidated Standards of Reporting Trials (CONSORT) 2025 checklist indicating where each recommended reporting item is addressed within the manuscript. Hopewell S, Chan AW, Collins GS, Hróbjartsson A, Moher D, Schulz KF, et al. CONSORT 2025 Statement: updated guideline for reporting randomised trials. BMJ. 2025; 388:e081123. https://dx.doi.org/10.1136/bmj-2024-081123. 2025 Hopewell et al. This is an Open Access article distributed under the terms of the Creative Commons Attribution License (https://creativecommons.org/licenses/by/4.0/), which permits unrestricted use, distribution, and reproduction in any medium, provided the original work is properly cited.
(DOCX)

S2 Checklist Inclusivity in global research checklist. Completed Inclusivity in Global Research Questionnaire describing ethical considerations, authorship, and local collaboration and engagement.
(DOCX)

S1 File Study Protocol. Published study protocol [19].
(PDF)

## Abbreviations

AI: artificial intelligence
AM: acute malnutrition
CIs: confidence intervals
DSMB: Data and Safety Monitoring Board
FBFs: fortified blended flours
HAZ: height-for-age z-score
HEWs: Health Extension Workers
IQRs: interquartile ranges
ITT: intention-to-treat
LNS: lipid-based nutrient supplements
LOS: length of stay
MAM: moderate acute malnutrition
MODAM-MAM: Modified Dosages for Acute Malnutrition MAM
MUAC: mid-upper arm circumference
PP: per-protocol
RD: risk difference
RUSF: ready-to-use supplementary food
RUTF: ready-to-use therapeutic food
SAM: severe acute malnutrition
SFFs: specially formulated foods
TSFP: targeted supplementary feeding program
UNICEF: United Nations Children’s Fund
VIFs: variance inflation factors
WHO: World Health Organization
WHZ: weight-for-height z-score
