# Supplementary material for: Comparative effectiveness of lipid-nutrient spread supplementation regimens for moderate acute malnutrition in Ethiopia: The MODAM-MAM individually randomized controlled trial
Source: PLoS Med. 2026 Aug 11;23(8):e1005199. doi: 10.1371/journal.pmed.1005199 (PMC13475968; doi:10.1371/journal.pmed.1005199)
Supplement: Supplementary file 1 [file pmed.1005199.s018.pdf]

Trehan et al. BMC Nutrition (2025) 11:71
https://doi.org/10.1186/s40795-025-01054-w

BMC Nutrition

STUDY PROTOCOL

Open Access

The Modified Dosages for Acute Malnutrition (MODAM) study: protocol for three integrated randomized controlled trials of novel approaches for the management of childhood wasting in Ethiopia

Indi Trehan[1*], Yosef Beyene[2], Hiwot Darsene[3], Bailey S. Adams[4], Maria Wrabel[4], Getu Gizaw[5], Liya A. Legese[5], Bernardette Cichon[6], Stanley Chitekwe[7], Mesfin W. Shellemew[7], Masresha Tessema[2] and Heather C. Stobaugh[4*]

Abstract

Background Only a small percentage of children with severe and moderate acute malnutrition receive treatment due to resource limitations, relatively complex treatment protocols, persistent supply chain challenges, and limited early identification among high-risk populations. Several innovations to the current model of care for uncomplicated acute malnutrition have been proposed, including modified doses of nutritional supplementation and family-led mid-upper-arm circumference (MUAC) and edema screening ("Family MUAC") for early identification. The evidence base for these innovations remains limited.

Methods The Modified Dosages for Acute Malnutrition (MODAM) study includes three integrated individually randomized clinical trials testing innovations in the identification and treatment of acute malnutrition in Ethiopia. One trial will enroll 2400 children aged 6–59 months with severe acute malnutrition, testing standard weight-based dosing of ready-to-use therapeutic food (RUTF) against two experimental RUTF dosing regimens: either two sachets (1000 kcal) daily of RUTF until discharge, or two sachets until achieving anthropometric criteria for moderate acute malnutrition (MAM), at which time dosing will be decreased to one sachet (500 kcal) daily until discharge as fully recovered. A second trial will enroll 2400 children with MAM and test a standard dose of one daily sachet (540 kcal) of ready-to-use supplemental food against two experimental dosing regimes: one sachet (500 kcal) or two sachets (1000 kcal) of RUTF daily until discharge. Children who recover from these two trials will be randomized again into a third trial evaluating post-recovery protocols designed for the early identification of relapse: (1) the control arm involving one scheduled return visit at 24 weeks post-recovery; (2) the first intervention arm involving three

*Correspondence:
Indi Trehan
indi@alum.berkeley.edu
Heather C. Stobaugh
hstobaugh@actionagainsthunger.org

Full list of author information is available at the end of the article

(BMC logo retracted)

© The Author(s) 2025. **Open Access** This article is licensed under a Creative Commons Attribution 4.0 International License, which permits use, sharing, adaptation, distribution and reproduction in any medium or format, as long as you give appropriate credit to the original author(s) and the source, provide a link to the Creative Commons licence, and indicate if changes were made. The images or other third party material in this article are included in the article's Creative Commons licence, unless indicated otherwise in a credit line to the material. If material is not included in the article's Creative Commons licence and your intended use is not permitted by statutory regulation or exceeds the permitted use, you will need to obtain permission directly from the copyright holder. To view a copy of this licence, visit http://creativecommons.org/licenses/by/4.0/.

scheduled return visits at 4, 12, and 24 weeks post-recovery; and (3) the second intervention arm which involves caregivers receiving Family MUAC training and one scheduled visit at 24 weeks post-recovery.

Discussion This study will provide data on the effectiveness of multiple innovations in the management of childhood acute malnutrition. Results will add to the evidence base on the effectiveness and cost-effectiveness of such modifications in the identification and management of acute malnutrition, ideally adding to the global database on this topic and directly contributing to future WHO guidelines.

Trial registration Trials were registered on clinicaltrials.gov as NCT06038071 (registered September 8, 2023), NCT06056089 (registered September 20, 2023), and NCT06061484 (registered September 24, 2023).

Keywords Wasting, Acute malnutrition, Mid-upper arm circumference, Simplified approaches, Post-recovery monitoring, Relapse

Background

Acute malnutrition (AM), also known as "wasting" with or without nutritional edema, remains a pressing global health challenge that continues to profoundly impact the survival and optimal growth and development of children throughout low- and middle-income countries [1]. Despite decades of attention and innovations in the identification, diagnosis, and management of AM, an estimated 45 million children under five years of age, or 6.8% of children this age worldwide, at any given point in time still suffered from this scourge in 2022 [2]. While the terminology suggests an "acute" problem new to the child, the overwhelming majority of children that suffer from AM are chronically undernourished with insufficient dietary quality and quantity, often experiencing repeated bouts of acute and chronic infections. Indeed, while AM can develop quite quickly, the adjective "acute" rather refers to the effectiveness of acute management for these children that can significantly decrease the high risk of near-term mortality [1].

AM is defined and diagnosed by basic anthropometry that can be performed in low-resource settings either at the household or village level using relatively simple tools. While the condition encompasses a range of severity, it is divided into two categories associated with risk of mortality among children aged 6–59 months. Severe acute malnutrition (SAM) is defined as having a weight-for-length Z-score (WLZ) more than 3 standard deviations below the WHO Growth Standards [3] reference median, and/or a mid-upper arm circumference (MUAC) less than 115 mm, and/or nutritional bilateral pitting edema (without an alternative etiology for the edema such as cardiac, renal, or hepatic dysfunction) [4]. Moderate acute malnutrition (MAM) is defined as having a WLZ between 2 and 3 standard deviations below the WHO Growth Standards reference median, and/or a MUAC of 115–124 mm, without edema. AM may be further classified as "complicated", warranting hospital admission in a small minority of cases, or "uncomplicated" which can be treated in an outpatient setting using a package of care known as the community-based management of acute malnutrition (CMAM).

CMAM demonstrates tremendous success in decreasing mortality in children with AM. The effectiveness of CMAM, along with several important updates, was reaffirmed by the World Health Organization (WHO) in 2023 after a multiyear process rigorously re-evaluating the evidence base for numerous aspects of CMAM [5]. Despite these successes and increased attention given to AM over the last 20 years by the international community, there persists a large gap between the number of children suffering from AM and the number who receive treatment [6]. The reasons for this coverage gap are multifactorial and include insufficient financial and human resources and challenges with identification and diagnosis of malnourished children.

These coverage gaps have led to an important movement in reconsidering aspects of the CMAM approach that may increase cost-efficiency, improve early identification, reduce risk of relapse, and potentially provide treatment to more children. A number of these adaptations, often referred to as "simplified approaches", have been piloted and implemented during supply shortages, clinical trials, and as adaptations during the COVID-19 pandemic [7–11].

Clinical trials testing the effectiveness of these approaches compared to standard approaches have generally shown favorable outcomes, although not universally so in all settings and for all important outcomes, and have used inconsistent methodologies that arguably limit their widespread applicability. There may also be local contextual factors that might impact the effectiveness of these novel approaches in all settings. The integration of multiple approaches together in the same CMAM program have also not been studied at scale. There thus remains a pressing need for additional rigorous clinical trials testing the effectiveness and cost-effectiveness of innovations in the identification and management of acutely malnourished children, especially in new locations.

In 2022, the Federal Ministry of Health in Ethiopia made a request for local evidence on simplified approaches to inform revisions to the Ethiopia national AM guidelines, including alignment with the global-level WHO wasting guidelines revised in 2023 [5], highlighting the need for additional evidence on efficient dosing and innovations in the management of AM, particularly in emergency settings due to increasing conflicts and environmental shocks. Recent surveys in Ethiopia have demonstrated a critically high level of AM in rural areas of the country [12], along with more than 1/3 of recovered children relapsing to AM after initial recovery [13–15].

A consortium of Ethiopian and international partners is thus implementing the Modified Dosages for Acute Malnutrition (MODAM) study, described here. After a review of the literature and formative research involving input from an external technical advisory group in addition to numerous national and international stakeholders, a series of three integrated randomized controlled clinical trials have been designed. These will test:

1. modified doses of ready-to-use therapeutic foods for children with SAM;
2. modified doses of ready-to-use supplemental and therapeutic foods for children with MAM; and.
3. innovative approaches to monitor children after recovery from AM in an effort to improve post-discharge outcomes such as early diagnosis of relapse [16, 17].

These trials are designed to provide essential information to inform policies and program designs by key decision-makers among national ministries of health, international stakeholders (e.g. UNICEF, WHO), implementing organizations, and academics about the utility of several novel interventions in the identification and management of childhood AM in high-burden contexts.

Methods

Study setting

All three trials will be performed with an integrated approach, using a team of trained field researchers supported by Action Against Hunger USA who will work to support CMAM programs operating at health posts and health centers operated by the Federal Ministry of Health. Health posts are the lowest level of health care in Ethiopia, closest to their communities but also the least resourced. Meanwhile, health centers are one level higher in the hierarchy, providing both primary care as well as referral care for patients sent from health posts. Study subjects will be drawn from communities with relatively high endemic rates of food insecurity and malnutrition but have also suffered recent shocks including those from climate change and conflict.

Quarterly community mobilization activities at the local village level will help to disseminate information to the populations who live in the catchment areas of the research sites to help identify children with possible AM. Enrollment and follow-up activities will take place weekly at the health posts and health centers. Each health post and health center will be supplied with food products (RUTF and RUSF), measuring instruments, amoxicillin, and albendazole throughout the course of the study to ensure that stockouts and insufficient supplies are not a factor in trial outcomes.

Study population

Children who reside in the catchment areas of the individual health posts and health centers will be eligible for enrollment if they are 6–59 months of age at the time of screening, are diagnosed with uncomplicated AM (including passing an appetite test if diagnosed with SAM), have no plans to leave the area over the next year, and have caregiver consent for enrollment.

Children will be excluded from the study if they have been treated for AM, either as inpatients or outpatients, within the past 3 months. This also means that children who are transferred from inpatient care to outpatient care to continue their therapy at the health post level will be excluded from the research studies but will be assisted in continuing to receive routine outpatient CMAM care per the national treatment protocols. Children with known chronic illnesses such as severe cerebral palsy, congenital anomalies such as unrepaired congenital heart disease, Down syndrome, hydrocephalus, unrepaired cleft lip or palate, or other conditions that might be expected to contribute to challenges with feeding the allocated interventions will also be excluded. Ineligible children who are identified to have uncomplicated AM will still receive care at the health posts without prejudice using the standard national protocols.

Anthropometry

Study subjects will be evaluated and enrolled through a series of steps outlined below to ensure consistency and equity for all children encountered. Children's AM status will be assessed using standard field anthropometric techniques [18]. At screening, enrollment, each treatment follow-up visit, and each post-recovery follow-up visit, all of the following will be evaluated:

- recumbent length using a rigid board, to the nearest 1 mm;
- weight using a digital scale, to the nearest 100 g;
- MUAC using a flexible tape, to the nearest 1 mm; and.
- bilateral pitting edema.

Length and weight will then be evaluated using gender-specific look-up tables to determine their WLZ based on WHO Growth Standards [3]. Children will be classified as having SAM if their WLZ is more than 3 standard deviations below the median, if their MUAC is less than 115 mm, or if they demonstrate bilateral pitting edema.

Children will be classified as having MAM if their WLZ is between 3 and 2 standard deviations below the median or if their MUAC is ≥ 115 and < 125 mm, without meeting other criteria for SAM.

Clinical assessment

Children will also be assessed for complications that may warrant referral to a higher level of care. These include, but are not limited to, moderate-to-severe dehydration, altered mental status, lethargy or listlessness, hypothermia, respiratory distress, poor peripheral perfusion, repeated vomiting, and inability to feed their assigned study food under observation at the research site. Children with any evidence of such complications will be excluded from the study and instead be referred for care at a health center or hospital.

Test feeding

After diagnosis of uncomplicated SAM, children will undergo a test feeding with ready-to-use therapeutic food (RUTF) under direct supervision by a member of the research team and the post's health extension worker (HEW). Children will be expected to complete approximately 30 g of RUTF while at the health post to be considered to have successfully passed the feeding test as a final evaluation of their suitability for outpatient management of AM [19].

CMAM counseling

Standard CMAM counseling will be provided to caregivers about the proper consumption of their nutritional supplementation, including the need to consume the daily allotment at multiple small feedings through the course of the day prior to feeding home foods, the need to continue breastfeeding (if still breastfeeding), the need for availability of clean water while feeding, sanitation and hygiene reminders, the importance of not sharing the supplements with siblings or others, and signs of clinical worsening that would warrant an urgent return to the health post or health center.

To improve adherence to the assigned intervention group and decrease the risk of crossover, additional counseling will be provided to caregivers regarding the importance of respecting the randomization process and the feasible safety of all intervention groups. Scripting in the field will emphasize the nature of the interventions as *medicine* (rather than food) customized for each individual child's specific illness, based on their severity of acute malnutrition and randomization group. At each follow-up visit, the total number of sachets consumed will be asked of the caregivers and empty sachets will be collected from the caregiver. Informal discussions with local village health care workers, community leaders, and individual caregivers have all suggested that the caregivers in the study areas will understand, and be accepting of, randomization that may lead to varying amounts of intervention foods to each child.

Ethics and consent

The study received ethical approval from the Ethiopia Public Health Institute and the University of Washington. After initial screening, anthropometry, clinical assessment, test feeding, and CMAM counseling, caregivers of children who are deemed eligible for entry into the MODAM study, either with SAM or MAM, will be provided with written and verbal information about the entire study, including the enrollment process, randomization, treatment protocols, follow-up visits, and the post-recovery study. An opportunity will be given to ask any questions that arise. Consent will be sought verbally and recorded with either a written signature or thumbprint, depending on caregiver literacy. Eligible children who do not consent to the study will continue to receive care without prejudice using the standard national protocols.

Randomization and masking

For those children who are ultimately eligible and consent to the study, randomization will then take place using separate randomization blocks for children enrolled in the 2 AM treatment trials (SAM and MAM). In each of the two treatment trials, there are three study arms to which children will be individually randomized.

Randomization will occur in blocks of 18 (6 sets of allocations to each of the 3 arms) using small opaque envelopes placed into a larger letter-sized opaque envelope. Each small opaque envelope will contain a code corresponding to one of the intervention arms. The consenting caregiver for the child will be asked to reach into the large envelope and randomly select one small envelope, which will then be opened in front of the caregiver in private to reveal the coded intervention group allocated. This coded information will also be recorded on the child's paper data cards in the field and eventually entered into the study electronic database.

Given the nature of the interventions, it will not be possible to mask the allocated intervention to enrolled children's caregivers; however, anthropometric assessments, outcome assessments, and all statistical analyses will remain masked to the allocated intervention arms until all analyses are complete.

Enrollment questionnaire

In addition to randomization, an enrollment questionnaire will be completed for all children, which will include questions for the caregiver on a range of topics that may be related to trial outcomes. These will include topics such as the child's past medical history,

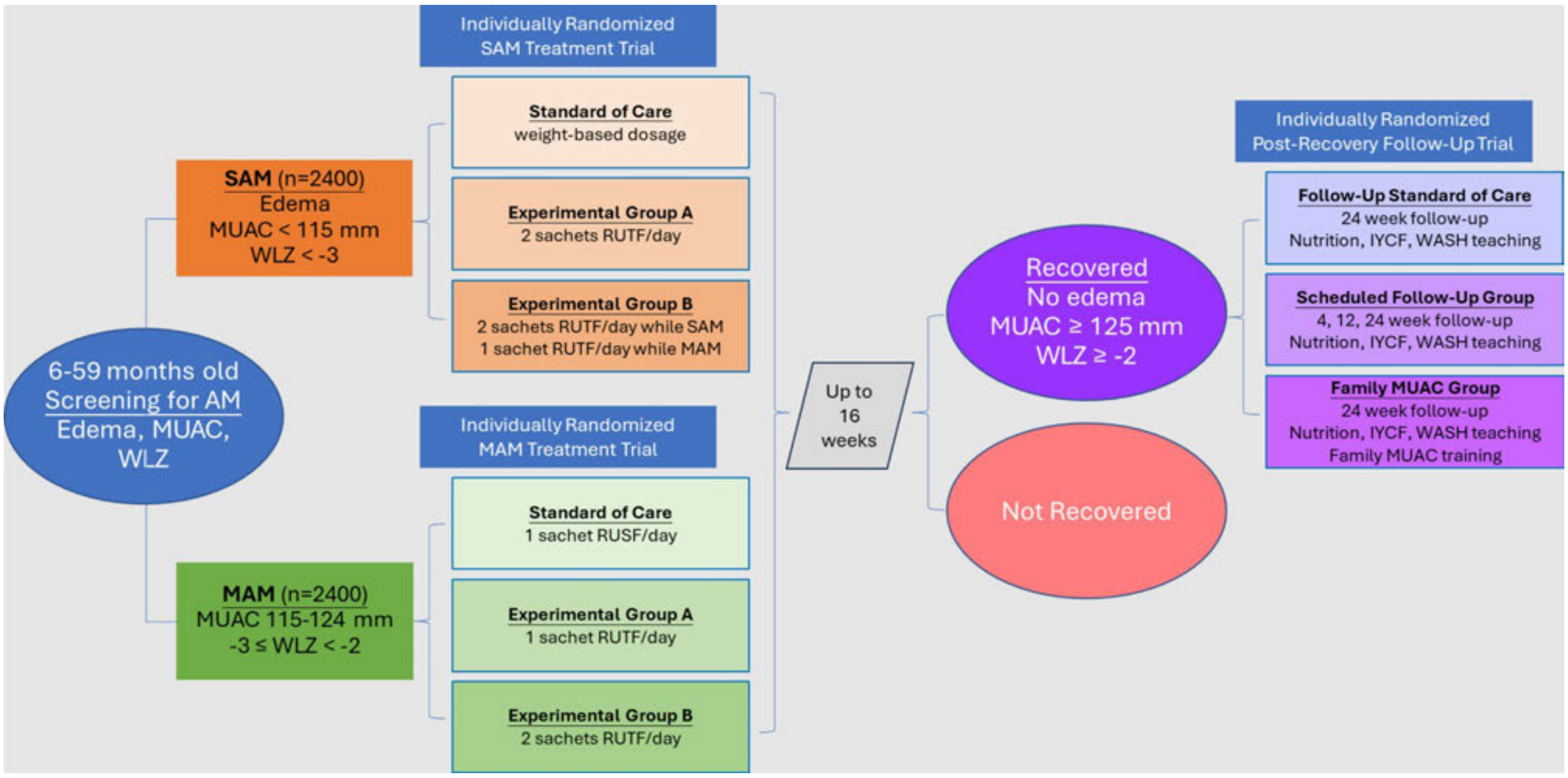

Fig. 1 Patient flow through 3 individually randomized clinical trials encompassed by the MODAM study

Table 1 Weight-based dosing of 500-kcal RUTF sachets for children in the standard of care group

Weight (kg)	Sachets per day	Sachets per week
**3.5–3.9**	1½	11
**4.0–5.4**	2	14
**5.5–6.9**	2½	18
**7.0–8.4**	3	21
**8.5–9.4**	3½	25
**9.5–10.4**	4	28
**10.5–11.9**	4½	32
**≥ 12**	5	35

brief information about the child's breastfeeding status, prior history of AM, demographic information about the household, household food security, and an economic evaluation that asks about household goods and livestock ownership.

Severe acute malnutrition intervention– trial 1

Upon enrollment, children with SAM will be randomly allocated to one of three groups (Fig. 1), whereby they will receive varying doses of 500 kcal sachets of RUTF provided in 7-day rations on a weekly basis until discharge. All aspects of treatment are identical across the arms apart from the dosing of RUTF, which differs as follows:

- SAM standard of care (control) group: Children in this group will be dosed with approximately 150–200 kcal of RUTF per kg of the child's weight per day (Table 1), reflecting the current national standard of care in Ethiopia [20]. In this dosing regimen, as the child's weight increases over the course of treatment, so too does the amount of RUTF that is provided each week.
- SAM experimental group A: Children in this group will be dosed with two sachets of RUTF to consume daily until discharge. This reflects the draft simplified emergency protocol in Ethiopia and a common choice for novel protocols in many settings. In addition to the possibility of improved cost-effectiveness with this experimental dosing, the simplicity of a consistent dose each day could make it easier for health workers to implement, improve understanding among caregivers for how to ration a week's worth of RUTF accurately, and improve the predictability of demand forecasting.
- SAM experimental group B: Children in this group will be dosed with two sachets of RUTF to consume daily while *any* of their anthropometric parameters are in the SAM range (i.e., WLZ < – 3, MUAC < 115, or edema). Children will then transition to one sachet of RUTF to consume daily when *all* of their anthropometric parameters are in the MAM range or better (i.e., WLZ ≥ -3, MUAC ≥ 115, and no edema). Similar dosing regimens have been previously trialed and piloted in other locations [21], with the same potential advantages of experimental group A. This dosing regimen also has the theoretical advantage of providing a higher dose of macronutrients and micronutrients while the child is most malnourished and subsequently decreasing the treatment dosage as the child improves– the opposite approach to the control arm. This dosing regimen is hypothesized to be even more cost effective than the experimental group A regimen, but runs the risk of lower recovery rates and higher rates of relapse that should be tested.

Table 2 Follow-up visit outcomes for AM intervention trials

SAM Trial	MAM Trial	Definition
**Interim Status**		
Continued SAM	–	Anthropometry demonstrates ongoing SAM
Improved to MAM	–	Anthropometry demonstrates improvement to MAM
–	Continued MAM	Anthropometry demonstrates ongoing MAM
**Final Outcome = Recovered**		
Recovered	Recovered	Anthropometry has reached recovery criteria for 2 consecutive visits within 16 weeks of enrollment
**Final Outcome = Not Recovered**		
–	Deteriorated to SAM	Anthropometry worsened from MAM to SAM since previous visit
Died	Died	Death since previous visit
Hospitalization	Hospitalization	Child hospitalized for medical or nutritional deterioration
Lost to follow-up	Lost to follow-up	Child unable to be located for three consecutive visits
Remained with AM	Remained with AM	Child has not achieved recovery criteria after 16 weeks of therapy

Moderate acute malnutrition intervention– trial 2

Upon enrollment, children with MAM will be randomly allocated to one of three groups (Fig. 1), with either RUTF or ready-to-use supplementary food (RUSF) (depending on the arm) provided in a 14-day supply on a fortnightly basis until discharge. All aspects of treatment will remain identical across arms apart from the dosing and type of ready-to-use food provided, which will differ as follows:

- MAM standard of care (control) group. Children in this group will be dosed with one sachet of RUSF, providing 530–540 kcal, to consume daily until discharge. This reflects the current national standard of care in Ethiopia [20].
- MAM experimental group A. Children in this group will receive one sachet of RUTF to consume daily until discharge. This reflects the draft simplified emergency protocol for MAM treatment in Ethiopia. Testing this dosing regimen in a rigorous trial will provide local evidence about the safety, efficacy, and cost efficiency of this regimen for food insecure contexts within Ethiopia (and possibly outside the country as well). This regimen would be expected to have a similar efficacy as the standard of care and simplify tasks for health workers and supply chains. A review of costing data across a range of suppliers suggests that the cost of RUTF is 5–20% higher than RUSF (depending on the manufacturer [22, 23]) and thus the marginal cost for treatment, once comprehensive costs of a MAM treatment program are included, may be relatively small. The 2023 WHO guidelines also acknowledge the likely equivalence between RUTF and RUSF in the effectiveness for achieving initial nutritional recovery in the treatment of MAM [5]. This would be one of the first trials to test this hypothesis prospectively.
- MAM experimental group B. Children in this group will receive two sachets of RUTF to consume daily until discharge. This reflects the draft simplified emergency protocol for SAM treatment in Ethiopia. Using this same regimen for MAM in addition to SAM would be a vast simplification for frontline health workers and for the health system in terms of demand planning and purchasing at scale. The cost per day of food would certainly be higher in this regimen, but a faster time to recovery may negate the higher daily cost. The benefits in terms of relapse rates and growth may also be greater with this regimen.

Intervention trials 1 and 2 follow-up visits during treatment

Children will be reassessed at health posts and health centers during follow-up visits every 7 days (SAM trial) or every 14 days (MAM trial) for up to 16 weeks after the time of enrollment, the maximum length of stay prescribed in the Ethiopian national guidelines. At each follow-up visit, a full set of anthropometric measurements will be reassessed, recent infectious symptoms (vomiting, diarrhea, fever, cough) will be queried of the caregiver, and appropriate appetite verified.

Children who appear to be progressing towards recovery will receive additional allotments of their assigned intervention. Children who are not progressing well (e.g., poor weight gain, feeding or health concerns expressed by their caregivers, poor clinical appearance including medical complications, etc.) will be either re-engaged in nutritional and health counseling, including the importance of full adherence to the allocated intervention, or referred to a higher level of care. Clinical assessments for complications will be completed similar to enrollment.

Intervention trials 1 and 2 treatment outcomes

At each follow-up visit, children in the AM trials will receive one of the classifications listed in Table 2. Children will be assigned a final discharge outcome of recovery, defined as having a WLZ at two standard deviations below the median or better *and* MUAC at 125 mm or better *and* no edema, all for two consecutive visits. Children will be considered to have not recovered with any of the following discharge outcomes: deterioration to SAM

(for children in the MAM trial), death, hospitalization, lost to follow-up, and remaining with AM after 16 weeks of treatment.

Intervention trials 1 and 2 sample size

The powered objective for the AM intervention trials will be the rates of anthropometric recovery by 16 weeks (Table 2), comparing each intervention group individually against the standard of care (control) group, based on the hypothesis that the novel interventions are non-inferior.

A 2020 systematic review of treatment outcomes among children treated for SAM as outpatients with the standard dosing of RUTF showed an average recovery rate of 70% [24]. In Ethiopia specifically, a recent study in Gondar found approximately 75% of children with SAM treated as outpatients recovered [25]. Another recent study in Oromia found that children enrolled by MUAC alone had a recovery rate of approximately 85% [26]. Less complete outcome data exists for children with MAM, but an operational study from southern Ethiopia demonstrated a 73% recovery rate for children with MAM treated with RUSF [27]. Based on these accumulated data, we have powered the study based on an anticipated recovery rate of 75% for both the SAM and MAM studies.

The non-inferiority margin that is considered clinically acceptable is a relatively subjective judgment that needs to incorporate local sociocultural preferences and economic considerations. Trial cost and duration limitations must also be considered. To help inform this decision, we conducted interviews with health care workers and community leaders within Ethiopia, as well as discussions with our technical advisory groups. There was consensus that a non-inferiority margin of 5–10% would be considered clinically useful, although there was a notable preference for as small of a margin as would be feasible to provide the most robust data, especially compared to prior trials of modified dosing regimens which have used comparatively larger non-inferiority margins. Given resource constraints, we will thus aim for a non-inferiority margin of 6% for each trial, but may have to compromise to up to 10% if enrollment challenges arise.

The α parameter (Type I error) will be set at 0.05, indicating a 5% risk of identifying a difference between an experimental group and the control group where none really exists. The β value (Type II error) will be set at 0.20, thereby giving a power of 80% to identify a difference between an experimental group and the control arm. Due to possible incorrect enrollments, incomplete or inaccurate data collection, and/or drop-outs, we will aim to enroll an additional 10% in each study arm beyond the sample size target calculation.

Based on these parameters, we determined that the minimum sample size needed for a non-inferiority margin of 6% is 798 children per study arm; the minimum needed for a non-inferiority margin of 8% is 462 children per study arm; for 10%, the minimum needed for 10% would be 305 [28]. Multiplying the largest of these by three study arms per trial gives a targeted sample size for enrollment of 2400 children in each trial. This warrants an enrollment of an average of 140 children per month in each trial. If enrollment is slower than anticipated, requiring an adaptation to the enrollment goal, then 1400 children in each trial will be sought to achieve a non-inferiority margin of 8% in a three-armed trial, or 915 for a 10% non-inferiority margin.

Post-recovery– trial 3

Children from all 3 arms in each of the 2 AM intervention trials who achieve anthropometric recovery within 16 weeks of enrollment will then be offered enrollment into the third of the three integrated trials encompassed by the MODAM study (Fig. 1). This post-recovery trial aims to offer insights into numerous outstanding questions in the AM field, given that high rates of post-recovery relapse and mortality place heavy burdens on operational SAM and MAM treatment programs [16, 29]. High post-SAM relapse is a notable problem in Ethiopia, where such cases make up a high proportion of CMAM enrollments [30–32].

Among issues to be explored include the sustainability of anthropometric recovery following the various novel interventions being tested for SAM and MAM treatment, in addition to an investigation of other post-discharge health outcomes such as the overall growth trajectory over the subsequent six months. A major concern in the domain of modified dosages has been the possibility of decreased linear growth velocities among those children treated with proposed modified dosages which has been observed in some trials [21, 33], but not others [34]. These medium-to-long term outcomes are an important indicator to be considered in the overall quality of CMAM programs [35], especially those using novel dosing strategies [36].

Also to be investigated are whether certain post-recovery monitoring strategies are more effective in the medium-term (6 months) to improve early identification of relapse. The theoretical underpinning for the importance of early identification is that when children are less wasted, they are more likely to have better outcomes when readmitted for AM care, including higher likelihood of recovery, less time and resources needed for recovery, and less long-term growth faltering and morbidity. In addition to more frequent follow-up visits to monitor for relapse, some evidence has suggested that children's caregivers are themselves able to successfully

screen for low MUAC and edema at home, potentially providing earlier identification and diagnosis [37–40]. This is presumed to have the benefit of earlier entry into care, higher likelihood of rapid recovery, and less long-term consequences of AM.

For this third trial, children who recover will thus be enrolled in the post-recovery follow-up trial on the day of recovery from any arm of either of the intervention trials. They will be randomized using similar methods as described above into one of three different study arms and followed for 24 weeks:

- Standard of care (control) group. Children in this group will receive standard nutrition, infant and young child feeding (IYCF), and water, sanitation, and hygiene (WASH) education [20]. Caregivers will be educated to return to the health post or seek care from a HEW if they have a health or nutritional concern regarding their child. A single follow-up visit at 24 weeks after initial AM recovery (i.e., enrollment into the post-recovery trial) will be scheduled to assess anthropometry and nutritional status.
- Experimental group A: Active Follow-up. In addition to the interventions received by the control group, caregivers will be requested to revisit the health post/center for scheduled follow-up visits at 4,12, and 24 weeks after recovery. If a caregiver fails to attend the scheduled health post/center visit, then a HEW or study staff member travel to the child's home to remind them to complete the scheduled follow-up visit the following week or directly collect the data at the home as a last resort. This group is considered to have the most "active" follow-up procedures implemented by healthcare workers and volunteers.
- Experimental group B: Family MUAC Follow-up. In addition to the interventions received by the control group, caregivers will receive training on how to implement home-based MUAC and edema screening, including provision of MUAC tapes [41]. Caregivers will be educated how and when to assess their child's MUAC and edema, and to return for care if their child's MUAC falls into the AM category, their child has bilateral pitting edema, or at any time they are concerned about their child's overall health. These children will also be scheduled for a single follow-up visit at 24 weeks after initial AM recovery.

Whenever a child in the post-recovery trial, regardless of study arm, is brought back to the health post, anthropometry will be assessed by study staff to determine whether or not the child relapsed to AM as well as to record overall growth trajectory. An additional health and demographics questionnaire will be completed. Those identified with AM will then receive treatment per the standard national protocol and continued to be followed for a total of 24 weeks from the time of initial recovery as some may relapse multiple times.

Post-recovery outcomes of interest

To help inform the assessment of the various novel treatment protocols, a number of parameters will be investigated over the 24-week post-recovery follow-up period, including, but not limited to:

- Vital and nutritional status, including rates of relapse to SAM and MAM, hospitalization, loss to follow-up, and mortality.
- Weight gain, as measured in g, g/kg/d, WAZ, and WLZ.
- MUAC gain, as measured in mm, mm/d, and MUAC-for-age Z-score (MUACZ).
- Length gain, as measured in cm, mm/d, and length-for-age z-score (LAZ).
- Rate, severity, and frequency of relapse and mortality.

The relative effectiveness of the three different post-recovery strategies will be investigated by assessing a number of parameters, including, but not limited to, the following:

- Frequency of return visits to the health post.
- History of hospitalizations.
- Frequency of identification of relapse to AM.
- Number of person-months with AM after initial recovery.
- Anthropometry at the time of relapse.
- Anthropometry at 24-week post-recovery.

Statistical analyses

Standard statistical approaches will be used in each of the three trials to analyze each intervention arm independently in comparison to the control arm. Dichotomous variables will be compared using Chi-square testing. Continuous variables will be analyzed using the Student t-test. Rates of recovery and relapse will be evaluated using Kaplan-Meier survival analyses. Regression analyses will be used as appropriate to control for confounding variables. In general, P values less than 0.05 will be considered statistically significant, although this will be reduced in the case of multiple comparisons. 95% confidence intervals will be calculated in addition to P values in order to provide additional detail about error margins in the various calculations. Intention-to-treat analyses will be used throughout, as this will provide the most pragmatic and applicable evidence to inform the real-world scale-up of these interventions.

Data and safety monitoring

A data and safety monitoring board (DSMB) has been established for the two AM intervention trials, given that many children enrolled in the novel intervention arms may receive less specially-formulated foods in

their treatment than in the standard of care models. The DSMB will periodically review and evaluate the accumulated study data for participant safety, study conduct, and progress. Significant adverse events will be reported to the DSMB on an ongoing basis as they occur.

Masked outcome data will be provided to the DSMB after approximately every 600 subjects in each treatment trial have completed their treatment and been classified as either recovered or not. Given the goal of enrolling 2400 children in each trial, this equates to three interim analyses (at 600, 1200, and 1800 outcomes per trial). The O'Brien-Fleming boundary [42] will be used by the DSMB to recommend stopping early any experimental arm in case that arm's recovery rate or mortality rate is significantly worse than the control arm.

DSMB recommendations to stop an experimental arm will be taken up by the MODAM study principal investigators and external technical advisory group for consideration as soon as possible after the recommendation is made. Any children currently enrolled in the trial if and when a decision is made to close an experimental arm will be transferred to standard of care (control arm) and will have their data censored as a non-study enrollment.

Cost effectiveness analysis

A sub-study evaluating cost effectiveness from an institutional perspective will be conducted to help inform total program costs, as well as the proportion of program costs linked to initial treatment vs. treatment of relapse for both the MAM and SAM trials. Cost per child treated, cost per child cured, costs per child who sustains recovery, and costs per child who fails to sustain recovery will be calculated for each study arm.

An activity and ingredients-based approach will be used to estimate total program costs. Cost data will be collected from accountancy records and through key informant interviews. Time allocation interviews will be conducted to determine personnel costs, apportion shared costs to project activities, and to exclude research costs. Program cost data will be adjusted for inflation and capital cost depreciation and assigned to the MAM and SAM studies. To differentiate between initial and relapse treatment costs, a per-visit cost will be calculated. This cost will then be multiplied by the length of stay to determine the average cost per child for initial and relapse treatment episodes. These costs, in turn, will then be multiplied by the number of children treated and relapsed, to estimate the total cost of initial vs. relapse treatment, respectively. Sensitivity analyses will be conducted where necessary to determine how uncertainty in costs and outcomes might affect cost-efficiency and cost-effectiveness estimates.

Discussion

The MODAM study encompasses three distinct, yet integrated, randomized controlled clinical trials evaluating the effectiveness of a number of novel interventions that have the potential to improve CMAM programs for the management of children with AM. The results will add to the context-specific body of knowledge available to guide policymakers in Ethiopia and other countries as they plan how to most efficiently implement and expand their national programs to address AM. Specifically, we anticipate providing new insights into the effectiveness of modified dosages of specially-formulated foods for both SAM and MAM, which are especially important considering the widespread adoption of some novel dosing schedules in many settings without a strong body of evidence.

In addition to the modified dosing strategies proposed, the MODAM trial has at least three significant design strengths that have not often been used in previous modified dosage studies. We believe these strengths may generate robust, compelling evidence that would provide new perspectives for policymakers before implementing modified dosage protocols. First is that we will prospectively provide different treatments and disaggregate results for children with SAM from MAM, as children with SAM have been seen to have less successful outcomes compared to those with MAM with these modified approaches [43]. While treating all children with AM in a unified program with a unified dosing regimen is certainly appealing, this runs the risk of being an *over*simplification due to the higher vulnerability of children with SAM. Second is that the individual participant randomization design, which we anticipate will provide a more robust level of evidence compared to cluster randomization used in prior studies in Ethiopia [44, 45]. In addition to baseline differences among the communities found in different clusters, cluster randomized trials are vulnerable to incorrect analytical methods, insufficient sample size, and improper analysis using the individual rather than the cluster as the unit of analysis [46, 47]. Third is that we will aim for a significantly smaller non-inferiority margin, with an associated larger sample size than most prior modified dosage studies, which we hope will provide more subtle information to stakeholders regarding both primary and secondary outcomes.

Only a small body of evidence exists regarding the experimental dosing regimens being tested for SAM treatment. There remains equipoise in terms of the interventions' effectiveness, given the lower quantity of RUTF that most children will receive and some experience showing higher rates of stunting following discharge; however, the potential for significant cost savings, increased coverage, and simplification for frontline health workers may make these modified dosing schedules a

worthwhile pursuit nonetheless. We hope that the development of cost-efficiency models incorporating the data generated by these trials will help provide policymakers with the information they need to make fully informed decisions as they balance these conflicting imperatives.

The experimental dosing regimens we will investigate for children with MAM have not been previously studied as far as we are aware, and include one experimental arm putting to the test a new WHO recommendation that RUTF may be used instead of RUSF for MAM [5]. While it would seem rather obvious that RUTF would be just as effective as RUSF, given that RUTF has an even better mix of complete protein than RUSF, its lower micronutrient content and less energy per sachet makes its presumed effectiveness less than guaranteed. The third experimental arm for MAM takes quite a different approach from the previously tested regimens of *reduced* dosage, instead opting for an increased dosage using 2 sachets (1000 kcal) of RUTF daily. The hypothesis here is that, by significantly increasing the amount of treatment food consumed on a daily basis, the length of stay might be significantly shortened, thereby saving financial costs overall and also decreasing the morbidity burden these children suffer while acutely malnourished if they are able to recover quickly.

We also aim to provide evidence regarding the effectiveness of the Family MUAC approach for a targeted high-risk population (children who have recently recovered from AM) known to have a significantly elevated risk of development of AM [16, 29]. Given the limited resources available for Family MUAC and the inconsistency of its success in the early identification of children with AM, this targeted approach is an opportunity to focus health worker resources on those for whom the benefit may be highest. The experience these caregivers have with previous successful rehabilitation from AM for their children may also increase their motivation to follow through with the home-based AM screening and to bring their children back to health posts/centers more rapidly, as they have seen the life-saving effects of the AM treatment. As far as we are aware, this is the first prospective study of the Family MUAC focused on this post-AM population and may provide a novel path forward for an operationally practical and cost-effective implementation of Family MUAC programs.

CMAM programs in high-burden settings all over the world struggle from similar challenges related to insufficient coverage for both diagnosis and treatment of AM, unfortunately largely a function of limited financial resources. The MODAM study is designed to provide insights into how to overcome some of these issues in an operational setting by rigorously testing approaches that can help extend available supplies of therapeutic and supplementary foods to treat more children, simplify supply chains, and more readily identify children at high risk for AM at an earlier stage of illness.

An important, and potentially challenging, aspect of the MODAM study is that this will be one of the first CMAM studies to incorporate the new 2023 WHO guideline on AM recovery criteria, requiring *all* three criteria of edema, MUAC, and WLZ to meet recovery thresholds [5]. This new recommendation comes from an appropriate logic, in that this ensures that children who are deemed recovered are not immediately readmitted using a different anthropometric criterion, but does carry the risk of prolonged length of stay and a higher number of children who would be deemed "failures" to recover. Our experience here will thus help to provide some of the first evidence on the effect this new WHO recommendation has in practice.

We have aimed to enroll rather large sample sizes (800 children in each study arm) in order to provide a relatively small non-inferiority margin (6%) for the AM treatment trials. This is ambitious, given the challenging situation in Ethiopia recently in terms of conflict in many areas and the relatively low rates of AM in areas not already being served by other CMAM program evaluations. There is thus the distinct possibility we will not be able to enroll the full sample size desired before trial funding runs out and we may need to accept a smaller sample size or eliminate one experimental arm in a given trial in order to achieve the same non-inferiority margin. It is also possible that recent violent conflicts will spill over into study areas causing potential pauses in research operations. Nevertheless, we aim to anticipate these potential challenges through the study team's close collaborations with the Federal Ministry of Health and UNICEF, to proactively monitor contextual factors and plan accordingly.

At the conclusion of each trial in the MODAM study, results will be disseminated as soon as possible to the Federal Ministry of Health in Ethiopia and surrounding regions through consultative workshops and other platforms, in addition to peer-reviewed publications. We will make every effort to help ministries of health with interpretation and appropriate contextualization and implementation of any significant findings into national protocols that will support maximal coverage of optimal care for acutely malnourished children. After publication, raw de-identified data will be made publicly available and efforts will be made to collaborate with other researchers on additional analyses not yet anticipated.

Abbreviations

AM	Acute malnutrition
CMAM	Community-based management of acute malnutrition
COVID-19	Coronavirus disease 2019
DSMB	Data and safety monitoring board
HEW	Health extension worker
IYCF	Infant and young child feeding

LAZ	Length-for-age Z-score
MAM	Moderate acute malnutrition
MODAM	Modified Dosages for Acute Malnutrition study
MUAC	Mid-upper arm circumference
MUACZ	Mid-upper arm circumference-for-age Z-score
RUSF	Ready-to-use supplementary food
RUTF	Ready-to-use therapeutic food
SAM	Severe acute malnutrition
WASH	Water, sanitation, and hygiene
WHO	World Health Organization
WLZ	Weight-for-length Z-score

Acknowledgements
We are grateful to the MODAM Study Technical Advisory Group (Tefera Belachew, James Berkley, Mufaro Chiriga, Wondayferam Gemeda, Aweke Kebede, Carol Levin, Mark Manary, Susan Shepard, Parminder Suchdev), the Data and Safety Monitoring Board (Adebabay Amare, Tigist Bacha, Seifu Hagos, Yehenew Kifle, Dessalegn Tamiru), collaborators on the cost-efficiency model (Sarah King, Navideh Noori, Laura Skrip), Action Against Hunger Ethiopia Staff (Beza Abebe, Muluneh Girma, Ria Jusufbegovic, and Mhiret Teshome), and UNICEF Ethiopia staff (Rashid Abdulai, Anne-Sophie Donze, Ramadhani Noor).

Author contributions
I.T., Y.B., H.D., B.S.A., M.W., G.G., L.A.L., B.C., S.C., M.W.S., M.T., and H.C.S. contributed to the design and development of the trials described here. I.T. wrote the first version of the manuscript. H.C.S. secured trial funding and is responsible for overall management of trial execution. All authors read and approved the final manuscript.

Funding
The MODAM study is funded by a consortium of funders through the Power of Nutrition, including the Eleanor Crook Foundation, Rotary International, and the END fund, as well as direct funding from Action Against Hunger USA.

Data availability
No datasets were generated or analysed during the current study.

Declarations

Ethics approval and consent to participate
Ethical approval was provided by the Ethiopian Public Health Institute (protocol EPHI-IRB-498-2023) and the University of Washington.

Consent for publication
Not applicable.

Competing interests
The authors declare no competing interests.

Author details
[1]Departments of Pediatrics, Global Health, and Epidemiology, University of Washington, Seattle, WA, USA
[2]Ethiopian Public Health Institute, Addis Ababa, Ethiopia
[3]Federal Ministry of Health, Addis Ababa, Ethiopia
[4]Action Against Hunger USA, New York, NY, USA
[5]Action Against Hunger USA, Addis Ababa, Ethiopia
[6]Action Against Hunger UK, London, UK
[7]United Nations Children's Fund, Addis Ababa, Ethiopia

Received: 2 August 2024 / Accepted: 27 March 2025
Published online: 08 April 2025

References

1. Bhutta ZA, Berkley JA, Bandsma RHJ, et al. Severe childhood malnutrition. Nat Rev Dis Primers. 2017;3:17067.
2. United Nations Children's Fund, World Health Organization, International Bank for Reconstruction and Development, World Bank Group. Levels and trends in child malnutrition: UNICEF / WHO / World Bank Group joint child malnutrition estimates: Key findings of the 2023 edition. 2023.
3. de Onis M, Garza C, Onyango AW, Martorell R. WHO child growth standards. Acta Paediatr. 2006;450:S1–101.
4. World Health Organization. WHO child growth standards and the identification of severe acute malnutrition in infants and children. 2009.
5. World Health Organization. WHO guideline on the prevention and management of wasting and nutritional oedema (acute malnutrition) in infants and children under 5 years. 2023.
6. USAID UNICEF. IRC, Action Against Hunger, Save the Children. Scaling up coverage, quality and sustainability of early detection and treatment of severe wasting programming for children. 2023.
7. Emergency Nutrition Network. Simplified approaches to the treatment of wasting: Technical brief. 2020.
8. United Nations Children's Fund. Treatment of wasting using simplified approaches: A rapid evidence review. 2020.
9. United Nations Children's Fund. Simplified Approaches Working Group. Using simplified approaches in exceptional circumstances. 2022.
10. Wrabel M, Stokes-Walters R, King S, Funnell G, Stobaugh H. Programmatic adaptations to acute malnutrition screening and treatment during the COVID-19 pandemic. Matern Child Nutr. 2022;18(4):e13406.
11. López-Ejeda N, Charle-Cuellar P, Samake S, et al. Effectiveness of decentralizing outpatient acute malnutrition treatment with community health workers and a simplified combined protocol: A cluster randomized controlled trial in emergency settings of Mali. Front Public Health. 2024;12:1283148.
12. Tesfaye GA, Wana EW, Workie MG. Critically high burden of acute malnutrition and its associated factors among children under 5 years in the rural settings of Southern Ethiopia. Food Nutr Bull. 2024;45(1):38–46.
13. Alyi M, Roba KT, Ketema I, et al. Relapse of acute malnutrition and associated factors after discharge from nutrition stabilization centers among children in Eastern Ethiopia. Front Nutr. 2023;10:1095523.
14. Teshale EB, Nigatu YD, Delbiso TD. Relapse of severe acute malnutrition among children discharged from outpatient therapeutic program in Western Ethiopia. BMC Pediatr. 2023;23(1):441.
15. Yitayew YA, Yalew ZM, Nebiyu S, Jember DA. Acute malnutrition relapse and associated factors among 6–59 months old children treated in the community-based management of acute malnutrition in Dessie, Kombolcha, and Haik towns, Northeast Ethiopia. Front Public Health. 2023;11:1273594.
16. Stobaugh HC, Mayberry A, McGrath M, et al. Relapse after severe acute malnutrition: A systematic literature review and secondary data analysis. Matern Child Nutr. 2019;15(2):e12702.
17. Schaefer R, Mayberry A, Briend A, et al. Relapse and regression to severe wasting in children under 5 years: A theoretical framework. Matern Child Nutr. 2021;17(2):e13107.
18. Cashin K, Oot L. Guide to anthropometry: A practical tool for program planners, managers, and implementers. 2018.
19. Trehan I, Manary MJ. Management of severe acute malnutrition in low-income and middle-income countries. Arch Dis Child. 2015;100(3):283–7.
20. Government of Ethiopia. National guideline for the management of acute malnutrition in Ethiopia. 2019.
21. Bailey J, Opondo C, Lelijveld N, et al. A simplified, combined protocol versus standard treatment for acute malnutrition in children 6–59 months (ComPAS trial): A cluster-randomized controlled non-inferiority trial in Kenya and South Sudan. PLoS Med. 2020;17(7):e1003192.
22. United Nations Children's Fund. Therapeutic spread, sachet 92 g/car-150. 2024: https://supply.unicef.org/s0000240.html. Accessed on July 23, 2024.
23. United Nations Children's Fund. Supplementary spread, sachet 100 g/car-150. 2024: https://supply.unicef.org/s0000248.html. Accessed on July 23, 2024.
24. Bitew ZW, Alemu A, Worku T. Treatment outcomes of severe acute malnutrition and predictors of recovery in under-five children treated within outpatient therapeutic programs in Ethiopia: A systematic review and meta-analysis. BMC Pediatr. 2020;20(1):335.
25. Debie A, Kassie GM, Tsehay CT et al. Recovery rate of severe acute malnourished children aged 6–59 months enrolled in outpatient therapeutic program at health posts of central Gondar zone, Ethiopia. Nutr Health. 2022:2601060221137102.
26. Lambebo A, Tamiru D, Belachew T. Utilization of mid-upper arm circumference as discharge tool for children in outpatient therapeutic program, Ethiopia. J Nutr Sci. 2022;11:e101.
27. Karakochuk C, van den Briel T, Stephens D, Zlotkin S. Treatment of moderate acute malnutrition with ready-to-use supplementary food results in higher overall recovery rates compared with a corn-soya blend in children in Southern Ethiopia: an operations research trial. Am J Clin Nutr. 2012;96(4):911–6.

28. Faul F, Erdfelder E, Lang AG, Buchner A. G*Power 3: A flexible statistical power analysis program for the social, behavioral, and biomedical sciences. Behav Res Methods. 2007;39(2):175–91.
29. King S, Marshak A, Mello-Guyett L, et al. Rates and risks factors for relapse among children recovered from severe acute malnutrition: A multi-country, prospective cohort study in Mali, South Sudan, and Somalia. SSRN: Preprints Lancet. 2023. https://doi.org/10.2139/ssrn.4664539.
30. Lambebo A, Tamiru D, Belachew T. Time to relapse of severe acute malnutrition and risk factors among under-five children treated in the health posts of Hadiya zone, Southern Ethiopia. J Nutr Sci. 2021;10:e105.
31. Lambebo A, Temiru D, Belachew T. Frequency of relapse for severe acute malnutrition and associated factors among under five children admitted to health facilities in Hadiya zone, South Ethiopia. PLoS ONE. 2021;16(3):e0249232.
32. Girma T, James PT, Abdissa A, et al. Nutrition status and morbidity of Ethiopian children after recovery from severe acute malnutrition: prospective matched cohort study. PLoS ONE. 2022;17(3):e0264719.
33. Kangas ST, Salpeteur C, Nikiema V, et al. Impact of reduced dose of ready-to-use therapeutic foods in children with uncomplicated severe acute malnutrition: A randomised non-inferiority trial in Burkina Faso. PLoS Med. 2019;16(8):e1002887.
34. Cazes C, Phelan K, Hubert V, et al. Simplifying and optimising the management of uncomplicated acute malnutrition in children aged 6–59 months in the Democratic Republic of the congo (OptiMA-DRC): A non-inferiority, randomised controlled trial. Lancet Glob Health. 2022;10(4):e510–20.
35. O'Sullivan NP, Lelijveld N, Rutishauser-Perera A, Kerac M, James P. Follow-up between 6 and 24 months after discharge from treatment for severe acute malnutrition in children aged 6–59 months: A systematic review. PLoS ONE. 2018;13(8):e0202053.
36. Kangas ST, Coulibaly IN, Tausanovitch Z et al. Post-recovery relapse of children treated with a simplified, combined nutrition treatment protocol in Mali: A prospective cohort study. Nutrients 2023; 15(11).
37. Blackwell N, Myatt M, Allafort-Duverger T, et al. Mothers understand and can do it (MUAC): A comparison of mothers and community health workers determining mid-upper arm circumference in 103 children aged from 6 months to 5 years. Arch Public Health. 2015;73(1):26.
38. Alé FG, Phelan KP, Issa H, et al. Mothers screening for malnutrition by mid-upper arm circumference is non-inferior to community health workers: results from a large-scale pragmatic trial in rural Niger. Arch Public Health. 2016;74(1):38.
39. Bliss J, Lelijveld N, Briend A, et al. Use of mid-upper arm circumference by novel community platforms to detect, diagnose, and treat severe acute malnutrition in children: A systematic review. Glob Health Sci Pract. 2018;6(3):552–64.
40. Tickell KD, Achieng C, Masheti M, et al. Family MUAC supported by a two-way SMS platform for identifying children with wasting: the Mama Aweza randomised controlled trial. EClinicalMedicine. 2023;64:102218.
41. ALIMA, Mother, -MUAC. Teaching mothers to screen for malnutrition - guidelines for training of trainers. 2016.
42. Dodd LE, Proschan MA. Data and safety monitoring board monitoring of clinical trials for early efficacy. NEJM Evid. 2022;1(3):EVIDctw2100025.
43. Heymsfield G, Tausanovitch Z, Christian LG et al. Effectiveness of acute malnutrition treatment with a simplified, combined protocol in central African Republic: an observational cohort study. Matern Child Nutr. 2024:e13691.
44. Maru Y, Tamiru D, Baye K, et al. Comparing time to recovery in wasting treatment: simplified approach vs. Standard protocol among children aged 6–59 months in Ethiopia-a cluster-randomized, controlled, non-inferiority trial. Front Pediatr. 2024;12:1337370.
45. Maru Y, Tamiru D, Baye K et al. Effect of a simplified approach on recovery of children 6–59 months with wasting in Ethiopia: A noninferiority, cluster randomized controlled trial. Matern Child Nutr. 2024:e13670.
46. Brown AW, Li P, Bohan Brown MM, et al. Best (but oft-forgotten) practices: designing, analyzing, and reporting cluster randomized controlled trials. Am J Clin Nutr. 2015;102(2):241–8.
47. Turner EL, Platt AC, Gallis JA, et al. Completeness of reporting and risks of overstating impact in cluster randomised trials: A systematic review. Lancet Glob Health. 2021;9(8):e1163–8.

Publisher's note

Springer Nature remains neutral with regard to jurisdictional claims in published maps and institutional affiliations.
