# Supplementary material for: Comparative effectiveness of lipid-nutrient spread supplementation regimens for moderate acute malnutrition in Ethiopia: The MODAM-MAM individually randomized controlled trial
Source: PLoS Med. 2026 Aug 11;23(8):e1005199. doi: 10.1371/journal.pmed.1005199 (PMC13475968; doi:10.1371/journal.pmed.1005199)
Supplement: Supplementary file 2 [file pmed.1005199.s001.docx]

**S1 Table: Additional enrollment characteristics of children included in the intention-to-treat analysis, by treatment group**

|  | **1-RUSF** | **1-RUTF** | **2-RUTF** |
| --- | --- | --- | --- |
|  | n=801 | n=793 | n=818 |
| Admitted with MUAC 11.5-12.4 cm | 499 [62.3%] | 478 [60.3%] | 509 [62.2%] |
| MUAC (cm) if admitted with low MUAC | 12.1 [11.9 to 12.3] | 12.1 [12.0 to 12.3] | 12.2 [12.0 to 12.3] |
| Admitted with WHZ -2 to -3 | 531 [66.3%] | 516 [65.1%] | 511 [62.5%] |
| WHZ if admitted with WHZ -2 to -3 | -2.40 [-2.62 to -2.19] | -2.38 [-2.60 to -2.22] | -2.37 [-2.62 to -2.18] |
| Admitted with WHZ -2 to -3 *and* MUAC 11.5-12.4 cm | 251 [31.3%] | 232 [29.3%] | 234 [28.6%] |
| MUAC (cm) if admitted with low MUAC *and* WHZ | 12.0 [11.8 to 12.3] | 12.2 [12.0 to 12.3] | 12.2 [11.9 to 12.3] |
| WHZ if admitted with low MUAC *and* WHZ | -2.44 [-2.71 to -2.21] | -2.44 [-2.66 to -2.24] | -2.44 [-2.70 to -2.24] |
| Admitted with WAZ <-3 | 266 [33.2%] | 221 [27.9%] | 240 [28.3%] |
| WAZ if admitted with low WAZ | -3.44 [-3.76 to -3.18] | -3.45 [-3.82 to -3.21] | -3.45 [-3.83 to -3.17] |
| Admitted with HAZ <-2 | 409 [51.1%] | 369 [46.5%] | 385 [47.1%] |
| HAZ if admitted with low HAZ | -2.99 [-3.75 to -2.56] | -3.01 [-3.73 to -2.44] | -3.08 [-3.82 to -2.48] |

Data are n (%) or median (IQR), as appropriate. Indented rows show the distribution of anthropometric measures among children within each subgroup at admission.

MUAC=mid-upper arm circumference; WHZ=weight-for-height z-score; HAZ=height-for-age z-score; WAZ=weight-for-age z-score; RUSF=ready to use supplementary food; RUTF=ready to use therapeutic food.
