# Supplementary material for: Comparative effectiveness of lipid-nutrient spread supplementation regimens for moderate acute malnutrition in Ethiopia: The MODAM-MAM individually randomized controlled trial
Source: PLoS Med. 2026 Aug 11;23(8):e1005199. doi: 10.1371/journal.pmed.1005199 (PMC13475968; doi:10.1371/journal.pmed.1005199)
Supplement: Supplementary file 3 [file pmed.1005199.s002.docx]

|  | **Gode** | **Teltele** |  |  |
| --- | --- | --- | --- | --- |
| **Characteristic** | n=1,195 | n=1,217 | Difference [95% CI] | |
| **Child Characteristics** |  |  |  | |
| Female | 599 [50.1%] | 616 [50.6%] | 0.50 [-3.50 to 4.50] | |
| Age (months) | 23.2 [12.3 to 36.0] | 18.1 [11.8 to 35.3] | -1.90 [-2.80 to -1.00] | |
| < 24 months | 640 [53.6%] | 692 [56.9%] | 3.30 [-0.70 to 7.30] | |
| MUAC (cm) | 12.5 [12.1 to 13.2] | 12.3 [12.0 to 12.5] | -0.30 [-0.30 to -0.20] | |
| Height (cm) | 78.0 [70.5 to 90.1] | 75.4 [68.6 to 85.2] | -2.60 [-3.50 to -1.70] | |
| Weight (kg) | 8.2 [7.0 to 10.5] | 7.9 [6.9 to 9.7] | -0.30 [-0.40 to -0.10] | |
| WHZ | -2.28 [-2.53 to -2.03] | -2.04 [-2.40 to -1.51] | 0.26 [0.22 to 0.31] | |
| HAZ | -1.88 [-2.97 to -0.68] | -1.99 [-2.98 to -0.90] | -0.13 [-0.27 to -0.00] | |
| WAZ | -2.54 [-3.18 to -1.92] | -2.40 [-3.06 to -1.81] | 0.12 [0.04 to 0.19] | |
| **Admission Characteristics** |  |  |  | |
| Admitted with MUAC 11.5-12.4 cm | 573 [48.0%] | 913 [75.0%] | 27.10 [23.30 to 30.80] | |
| MUAC if admitted with low MUAC | 12.1 [11.9 to 12.3] | 12.2 [12.0 to 12.3] | -0.00 [-0.00 to -0.00] | |
| Admitted with WHZ -2 to -3 | 922 [77.2%] | 636 [52.3%] | -24.90 [-28.60 to -21.20] | |
| WHZ if admitted with WHZ -2 to -3 | -2.38 [-2.61 to -2.21] | -2.38 [2.62 to -2.18] | 0.01 [-0.01 to 0.04] | |
| Admitted with WHZ -2 to -3 *and* MUAC 11.5-12.4cm | 345 [28.9%] | 372 [30.6%] | 1.70 [-2.00 to 5.30] | |
| MUAC if admitted with low MUAC *and* WHZ | 12.1 [11.8 to 12.3] | 12.2 [12.0 to 12.3] | -0.00 [-0.00 to 0.10] | |
| WHZ if admitted with low MUAC *and* WHZ | -2.47 [-2.73 to -2.28] | -2.41 [-2.67 to -2.19] | 0.06 [0.01 to 0.10] | |
| Admitted with WAZ <-3 | 397 [33.2%] | 330 [27.1%] | -6.10 [-9.80 to -2.50] | |
| WAZ if admitted with low WAZ | -3.45 [-3.80 to -3.18] | -3.44 [-3.80 to -3.20] | 0.01 [-0.05 to 0.06] | |
| Admitted with HAZ <-2 | 560 [46.9%] | 603 [49.6%] | 2.70 [-1.30 to 6.70] | |
| HAZ if admitted with low HAZ | -3.02 [-3.78 to -2.50] | -2.99 [-3.72 to -2.48] | 0.04 [-0.05 to 0.13] | |
| **Health** |  |  |  | |
| Diarrhea in the previous 7 days | 274 [23.0%] | 309 [25.5%] | 2.50 [-1.00 to 5.90] | |
| Fever in the previous 7 days | 375 [31.5%] | 208 [17.2%] | -14.30 [-17.70 to -11.00] | |
| Cough in the previous 7 days | 407 [34.2%] | 282 [23.3%] | -11.00 [-14.60 to -7.40] | |
| Currently breastfed | 453 [37.9%] | 691 [56.8%] | 18.80 [14.80 to 22.70] | |
| **Caregiver Characteristics** |  |  |  | |
| Primary caregiver is mother | 1,164 [97.4%] | 1,172 [96.3%] | -1.20 [-3.00 to -0.40] | |
| Mother has no formal education | 952 [79.7%] | 928 [76.3%] | -3.68 [-7.00 to -3.80] | |
| **Household Characteristics** |  |  |  | |
| Household currently displaced | 49 [4.1%] | 35 [2.9%] | -1.20 [-2.70 to 0.20] | |
| Head of household is female | 101 [8.5%] | 195 [16.0%] | 7.50 [4.90 to 10.10] | |
| Number of children under 10 years old living at home | 4 [3 to 5] | 2 [2 to 3] | -2.00 [-2.00 to -2.00] | |
| Child is a twin | 30 [2.5%] | 61 [5.0%] | 2.50 [1.00 to 4.00] | |
| Primary livelihood of household |  |  |  | |
| Agriculture | 204 [17.1%] | 789 [64.8%] | 47.70 [44.20 to 51.10] | |
| Rearing livestock | 45 [3.8%] | 84 [6.9%] | 2.10 [1.30 to 4.90] | |
| Mixed (livestock and agriculture) | 24 [2.0%] | 121 [9.9%] | 7.90 [6.10 to 9.80] | |
| Casual labor | 621 [52.0%] | 145 [11.9%] | -40.60 [-44.00 to -37.20] | |
| Salaried work | 118 [9.9%] | 9 [0.7%] | -9.20 [-11.00 to -7.50] | |
| Other | 170 [14.2%] | 67 [5.5%] | -8.90 [-11.20 to -6.50] | |

**S2 Table: Enrollment characteristics of children included in the intention-to-treat analysis, by study site**

Data are n [%] or median [IQR], as appropriate. Characteristics were measured at enrollment and are presented by study site. Differences between sites were estimated using the Hodges-Lehmann method, which calculates the median of all pairwise differences between groups; values reflect distributional shifts, rather than simple differences in medians.

MUAC = mid-upper arm circumference; WHZ = weight-for-height z-score; HAZ = height-for-age z-score; WAZ = weight-for-age z-score.
