# Supplementary material for: Comparative effectiveness of lipid-nutrient spread supplementation regimens for moderate acute malnutrition in Ethiopia: The MODAM-MAM individually randomized controlled trial
Source: PLoS Med. 2026 Aug 11;23(8):e1005199. doi: 10.1371/journal.pmed.1005199 (PMC13475968; doi:10.1371/journal.pmed.1005199)
Supplement: Supplementary file 4 [file pmed.1005199.s003.docx]

**S3 Table: Enrollment characteristics of children included in the per-protocol population, by treatment group**

|  | **1-RUSF** | **1-RUTF** | **2-RUTF** |
| --- | --- | --- | --- |
| **Characteristic** | n=753 | n=748 | n=777 |
| **Child Characteristics** |  |  |  |
| Female | 375 [49.8%] | 384 [51.3%] | 400 [51.5%] |
| Age (months) | 20.7 [12.0 to 35.6] | 23.9 [12.1 to 36.0] | 19.5 [12.0 to 35.4] |
| < 24 months | 421 [55.9%] | 380 [50.8%] | 452 [58.2%] |
| MUAC (cm) | 12.3 [12.0 to 12.8] | 12.4 [12.1 to 12.9] | 12.3 [12.1 to 12.8] |
| Height (cm) | 76.3 [68.9 to 87.3] | 78.0 [70.6 to 88.8] | 75.6 [69.3 to 88.4] |
| Weight (kg) | 7.9 [6.8 to 9.9] | 8.3 [7.1 to 10.3] | 7.9 [6.9 to 10.2] |
| WHZ | -2.19 [-2.49 to -1.76] | -2.21 [-2.47 to -1.84] | -2.15 [-2.45 to -1.72] |
| HAZ | -2.06 [-3.00 to -1.01] | -1.85 [-2.91 to -0.69] | -1.89 [-2.94 to -0.78] |
| WAZ | -2.56 [-3.18 to -1.99] | -2.44 [-3.08 to -1.79] | -2.45 [-3.08 to -1.83] |
| **Admission Characteristics** |  |  |  |
| Admitted with MUAC 11.5-12.4 cm | 468 [62.2%] | 451 [60.3%] | 486 [62.6%] |
| MUAC if admitted with low MUAC | 12.1 [11.9 to 12.3] | 12.1 [12.0 to 12.3] | 12.2 [12.0 to 12.3] |
| Admitted with WHZ -2 to -3 | 495 [65.7%] | 487 [65.1%] | 482 [62.0%] |
| WHZ if admitted with WHZ -2 to -3 | -2.40 [-2.62 to -2.19] | -2.38 [-2.60 to -2.22] | -2.37 [-2.62 to -2.19] |
| Admitted with WHZ -2 to -3 *and* MUAC 11.5-12.4cm | 231 [30.7%] | 220 [29.4%] | 223 [28.7%] |
| MUAC if admitted with low MUAC *and* WHZ | 12.1 [11.8 to 12.3] | 12.2 [12.0 to 12.3] | 12.2 [11.9 to 12.3] |
| WHZ if admitted with low MUAC *and* WHZ | -2.43 [-2.71 to -2.20] | -2.44 [-2.67 to -2.25] | -2.44 [-2.69 to -2.23] |
| Admitted with WAZ <-3 | 250 [33.2%] | 210 [28.1%] | 223 [28.7%] |
| WAZ if admitted with low WAZ | -3.45 [-3.75 to -3.18] | -3.46 [-3.84 to -3.22] | -3.44 [-3.81 to -3.18] |
| Admitted with HAZ <-2 | 388 [51.5%] | 349 [46.7%] | 362 [46.6%] |
| HAZ if admitted with low HAZ | -2.98 [-3.74 to -2.53] | -3.01 [-3.76 to -2.45] | -3.08 [-3.81 to -2.48] |
| **Health** |  |  |  |
| Diarrhea in the previous 7 days | 187 [24.9%] | 177 [23.8%] | 192 [24.8%] |
| Fever in the previous 7 days | 216 [28.8%] | 162 [21.8%] | 172 [22.2%] |
| Cough in the previous 7 days | 230 [30.7%] | 199 [26.8%] | 223 [28.7%] |
| Currently breastfed | 385 [52.3%] | 346 [47.0%] | 356 [46.7%] |
| **Caregiver Characteristics** |  |  |  |
| Primary caregiver is mother | 728 [96.9%] | 726 [97.2%] | 750 [97.0%] |
| Mother has no formal education | 573 [76.4%] | 589 [78.7%] | 608 [78.4%] |
| **Household Characteristics** |  |  |  |
| Household currently displaced | 23 [3.1%] | 31 [4.2%] | 19 [2.5%] |
| Head of household is female | 83 [11.0%] | 90 [12.1%] | 99 [12.8%] |
| Number of children under 10 years old living at home | 3 [2 to 4] | 3 [2 to 4] | 3 [2 to 5] |
| Child is a twin | 36 [4.8%] | 23 [3.1%] | 29 [3.8%] |
| Primary livelihood of household |  |  |  |
| Agriculture | 329 [43.9%] | 295 [39.8%] | 327 [42.4%] |
| Rearing livestock | 34 [4.5%] | 44 [5.9%] | 42 [5.4%] |
| Mixed (livestock and agriculture) | 38 [5.1%] | 43 [5.8%] | 60 [7.8%] |
| Casual labor | 248 [33.1%] | 237 [31.9%] | 233 [30.2%] |
| Salaried work | 32 [4.3%] | 47 [6.3%] | 39 [5.1%] |
| Other | 68 [9.1%] | 76 [10.2%] | 71 [9.2%] |
| Enrolled in Gode District, Somali Region | 375 [49.8%] | 361 [48.3%] | 373 [48.0%] |
| Enrolled in Teltele District, Oromia Region | 378 [50.2%] | 387 [51.7%] | 404 [52.0%] |

Data are n [%] or median [IQR], as appropriate. Characteristics were measured at enrollment prior to initiation of treatment.

MUAC = mid-upper arm circumference; WHZ = weight-for-height z-score; HAZ = height-for-age z-score; WAZ = weight-for-age z-score; RUSF = ready-to-use supplementary food; RUTF = ready-to-use therapeutic food.
