# Supplementary material for: Comparative effectiveness of lipid-nutrient spread supplementation regimens for moderate acute malnutrition in Ethiopia: The MODAM-MAM individually randomized controlled trial
Source: PLoS Med. 2026 Aug 11;23(8):e1005199. doi: 10.1371/journal.pmed.1005199 (PMC13475968; doi:10.1371/journal.pmed.1005199)
Supplement: Supplementary file 5 [file pmed.1005199.s004.docx]

**S4 Table: Regression-adjusted primary outcomes among children treated for moderate acute malnutrition, by treatment group in intention-to-treat analysis**

|  | **1-RUSF** | **1-RUTF** | **2-RUTF** | **1-RUSF vs. 1-RUTF** | **1-RUSF vs. 2-RUTF** |
| --- | --- | --- | --- | --- | --- |
|  |  |  |  | Difference | Difference |
| Recovered | 86.0% [83.51 to 88.44] | 86.1% [83.59 to 88.51] | 88.9% [86.76 to 91.13] | 0.08 [-3.44 to 3.60] | 3.08 [-0.25 to 6.42] |
| Not recovered | 14.0% [11.56 to 16.49] | 14.0% [11.49 to 16.41] | 11.1% [8.87 to 13.24] | -0.08 [-3.60 to 3.44] | -3.08 [-6.42 to 0.25] |
| Deteriorated to SAM | 1.1% [0.37 to 1.73] | 0.9% [0.28 to 1.47] | 0.6% [0.14 to 1.08] | -0.32 [-1.67 to 1.03] | -0.83 [-2.04 to 0.38] |
| Referred to hospital | 0.2% [-0.03 to 0.35] | 0.5% [0.04 to 0.91] | 0.6% [0.08 to 1.04] | 1.11 [0.04 to 2.19] | 1.28 [0.31 to 2.46] |
| Remained with MAM | 6.0% [4.36 to 7.71] | 5.5% [3.89 to 7.07] | 3.7% [2.46 to 5.01] | -0.66 [-3.19 to 1.87] | -2.77 [-5.13 to -0.42] |
| Defaulted | 4.2% [2.77 to 5.55] | 4.0% [2.64 to 5.38] | 3.2% [2.03 to 4.44] | -0.17 [-2.28 to 1.94] | -1.04 [-3.03 to 0.94] |
| Died | 0.0% [-0.02 to 0.04] | 0.0% [-0.03 to 0.05] | 0.0% [-0.04 to 0.08] | 0.01 [-0.34 to 0.37] | 0.14 [-0.29 to 0.57] |
| Length of stay (d) | 63.31 [61.23 to 65.39] | 62.31 [60.25 to 64.38] | 59.94 [57.93 to 61.94] | -1.00 [-3.93 to 1.93] | -3.37 [-6.26 to -0.48] |
| Total sachets provided | 72.2 [70.1 to 74.3] | 70.7 [68.7 to 72.7] | 139.5 [135.5 to 143.5] | -1.5 [-4.3 to 1.4] | 67.3 [62.8 to 71.8] |

Values are adjusted percentages or means with 95% confidence intervals derived from regression models. Binary outcomes were modelled using logistic regression and continuous outcomes using Poisson or linear models, adjusting for child age, sex, anthropometry, breastfeeding status, diarrhea, children in the household, and study location.

All values are model-based estimates, and in some cases, the 95% confidence intervals may include values outside the plausible range (e.g., negative percentages) due to statistical uncertainty at very low outcome probabilities. Differences represent adjusted contrasts between study arms and are defined as experimental arm – control arm (1-RUSF).

RUTF = ready-to-use therapeutic food; RUSF = ready-to-use supplementary food; SAM = severe acute malnutrition; MAM = moderate acute malnutrition.
