# Supplementary material for: Comparative effectiveness of lipid-nutrient spread supplementation regimens for moderate acute malnutrition in Ethiopia: The MODAM-MAM individually randomized controlled trial
Source: PLoS Med. 2026 Aug 11;23(8):e1005199. doi: 10.1371/journal.pmed.1005199 (PMC13475968; doi:10.1371/journal.pmed.1005199)
Supplement: Supplementary file 6 [file pmed.1005199.s005.docx]

**S5 Table: Adjusted odds ratios for recovery among all children treated for moderate acute malnutrition (n=2,338 observations)**

| Intervention Comparison | Odds Ratio | 95% CI | p-value |
| --- | --- | --- | --- |
| 1-RUTF*^a^* | 1.007 | 0.76 to 1.34 | 0.964 |
| 2-RUTF*^a^* | 1.313 | 0.98 to 1.76 | 0.071 |

Values are adjusted odds ratios with 95% confidence intervals derived from logistic regression models. Models were adjusted for prespecified enrollment covariates, including age, sex, district, baseline anthropometry (MUAC, WHZ, WAZ), breastfeeding status, recent diarrhea, and number of children under age 10 in the household. Only intervention effect estimates are presented, consistent with recommendations for reporting adjusted analyses in randomized trials.

*^a^* Reference group: 1-RUSF
