# Supplementary material for: Comparative effectiveness of lipid-nutrient spread supplementation regimens for moderate acute malnutrition in Ethiopia: The MODAM-MAM individually randomized controlled trial
Source: PLoS Med. 2026 Aug 11;23(8):e1005199. doi: 10.1371/journal.pmed.1005199 (PMC13475968; doi:10.1371/journal.pmed.1005199)
Supplement: Supplementary file 7 [file pmed.1005199.s006.docx]

**S6 Table: Regression-adjusted growth outcomes among children treated for moderate acute malnutrition by treatment group in intention-to-treat analysis**

|  | **1-RUSF** | **1-RUTF** | **2-RUTF** | **1-RUSF vs. 1-RUTF** | **1-RUSF vs. 2-RUTF** |
| --- | --- | --- | --- | --- | --- |
|  |  |  |  | Adjusted Difference | Adjusted Difference |
| **Weight** |  |  |  |  |  |
| Change in weight from admission to discharge (kg) | 0.72 [0.69 to 0.75] | 0.67 [0.64 to 0.71] | 0.70 [0.67 to 0.74] | -0.04 [-0.09 to 0.00] | -0.01 [-0.06 to 0.03] |
| Rate of weight gain (g/kg/d) | 1.61 [1.51 to 1.71] | 1.53 [1.43 to 1.62] | 1.69 [1.59 to 1.78] | -0.08 [-0.22 to 0.05] | 0.08 [-0.06 to 0.22] |
| Rate of weight gain in the first 2  weeks (g/kg/d) | 1.78 [1.60 to 1.97] | 1.75 [1.56 to 1.94] | 2.03 [1.84 to 2.21] | -0.03 [-0.30 to 0.23] | 0.24 [-0.02 to 0.55] |
| Rate of weight gain in the first 4  weeks (g/kg/d) | 1.57 [1.45 to 1.69] | 1.58 [1.46 to 1.70] | 1.64 [1.52 to 1.76] | 0.00 [-0.16 to 0.17] | 0.07 [-0.10 to 0.23] |
| **MUAC** |  |  |  |  |  |
| Change in MUAC from admission to discharge (cm) | 0.61 [0.57 to 0.64] | 0.61 [0.57 to 0.64] | 0.65 [0.62 to 0.68] | -0.00 [-0.05 to 0.04] | 0.04 [-0.00 to 0.09] |
| Rate of MUAC gain (mm/d) | 0.12 [0.11 to 0.13] | 0.12 [0.11 to 0.13] | 0.13 [0.12 to 0.14] | -0.00 [-0.01 to 0.01] | 0.01 [0.00 to 0.02] |
| MUAC at discharge (cm) | 13.10 [13.07 to 13.13] | 13.10 [13.07 to 13.13] | 13.15 [13.11 to 13.18] | -0.00 [-0.05 to 0.04] | 0.04 [-0.00 to 0.09] |
| **Length** |  |  |  |  |  |
| Change in length from admission to discharge (cm) | 0.80 [0.74 to 0.86] | 0.83 [0.77 to 0.89] | 0.79 [0.73 to 0.84] | 0.04 [-0.05 to 0.12] | -0.01 [-0.09 to 0.07] |
| Rate of length gain (mm/day) | 0.12 [0.12 to 0.13] | 0.13 [0.12 to 0.14] | 0.13 [0.12 to 0.13] | 0.00 [-0.01 to 0.02] | 0.00 [-0.01 to 0.01] |
| **WHZ** |  |  |  |  |  |
| Change in WHZ from admission to discharge | 0.77 [0.72 to 0.81] | 0.70 [0.65 to 0.74] | 0.76 [0.72 to 0.81] | -0.07 [-0.13 to -0.00] | -0.01 [-0.07 to 0.06] |
| WHZ at discharge | -1.30 [-1.34 to -1.25] | -1.36 [-1.41 to -1.32] | -1.30 [-135 to -1.26] | -0.07 [-0.13 to -0.00] | -0.01 [-0.07 to 0.06] |
| **HAZ** |  |  |  |  |  |
| Change in HAZ from admission to discharge | -0.33 [-0.26 to -0.31] | -0.31 [-0.33 to -0.29] | -0.31 [-0.33 to -0.28] | 0.03 [-0.00 to 0.06] | 0.03 [-0.00 to 0.06] |
| HAZ at discharge | -2.20 [-2.22 to -2.17] | -2.16 [-2.19 to -2.14] | -2.17 [-2.19 to -2.14] | 0.03 [0.00 to 0.07] | 0.03 [-0.00 to 0.06] |
| Proportion discharged with HAZ ≥ -2 | 43.5% [42.1 to 44.9] | 43.8% [42.2 to 45.3] | 42.9% [41.5 to 44.3] | 0.2 [-1.9 to 2.3] | -0.6 [-2.6 to 1.4] |
| **WAZ** |  |  |  |  |  |
| Change in WAZ from admission to discharge | 0.37 [0.34 to 0.40] | 0.33 [0.30 to 0.36] | 0.37 [0.35 to 0.41] | -0.03 [-0.08 to 0.01] | 0.01 [-0.04 to 0.06] |
| WAZ at discharge | -2.11 [-2.14 to -2.08] | -2.14 [-2.18 to -2.11] | -2.10 [-2.13 to -2.07] | -0.03 [-0.08 to 0.01] | 0.01 [-0.04 to 0.06] |
| Proportion discharged with WAZ ≥ -3 | 81.6% [79.7 to 83.5] | 82.6% [81.6 to 84.6] | 82.9% [80.9 to 84.8] | 1.0 [-1.8 to 3.8] | 1.3 [-1.5 to 4.0] |

Values are adjusted means or proportions with 95% confidence intervals derived from regression models. Continuous outcomes were modelled using linear regression and binary outcomes using logistic regression, adjusting for child age, sex, anthropometry, breastfeeding status, diarrhea, children in the household, and study location. All values are model-based estimates. Differences represent adjusted contrasts between study arms on the outcome scale (e.g., kg, cm, z-score units, or percentage points) and are defined as experimental arm minus control (1-RUSF).

MUAC = mid-upper arm circumference; WHZ = weight-for-height z-score; HAZ = height-for-age z-score; WAZ = weight-for-age z-score; RUTF = ready-to-use therapeutic food; RUSF = ready-to-use supplementary food.
