# Supplementary material for: Comparative effectiveness of lipid-nutrient spread supplementation regimens for moderate acute malnutrition in Ethiopia: The MODAM-MAM individually randomized controlled trial
Source: PLoS Med. 2026 Aug 11;23(8):e1005199. doi: 10.1371/journal.pmed.1005199 (PMC13475968; doi:10.1371/journal.pmed.1005199)
Supplement: Supplementary file 8 [file pmed.1005199.s007.docx]

**S7 Table: Growth outcomes among children *recovered* from moderate acute malnutrition by treatment group in intention-to-treat analysis**

|  | **1-RUSF** | **1-RUTF** | **2-RUTF** | **1-RUSF vs. 1-RUTF** | **1-RUSF vs. 2-RUTF** |
| --- | --- | --- | --- | --- | --- |
|  | n=683 | n=680 | n=726 | Difference | Difference |
| **Weight** |  |  |  |  |  |
| Change in weight from admission to discharge (kg) | 0.7 [0.5 to 1.0] | 0.7 [0.5 to 1.0] | 0.7 [0.5 to 1.0] | 0.03 [0.00 to 0.10] | 0.00 [-0.00 to 0.10] |
| Rate of weight gain (g/kg/d) | 1.64 [1.04 to 2.49] | 1.47 [0.93 to 2.32] | 1.54 [1.03 to 2.55] | 0.15 [0.04 to 0.25] | 0.01 [-0.10 to 0.12] |
| Rate of weight gain in the first 2  weeks (g/kg/d) | 1.83 [0.12 to 3.57] | 1.66 [0.02 to 3.20] | 1.88 [0.62 to 3.71] | 0.12 [-0.07 to 0.40] | -0.12 [-0.41 to 0.07] |
| Rate of weight gain in the first 4  weeks (g/kg/d) | 1.59 [0.69 to 2.64] | 1.53 [0.54 to 2.58] | 1.60 [0.59 to 2.78] | 0.08 [-0.07 to 0.25] | -0.01 [-0.18 to 0.14] |
| **MUAC** |  |  |  |  |  |
| Change in MUAC from admission to discharge (cm) | 0.7 [0.4 to 1.0] | 0.7 [0.4 to 1.0] | 0.7 [0.5 to 1.0] | 0.0 [-0.0 to 0.0] | 0.0 [-0.1 to 0.0] |
| Rate of MUAC gain (mm/d) | 0.12 [0.07 to 0.20] | 0.12 [0.07 to 0.19] | 0.14 [0.08 to 0.21] | 0.00 [-0.01 to 0.07] | -0.01 [-0.02 to -0.00] |
| MUAC at discharge (cm) | 13.0 [12.8 to 13.5] | 13.0 [12.8 to 13.5] | 13.0 [12.8 to 13.5] | 0.0 [-0.1 to 0.0] | 0.0 [-0.1 to 0.0] |
| **Length** |  |  |  |  |  |
| Change in length from admission to discharge (cm) | 0.5 [0.2 to 1.0] | 0.5 [0.2 to 1.2] | 0.5 [0.2 to 1.0] | 0.0 [-0.1 to 0.0] | 0.0 [-0.0 to 0.0] |
| Rate of length gain (mm/day) | 0.09 [0.04 to 0.18] | 0.09 [0.04 to 0.18] | 0.09 [0.04 to 0.19] | 0.00 [-0.01 to 0.01] | 0.00 [-0.01 to 0.00] |
| **WHZ** |  |  |  |  |  |
| Change in WHZ from admission to discharge | 0.86 [0.52 to 1.21] | 0.75 [0.43 to 1.10] | 0.80 [0.47 to 1.19] | 0.10 [0.04 to 0.15] | 0.05 [-0.01 to 0.10] |
| WHZ at discharge | -1.33 [-1.61 to -0.82] | -1.42 [-1.71 to -1.00] | -1.31 [-1.65 to -0.83] | 0.10 [0.05 to 0.16] | 0.01 [-0.04 to 0.06] |
| **HAZ** |  |  |  |  |  |
| Change in HAZ from admission to discharge | -0.25 [-0.46 to -0.12] | -0.21 [-0.44 to -0.08] | -0.25 [-0.46 to -0.10] | -0.04 [-0.01 to 0.02] | -0.01 [-0.04 to 0.02] |
| HAZ at discharge | -2.41 [-3.33 to -1.37] | -2.11 [-3.14 to -0.97] | -2.19 [-3.33 to -1.36] | -0.30 [-0.47 to -0.13] | -0.19 [-0.37 to -0.02] |
| Proportion discharged with HAZ ≥ -2 | 270 [39.8%] | 324 [48.1%] | 323 [44.8%] | 8.3 [3.0 to 13.6] | 5.0 [-0.1 to 10.2] |
| **WAZ** |  |  |  |  |  |
| Change in WAZ from admission to discharge | 0.44 [0.21 to 0.67] | 0.39 [0.18 to 0.62] | 0.41 [0.19 to 0.70] | 0.04 [0.00 to 0.08] | 0.01 [-0.03 to 0.50] |
| WAZ at discharge | -2.08 [-2.64 to -1.53] | -2.04 [-2.60 to -1.38] | -1.99 [-2.60 to -1.35] | -0.08 [-0.17 to 0.02] | -0.13 [-0.22 to -0.03] |
| Proportion discharged with WAZ ≥ -3 | 566 [83.4%] | 585 [86.8%] | 626 [86.8%] | 3.4 [-0.4 to 7.2] | 3.5 [-0.3 to 7.2] |

Data are n [%] or median [IQR], as appropriate. Differences are presented as median difference with 95% confidence intervals and were estimated using the Hodges-Lehmann method, which calculates the median of all pairwise differences between groups. Values reflect distributional shifts, not simple differences in medians. Risk difference is defined as experimental arm minus control (1-RUSF).

MUAC = mid-upper arm circumference; WHZ = weight-for-height z-score; HAZ = height-for-age z-score; WAZ = weight-for-age z-score; RUTF = ready-to-use therapeutic food; RUSF = ready-to-use supplementary food
