# Supplementary material for: Comparative effectiveness of lipid-nutrient spread supplementation regimens for moderate acute malnutrition in Ethiopia: The MODAM-MAM individually randomized controlled trial
Source: PLoS Med. 2026 Aug 11;23(8):e1005199. doi: 10.1371/journal.pmed.1005199 (PMC13475968; doi:10.1371/journal.pmed.1005199)
Supplement: Supplementary file 9 [file pmed.1005199.s008.docx]

**S8a Table: Primary outcomes for children treated for moderate acute malnutrition, stratified by age group in intention-to-treat analysis**

|  | **1-RUSF** | **1-RUTF** | **2-RUTF** | **1-RUSF vs. 1-RUTF** | **1-RUSF vs. 2-RUTF** | **1-RUTF vs. 2-RUTF** | |
| --- | --- | --- | --- | --- | --- | --- | --- |
|  |  |  |  | Difference | Difference | Difference | |
| **Children 6-23 months** | **n=449** | **n=407** | **n=476** |  |  |  |  |
| Recovered | 373 [83.1%] | 333 [81.8%] | 416 [87.4%] | -1.3 [-6.4 to 3.9] | 4.3 [-0.3 to 8.9] | 5.6 [0.8 to 10.4] |  |
| Not recovered | 76 [16.9%] | 74 [18.2%] | 60 [12.6%] | 1.3 [-3.9 to 6.4] | -4.3 [-8.9 to 0.3] | -5.6 [-10.4 to -0.8] |  |
| Deteriorated to SAM | 11 [2.5%] | 9 [2.2%] | 9 [1.9%] | -0.2 [-2.3 to 1.8] | -0.6 [-2.4 to 1.3] | -0.3 [-2.2 to 1.6] |  |
| Referred to Hospital | 4 [0.9%] | 12 [3.0%] | 14 [2.9%] | 2.1 [0.2 to 3.9] | 2.1 [0.3 to 3.8] | -0.0 [-2.2 to 2.2] |  |
| Remained with MAM | 33 [7.4%] | 29 [7.1%] | 18 [3.8%] | -0.2 [-3.7 to 3.3] | -3.6 [-6.5 to -0.6] | -3.3 [-6.4 to -0.3] |  |
| Defaulted | 26 [5.8%] | 22 [5.4%] | 18 [3.8%] | -0.4 [-3.5 to 2.7] | -2.0 [-4.8 to 0.8] | -1.6 [-4.4 to 1.2] |  |
| Died | 2 [0.5%] | 2 [0.5%] | 1 [0.2%] | 0.1 [-0.9 to 1.0] | -0.24 [-0.98 to 0.51] | -0.3 [-1.0 to 0.5] |  |
| Length of stay (d) | 56 [42 to 98] | 56 [42 to 84] | 56 [28 to 84] | 0 [0 to 0] | 0 [0 to 14] | 0 [0 to 0] |  |
| Total sachets provided | 70 [42 to 98] | 56 [42 to 84] | 112 [84 to 168] | 0 [0 to 0] | -56 [-56 to -42] | -56 [-56 to -56] |  |
| **Children 24-59 months** | **n=352** | **n=386** | **n=342** |  |  |  |  |
| Recovered | 306 [86.9%] | 341 [88.3%] | 305 [89.2%] | 1.4 [-3.4 to 5.3] | 2.3 [-2.6 to 7.1] | 0.8 [-3.8 to 5.4] |  |
| Not recovered | 46 [13.1%] | 45 [11.7%] | 37 [10.8%] | -1.4 [-5.2 to 3.4] | -2.3 [-7.1 to 2.6] | -0.8 [-5.4 to 3.8] |  |
| Deteriorated to SAM | 5 [1.4%] | 4 [1.0%] | 1 [0.3%] | -0.4 [-2.0 to 1.2] | -1.1 [-2.5 to 2.3] | -0.7 [-1.9 to 0.4] |  |
| Referred to Hospital | 2 [0.6%] | 0 [0.0%] | 2 [0.6%] | -0.6 [-1.4 to 0.2] | 0.0 [-1.1 to 1.1] | 0.6 [-0.2 to 1.4] |  |
| Remained with MAM | 26 [7.4%] | 26 [6.7%] | 21 [6.1%] | -0.7 [-4.4 to 3.1] | -1.3 [-5.0 to 2.5] | -0.6 [-4.2 to 3.0] |  |
| Defaulted | 13 [3.7%] | 15 [3.9%] | 12 [3.5%] | 0.2 [-2.6 to 3.0] | -0.2 [-3.0 to 2.6] | -0.4 [-3.1 to 2.4] |  |
| Died | 0 [0.0%] | 0 [0.0%] | 1 [0.3%] | 0.0 [0.0 to 0.0] | 0.3 [-0.3 to 0.9] | 0.3 [-0.3 to 0.9] |  |
| Length of stay (d) | 56 [28 to 98] | 56 [28 to 98] | 56 [28 to 88] | 0 [0 to 0] | 0 [0 to 0] | 0 [0 to 0] |  |
| Total sachets provided | 70 [42 to 98] | 70 [42 to 98] | 112 [84 to 196] | 0 [0 to 0] | -56 [-70 to -42] | -70 [-56 to -42] |  |

Data are n [%] or median [IQR], as appropriate. Effect estimates are presented as risk differences (percentage points) or median differences with 95% confidence intervals. Risk differences are calculated as experimental arm minus control (1-RUSF). Results are stratified by age group and should be interpreted as sub-group analyses.

MAM = moderate acute malnutrition; SAM = severe acute malnutrition; RUTF = ready-to-use therapeutic food; RUSF = ready-to-use supplementary food.

**S8b Table: Growth indicators for children treated for moderate acute malnutrition, stratified by age group in intention-to-treat analysis**

|  | **1-RUSF** | **1-RUTF** | **2-RUTF** | **1-RUSF vs. 1-RUTF** | **1-RUSF vs. 2-RUTF** | **1-RUTF vs. 2-RUTF** |
| --- | --- | --- | --- | --- | --- | --- |
|  |  |  |  | Difference | Difference | Difference |
| **Children 6-23 months** | **n=449** | **n=406** | **n=476** |  |  |  |
| Weight gain (kg) | 0.70 [0.40 to 0.96] | 0.63 [0.40 to 0.90] | 0.70 [0.40 to 1.00] | -0.01 [-0.00 to 0.10] | 0.00 [-0.05 to 0.10] | -0.00 [-0.10 to 0.00] |
| Rate of weight gain (g/kg/d) | 1.62 [0.84 to 2.49] | 1.56 [0.85 to 2.49] | 1.60 [0.91 to 2.76] | 0.06 [-0.11 to 0.23] | -0.11 [-0.29 to 0.06] | -0.17 [-0.35 to 0.00] |
| Rate of weight gain in the first 2  weeks (g/kg/d) | 2.00 [0.13 to 3.90] | 1.74 [0.00 to 3.57] | 2.01 [0.01 to 3.91] | 0.24 [0.00 to 0.74] | -0.01 [-0.50 to 0.23] | -0.31 [-0.82 to 0.00] |
| MUAC gain (cm) | 0.7 [0.3 to 1.0] | 0.6 [0.3 to 1.0] | 0.7 [0.4 to 0.9] | 0.0 [-0.1 to 0.1] | 0.0 [-0.1 to 0.0] | 0.0 [-0.1 to 0.0] |
| MUAC at discharge (cm) | 12.8 [12.6 to 13.1] | 12.8 [12.6 to 13.1] | 12.9 [12.7 to 13.1] | 0.0 [-0.1 to 0.0] | -0.1 [-0.1 to 0.0] | 0.0 [-0.1 to 0.0] |
| Length gain (cm) | 0.5 [0.2 to 1.2] | 0.5 [0.2 to 1.3] | 0.5 [0.2 to 1.1] | 0.0 [-0.1 to 0.1] | 0.0 [0.0 to 0.1] | 0.0 [0.0 to 0.1] |
| Change in WHZ | 0.86 [0.39 to 1.28] | 0.74 [0.35 to 1.17] | 0.82 [0.36 to 1.27] | 0.09 [0.00 to 0.18] | 0.02 [-0.07 to 0.12] | -0.07 [-0.16 to 0.03] |
| WHZ at discharge | -1.20 [-1.61 to -0.66] | -1.26 [-1.71 to -0.79] | -1.16 [-1.62 to -0.68] | 0.07 [-0.03 to 0.17] | -0.01 [-0.11 to 0.08] | -0.09 [-0.18 to 0.01] |
| Change in HAZ | -0.42 [-0.73 to -0.24] | -0.44 [-0.67 to -0.22] | -0.40 [-0.65 to -0.21] | -0.02 [-0.07 to 0.03] | -0.04 [-0.08 to 0.01] | -0.02 [-0.07 to 0.03] |
| HAZ at discharge | -2.71[-3.47 to -1.74] | -2.42 [-3.34 to -1.30] | -2.50 [-3.42 to -1.49] | -0.30 [-0.49 to -0.10] | -0.17 [-0.36 to 0.03] | 0.13 [-0.07 to 0.33] |
| Change in WAZ | 0.37 [0.05 to 0.68] | 0.34 [0.01 to 0.63] | 0.37 [0.05 to 0.74] | 0.03 [-0.03 to 0.10] | -0.02 [-0.08 to 0.05] | -0.05 [-0.12 to 0.02] |
| WAZ at discharge | -2.16 [-2.79 to -1.61] | -2.10 [-2.62 to -1.51] | -2.06 [-2.72 to -1.48] | -0.10 [-0.22 to 0.02] | -0.11 [-0.23 to 0.01] | -0.01 [-0.13 to 0.11] |
| **Children 24-59 months** | **n=352** | **n=386** | **n=342** |  |  |  |
| Weight gain (kg) | 0.72 [0.50 to 1.04] | 0.70 [0.40 to 1.00] | 0.76 [0.50 to 1.00] | 0.10 [0.00 to 0.10] | 0.00 [-0.00 to 0.10] | -0.00 [-0.10 to 0.00] |
| Rate of weight gain (g/kg/d) | 1.28 [0.81 to 2.09] | 1.13 [0.68 to 1.81] | 1.21 [0.76 to 1.91] | 0.17 [0.05 to 0.29] | 0.06 [-0.06 to 0.19] | -0.10 [-0.22 to 0.01] |
| Rate of weight gain in the first 2  weeks (g/kg/d) | 1.34 [0.00 to 2.78] | 1.35 [0.39 to 2.72] | 1.39 [0.52 to 3.04] | -0.36 [-0.02 to 0.23] | -0.14 [-0.53 to 0.12] | -0.09 [-0.42 to 0.14] |
| MUAC gain (cm) | 0.6 [0.4 to 0.9] | 0.6 [0.3 to 0.9] | 0.7 [0.4 to 1.0] | 0.0 [-0.1 to 0.1] | -0.1 [-0.1 to 0.0] | -0.1 [-0.1 to 0.0] |
| MUAC at discharge (cm) | 13.3 [13.0 to 13.9] | 13.3 [12.9 to 13.9] | 13.4 [13.0 to 13.9] | 0.0 [-0.1 to 0.1] | -0.1 [-0.2 to 0.0] | -0.1 [-0.2 to 0.0] |
| Length gain (cm) | 0.5 [0.2 to 1.0] | 0.5 [0.1 to 1.3] | 0.5 [0.2 to 1.0] | 0.0 [-0.1 to 0.1] | 0.0 [-0.1 to 0.0] | 0.0 [-0.1 to 0.1] |
| Change in WHZ | 0.69 [0.45 to 1.00] | 0.63 [0.37 to 0.93] | 0.66 [0.40 to 0.97] | 0.08 [0.02 to 0.15] | 0.05 [-0.02 to 0.11] | -0.04 [-0.10 to 0.03] |
| WHZ at discharge | -1.52 [-1.76 to -1.19] | -1.61 [-1.82 to -1.33] | -1.60 [-1.75 to -1.28] | 0.08 [0.02 to 0.14] | 0.03 [-0.03 to 0.09] | -0.05 [-0.10 to 0.01] |
| Change in HAZ | -0.13 [-0.23 to -0.02] | -0.14 [-0.22 to -0.03] | -0.12 [-0.23 to 0.00] | 0.00 [-0.03 to 0.02] | -0.01 [-0.03 to 0.02] | -0.01 [-0.03 to 0.02] |
| HAZ at discharge | -1.97 [-3.17 to -0.84] | -1.81 [-3.04 to -0.60] | -1.85 [-2.90 to -0.56] | 0.19 [-0.45 to 0.06] | -0.20 [-0.47 to 0.08] | -0.01 [-0.28 to 0.26] |
| Change in WAZ | 0.39 [0.21 to 0.59] | 0.33 [0.16 to 0.53] | 0.38 [0.17 to 0.56] | 0.06 [0.01 to 0.10] | 0.02 [-0.02 to 0.07] | -0.03 [-0.08 to 0.01] |
| WAZ at discharge | -2.13 [-2.76 to -1.48] | -2.11 [-2.79 to -1.35] | -2.04 [-2.77 to -1.32] | -0.05 [-0.20 to 0.10] | -0.11 [-0.26 to 0.04] | -0.05 [-0.21 to 0.10] |

Data are median [IQR]. Differences are presented as median difference with 95% confidence intervals. Differences are defined as experimental arm minus control (1-RUSF). Results are stratified by age group and should be interpreted as sub-analyses. MUAC = mid-upper arm circumference; WHZ = weight-for-height z-score; HAZ = height-for-age z-score; WAZ = weight-for-age z-score; RUTF = ready-to-use therapeutic food; RUSF = ready-to-use supplementary food.
