# Supplementary material for: Comparative effectiveness of lipid-nutrient spread supplementation regimens for moderate acute malnutrition in Ethiopia: The MODAM-MAM individually randomized controlled trial
Source: PLoS Med. 2026 Aug 11;23(8):e1005199. doi: 10.1371/journal.pmed.1005199 (PMC13475968; doi:10.1371/journal.pmed.1005199)
Supplement: Supplementary file 10 [file pmed.1005199.s009.docx]

**S9a Table: Primary Outcomes for children treated for moderate acute malnutrition, stratified by MUAC on admission in intention-to-treat analysis**

|  | **1-RUSF** | **1-RUTF** | **2-RUTF** | **1-RUSF vs. 1-RUTF** | **1-RUSF vs. 2-RUTF** | **1-RUTF vs. 2-RUTF** |  |
| --- | --- | --- | --- | --- | --- | --- | --- |
|  |  |  |  | Difference | Difference | Difference |  |
| **Admitted with MUAC 11.5 – 11.9cm** | **n=125** | **n=109** | **n=121** |  |  |  | |
| Recovered | 92 [73.6%] | 84 [77.1%] | 92 [76.0%] | 3.5 [-2.6 to 14.5] | 2.4 [-8.4 to 13.3] | -1.0 [-12.0 to 9.9] | |
| Not recovered | 33 [26.4%] | 25 [22.9%] | 29 [24.0%] | -3.5 [-14.5 to 2.6] | -2.4 [-13.3 to 8.4] | 1.0 [-9.9 to 12.0] | |
| Deteriorated to SAM | 4 [3.2%] | 4 [3.7%] | 7 [5.8%] | 0.5 [-4.2 to 5.2] | 2.6 [-2.6 to 7.8] | 2.1 [-2.2 to 7.6] | |
| Referred to Hospital | 2 [1.6%] | 5 [4.6%] | 6 [5.0%] | 3.0 [-1.5 to 7.5] | 3.4 [-1.1 to 7.8] | 0.4 [-5.1 to 5.9] | |
| Remained with MAM | 20 [16.0%] | 14 [12.8%] | 10 [8.3%] | -3.2 [-12.1 to 5.9] | -7.7 [-15.8 to 0.4] | -4.6 [-12.6 to 3.4] | |
| Defaulted | 7 [5.6%] | 1 [0.9%] | 6 [5.0%] | -4.7 [-9.1 to -0.3] | -0.6 [-6.2 to 5.0] | 4.0 [-0.2 to 8.3] | |
| Died | 0 [0.0%] | 1 [0.9%] | 0 [0.0%] | 0.9 [-0.9 to 2.7] | 0.0 [0.0 to 0.0] | -0.9 [-2.7 to 0.9] | |
| Length of stay (d) | 70 [56 to 112] | 70 [42 to 98] | 70 [42 to 98] | 0 [0 to 14] | 0 [0 to 14] | 0 [-1 to 0] | |
| Total sachets provided | 84 [56 to 112] | 84 [56 to 112] | 140 [112 to 210] | 0 [0 to 14] | -70 [-84 to -56] | -70 [-84 to -56] | |
| **Admitted with MUAC ≥ 12.0 cm** | **n=676** | **n=684** | **n=629** |  |  |  | |
| Recovered | 587 [86.8%] | 590 [86.3%] | 629 [90.2%] | -0.6 [-4.2 to 3.1] | 3.4 [0.0 to 6.8] | 4.0 [0.6 to 7.4] | |
| Not recovered | 89 [13.2%] | 94 [13.7%] | 68 [9.8%] | 0.6 [-3.1 to 4.2] | -3.4 [-6.8 to -0.4] | -4.0 [-7.4 to -0.6] | |
| Deteriorated to SAM | 12 [1.8%] | 9 [1.3%] | 3 [0.4%] | -0.5 [-1.8 to 0.9] | -1.3 [-2.5 to -0.2] | -0.9 [-1.9 to 0.1] | |
| Referred to Hospital | 4 [0.6%] | 7 [1.0%] | 10 [1.4%] | 0.4 [-0.5 to 1.4] | 0.8 [-0.2 to 1.9] | 0.4 [-0.8 to 1.6] | |
| Remained with MAM | 39 [5.8%] | 41 [6.0%] | 29 [4.2%] | 0.2 [-2.3 to 2.7] | -1.6 [-3.1 to 0.7] | -1.8 [-4.2 to 0.5] | |
| Defaulted | 32 [4.7%] | 36 [5.3%] | 24 [3.4%] | 0.5 [-1.8 to 2.9] | -1.3 [-3.4 to 0.8] | -1.8 [-4.0 to 0.3] | |
| Died | 2 [0.3%] | 1 [0.2%] | 2 [0.3%] | -0.2 [-0.7 to 0.4] | -0.0 [-0.6 to 0.6] | 0.1 [-0.4 to 0.6] | |
| Length of stay (d) | 56 [28 to 84] | 56 [28 to 84] | 42 [28 to 84] | 0 [0 to 0] | 0 [0 to 0] | 0 [0 to 0] | |
| Total sachets provided | 56 [42 to 98] | 56 [42 to 96] | 112 [84 to 168] | 0 [0 to 0] | -56 [-56 to -42] | -56 [-56 to -42] | |

Data are n [%] or median [IQR], as appropriate. Effect estimates are presented as risk differences (percentage points) or median differences with 95% confidence intervals. Risk difference is calculated as experimental arm minus control (1-RUSF). Results are stratified by MUAC at admission and should be interpreted as sub-analyses.

MAM = moderate acute malnutrition; SAM = severe acute malnutrition; RUTF = ready-to-use therapeutic food; RUSF = ready-to-use supplementary food.

**S9b Table: Growth indicators for children treated for moderate acute malnutrition, stratified by MUAC on admission in intention-to-treat analysis**

|  | **1-RUSF** | **1-RUTF** | **2-RUTF** | **1-RUSF vs. 1-RUTF** | **1-RUSF vs. 2-RUTF** | **1-RUTF vs. 2-RUTF** |  |
| --- | --- | --- | --- | --- | --- | --- | --- |
|  |  |  |  | Difference | Difference | Difference |  |
| **Admitted with MUAC 11.5 – 11.9cm** | **n=125** | **n=108** | **n=121** |  |  |  | |
| Weight gain (kg) | 0.9 [0.5 to 1.2] | 0.8 [0.5 to 1.1] | 0.7 [0.4 to 1.1] | 0.00 [-0.10 to 0.20] | 0.10 [-0.01 to 0.23] | 0.10 [-0.10 to 0.20] | |
| Rate of weight gain (g/kg/d) | 1.70 [0.85 to 2.46] | 1.69 [0.91 to 2.50] | 1.38 [0.74 to 2.46] | -0.01 [-0.34 to 0.33] | 0.17 [-0.19 to 0.49] | 0.17 [-0.18 to 0.52] | |
| Rate of weight gain in the first 2  weeks (g/kg/d) | 2.12 [0.00 to 4.06] | 2.16 [0.78 to 4.32] | 1.86 [0.00 to 3.69] | -0.22 [-1.02 to 0.42] | 0.13 [-0.53 to 0.95] | 0.47 [-0.21 to 1.12] | |
| MUAC gain (cm) | 1.0 [0.8 to 1.2] | 1.0 [0.8 to 1.2] | 1.0 [0.7 to 1.2] | 0.0 [-0.1 to 0.1] | 0.0 [-0.1 to 0.1] | 0.0 [-0.1 to 0.1] | |
| MUAC at discharge (cm) | 12.7 [12.5 to 12.9] | 12.8 [12.6 to 12.9] | 12.7 [12.5 to 13] | 0.0 [-0.1 to 0.0] | 0.0 [-0.1 to 0.1] | 0.0 [-0.1 to 0.1] | |
| Length gain (cm) | 0.8 [0.4 to 1.8] | 0.6 [0.3 to 1.4] | 0.8 [0.3 to 1.7] | -0.1 [-0.0 to 0.3] | 0.0 [-0.2 to 0.2] | -0.1 [-0.3 to 0.1] | |
| Change in WHZ | 1.09 [0.31 to 1.57] | 0.89 [0.46 to 1.44] | 0.82 [0.18 to 1.42] | 0.07 [-0.17 to 0.30] | 0.21 [-0.03 to 0.45] | 0.14 [-0.08 to 0.38] | |
| WHZ at discharge | -1.10 [-1.58 to -0.67] | -1.03 [-1.56 to -0.52] | -1.19 [-1.79 to -0.61] | -0.10 [-0.32 to 0.11] | 0.06 [-0.16 to 0.29] | 0.17 [-0.06 to 0.42] | |
| Change in HAZ | -0.47 [-0.89 to -0.24] | -0.43 [-0.73 to -0.21] | -0.36 [-0.74 to -0.15] | -0.07 [-0.17 to 0.05] | -0.12 [-0.23 to 0.00] | -0.05 [-0.16 to 0.06] | |
| HAZ at discharge | -3.04 [-3.94 to -2.29] | -3.11 [-4.08 to -2.08] | -3.04 [-3.96 to -2.12] | 0.07 [-0.27 to 0.42] | -0.09 [-0.44 to 0.26] | -0.17 [-0.53 to 0.21] | |
| Change in WAZ | 0.52 [0.06 to 0.87] | 0.49 [0.06 to 0.78] | 0.36 [-0.03 to 0.80] | 0.00 [-0.16 to 0.15] | 0.09 [-0.07 to 0.24] | 0.09 [-0.06 to 0.24] | |
| WAZ at discharge | -2.46 [-3.02 to -1.83] | -2.42 [-2.96 to -1.84] | -2.44 [-3.18 to -1.92] | -0.04 [-0.26 to 0.19] | 0.04 [-0.21 to 0.27] | 0.07 [-0.19 to 0.31] | |
| **Admitted with MUAC ≥ 12 cm** | **n=676** | **n=684** | **n=697** |  |  |  | |
| Weight gain (kg) | 0.7 [0.5 to 1.0] | 0.7 [0.4 to 0.9] | 0.7 [0.4 to 1.0] | 0.03 [0.00 to 0.10] | 0.00 [-0.02 to 0.04] | -0.02 [-0.10 to 0.00] | |
| Rate of weight gain (g/kg/d) | 1.37 [0.83 to 2.29] | 1.23 [0.70 to 2.07] | 1.41 [0.85 to 2.41] | 0.15 [0.04 to 0.26] | -0.05 [-0.16 to 0.06] | -0.20 [-0.3 to -0.09] | |
| Rate of weight gain in the first 2  weeks (g/kg/d) | 1.58 [0.00 to 3.42] | 1.35 [0.00 to 1.82] | 1.76 [0.21 to 3.51] | 0.18 [0.00 to 0.46] | -0.13 [-0.44 to 0.06] | -0.36 [-0.64 to -0.09] | |
| MUAC gain (cm) | 0.6 [0.2 to 0.8] | 0.6 [0.3 to 0.8] | 0.6 [0.4 to 0.9] | 0.0 [-0.0 to 0.0] | -0.0 [-0.1 to 0.0] | -0.0 [-0.1 to 0.0] | |
| MUAC at discharge (cm) | 13.0 [12.8 to 13.5] | 13.0 [12.8 to 13.6] | 13.1 [12.8 to 13.6] | 0.0 [-0.1 to 0.0] | -0.1 [-0.1 to 0.0] | 0.0 [-0.1 to 0.0] | |
| Length gain (cm) | 0.5 [0.2 to 1.0] | 0.5 [0.1 to 1.2] | 0.5 [0.2 to 1.0] | 0.0 [-0.1 to 0.0] | 0.0 [0.0 to 0.1] | 0.0 [0.0 to 0.1] | |
| Change in WHZ | 0.73 [0.43 to 1.09] | 0.64 [0.36 to 0.99] | 0.72 [0.40 to 1.11] | 0.09 [0.04 to 0.15] | 0.01 [-0.05 to 0.05] | -0.08 [-0.14 to -0.03] | |
| WHZ at discharge | -1.43 [-1.71 to -0.99] | -1.52 [-1.80 to -1.11] | -1.42 [-1.70 to -0.96] | 0.11 [0.05 to 0.17] | -0.01 [-0.07 to 0.05] | -0.12 [-0.18 to -0.07] | |
| Change in HAZ | -0.23 [-0.42 to -0.10] | -0.20 [-0.43 to -0.08] | -0.24 [-0.45 to -0.09] | -0.02 [-0.05 to 0.00] | 0.00 [-0.03 to 0.02] | 0.02 [-0.01 to 0.05] | |
| HAZ at discharge | -1.92 [-2.96 to -0.85] | -2.10 [-3.19 to -0.99] | -2.11 [-2.73 to -1.49] | -0.32 [-0.49 to -0.15] | -0.18 [-0.36 to 0.00] | 0.14 [-0.04 to 0.32] | |
| Change in WAZ | 0.36 [0.14 to 0.60] | 0.32 [0.11 to 0.55] | 0.38 [0.14 to 0.63] | 0.01 [0.05 to 0.80] | -0.01 [-0.05 to 0.03] | -0.06 [-0.10 to -0.02] | |
| WAZ at discharge | -2.11 [-2.73 to -1.49] | -2.06 [-2.69 to -1.36] | -1.99 [-2.64 to -1.36] | -0.08 [-0.18 to 0.02] | -0.13 [-0.23 to -0.03] | -0.05 [-0.15 to 0.05] | |

Data are median [IQR]. Differences are presented as median difference with 95% confidence intervals. Differences are defined as experimental arm minus control (1-RUSF). Results are stratified by MUAC at admission and should be interpreted as sub-analyses.

MUAC = mid-upper arm circumference; WHZ = weight-for-height z-score; HAZ = height-for-age z-score; WAZ = weight-for-age z-score; RUTF = ready-to-use therapeutic food; RUSF = ready-to-use supplementary food.
