# Supplementary material for: Comparative effectiveness of lipid-nutrient spread supplementation regimens for moderate acute malnutrition in Ethiopia: The MODAM-MAM individually randomized controlled trial
Source: PLoS Med. 2026 Aug 11;23(8):e1005199. doi: 10.1371/journal.pmed.1005199 (PMC13475968; doi:10.1371/journal.pmed.1005199)
Supplement: Supplementary file 11 [file pmed.1005199.s010.docx]

**S10a Table: Primary Outcomes for children treated for moderate acute malnutrition, stratified by WAZ on admission in intention-to-treat analysis**

|  | **1-RUSF** | **1-RUTF** | **2-RUTF** | **1-RUSF vs. 1-RUTF** | **1-RUSF vs. 2-RUTF** | **1-RUTF vs. 2-RUTF** |
| --- | --- | --- | --- | --- | --- | --- |
|  |  |  |  | Difference | Difference | Difference |
| **Admitted with WAZ < -3** | **n=266** | **n=221** | **n=240** |  |  |  |
| Recovered | 226 [85.0%] | 187 [84.6%] | 205 [85.4%] | -0.3 [-6.8 to 6.1] | 0.5 [-5.7 to 6.7] | 0.8 [-5.7 to 7.3] |
| Not recovered | 40 [15.0%] | 34 [15.4%] | 35 [14.6%] | 0.3 [-6.1 to 6.8] | -0.5 [-6.7 to 5.7] | -0.8 [-7.3 to 5.7] |
| Deteriorated to SAM | 5 [1.9%] | 6 [2.7%] | 2 [0.8%] | 0.8 [-1.9 to 3.5] | -1.1 [-3.0 to 1.0] | -1.9 [-4.3 to 0.6] |
| Referred to Hospital | 4 [1.5%] | 5 [2.3%] | 6 [2.5%] | 0.8 [-1.7 to 3.2] | 1.0 [-1.5 to 3.5] | 0.2 [-2.6 to 3.0] |
| Remained with MAM | 18 [6.8%] | 13 [5.9%] | 12 [5.0%] | -0.9 [-5.2 to 3.4] | -1.8 [-5.9 to 2.3] | -0.9 [-5.0 to 3.3] |
| Defaulted | 12 [4.5%] | 8 [3.6%] | 13 [5.4%] | -0.9 [-4.4 to 2.6] | 0.9 [-2.9 to 4.7] | 1.8 [-2.0 to 5.6] |
| Died | 1 [0.4%] | 2 [0.9%] | 2 [0.8%] | 0.5 [-0.9 to 2.0] | 0.5[-0.9 to 1.8] | -0.1 [-1.8 to 1.6] |
| Length of stay (d) | 56 [42 to 98] | 56 [42 to 84] | 56 [42 to 84] | 0 [0 to 0] | 0 [0 to 14] | 0 [0 to 0] |
| Total sachets provided | 70 [42 to 98] | 70 [42 to 91] | 112 [84 to 196] | 0 [0 to 7] | -56 [-70 to -49] | -56 [-70 to -56] |
| **Admitted with WAZ ≥ -3** | **n=535** | **n=572** | **n=578** |  |  |  |
| Recovered | 453 [84.7%] | 487 [85.1%] | 516 [89.3%] | 0.5 [-3.8 to 4.7] | 4.6 [0.6 to 8.6] | 4.1 [0.3 to 8.0] |
| Not recovered | 82 [15.3%] | 85 [14.9%] | 62 [10.7%] | -0.5 [-4.7 to 3.8] | -4.6 [-8.6 to -0.6] | -4.1 [-8.0 to -0.3] |
| Deteriorated to SAM | 11 [2.1%] | 7 [1.2%] | 8 [1.4%] | -0.8 [-2.3 to 0.7] | -0.7 [-2.2 to 0.9] | 0.2 [-1.2 to 1.5] |
| Referred to Hospital | 2 [0.4%] | 7 [1.2%] | 10 [1.7%] | 0.9 [-0.2 to 1.9] | 1.4 [0.2 to 2.5] | 0.5 [-0.9 to 1.9] |
| Remained with MAM | 41 [7.7%] | 42 [7.3%] | 27 [4.7%] | -0.3 [-3.4 to 2.8] | -3.0 [-5.8 to -0.2] | -2.7 [-5.4 to 0.1] |
| Defaulted | 27 [5.0%] | 29 [5.1%] | 17 [2.9%] | 0.0 [-2.6 to 2.5] | -2.1 [-4.4 to 0.2] | -2.1 [-4.4 to 0.1] |
| Died | 1 [0.2%] | 0 [0.0%] | 0 [0.0%] | -0.2 [-0.6 to 0.2] | -0.2 [-0.6 to 0.2] | 0.0 [0.0 to 0.0] |
| Length of stay (d) | 56 [42 to 84] | 56 [30 to 84] | 56 [28 to 84] | 0 [0 to 0] | 0 [0 to 0] | 0 [0 to 0] |
| Total sachets provided | 70 [42 to 98] | 70 [42 to 98] | 112 [84 to 196] | 0 [0 to 0] | -56 [-56 to -42] | -56 [-56 to -42] |

Data are n [%] or median [IQR], as appropriate. Effect estimates are presented as risk differences (percentage points) or median differences with 95% confidence intervals. Risk difference is calculated as experimental arm minus control (1-RUSF). Results are stratified by WAZ at admission and should be interpreted as sub-analyses.

MAM = moderate acute malnutrition; SAM = severe acute malnutrition; RUTF = ready-to-use therapeutic food; RUSF = ready-to-use supplementary food.

**S10b Table: Growth indicators for children treated for moderate acute malnutrition, stratified by WAZ on admission in intention-to-treat analysis**

|  | **1-RUSF** | **1-RUTF** | **2-RUTF** | **1-RUSF vs. 1-RUTF** | **1-RUSF vs. 2-RUTF** | **1-RUTF vs. 2-RUTF** |
| --- | --- | --- | --- | --- | --- | --- |
|  |  |  |  | Difference | Difference | Difference |
| **Admitted with WAZ < -3** | **n=266** | **n=221** | **n=240** |  |  |  |
| Weight gain (kg) | 0.8 [0.5 to 1.0] | 0.7 [0.5 to 1.0] | 0.8 [0.4 to 1.0] | 0.00 [-0.08 to 0.10] | 0.0 [-0.1 to 0.1] | 0.0 [-0.1 to 0.1] |
| Rate of weight gain (g/kg/d) | 1.72 [0.99 to 2.73] | 1.67 [0.98 to 2.56] | 1.56 [1.05 to 2.61] | 0.07 [-0.15 to 0.30] | 0.01 [-0.21 to 0.23] | -0.05 [-0.27 to 0.17] |
| Rate of weight gain in the first 2  weeks (g/kg/d) | 2.02 [0.69 to 4.06] | 2.01 [0.68 to 3.86] | 1.95 [0.00 to 4.01] | 0.00 [-0.50 to 0.47] | 0.00 [-0.53 to 0.44] | 0.00 [-0.54 to 0.47] |
| MUAC gain (cm) | 0.6 [0.3 to 1.0] | 0.7 [0.3 to 1.0] | 0.7 [0.4 to 1.0] | 0.0 [-0.1 to 0.1] | -0.1 [-0.1 to 0.0] | -0.0 [-0.1 to 0.0] |
| MUAC at discharge (cm) | 12.9 [12.7 to 13.2] | 12.9 [12.7 to 13.2] | 12.9 [12.7 to 13.2] | 0.0 [-0.1 to 0.0] | 0.0 [-0.1 to 0.0] | 0.0 [-0.1 to 0.1] |
| Length gain (cm) | 0.50 [0.20 to 1.10] | 0.50 [0.20 to 1.30] | 0.50 [0.20 to 1.10] | 0.0 [-0.1 to 0.1] | 0.0 [-0.1 to 0.1] | 0.0 [-0.1 to 0.1] |
| Change in WHZ | 0.98 [0.55 to 1.34] | 0.87 [0.48 to 1.27] | 0.95 [0.50 to 1.37] | 0.10 [-0.02 to 0.22] | 0.03 [-0.08 to 0.15] | -0.06 [-0.18 to 0.06] |
| WHZ at discharge | -1.37 [-1.73 to -0.82] | -1.50 [-1.82 to -0.99] | -1.31 [-1.71 to -0.84] | 0.12 [0.01 to 0.23] | -0.02 [-0.13 to 0.10] | -0.14 [-0.26 to -0.02] |
| Change in HAZ | -0.23 [-0.49 to -0.10] | -0.17 [-0.35 to -0.02] | -0.19 [-0.43 to -0.01] | -0.08 [-0.13 to -0.03] | -0.07 [-0.12 to -0.01] | 0.02 [-0.04 to 0.08] |
| HAZ at discharge | -3.73 [-4.51 to -3.12] | -3.63 [-4.34 to -3.07] | -3.82 [-4.5 to -3.2] | -0.10 [-0.26 to 0.07] | 0.04 [-0.12 to 0.21] | 0.14 [-0.03 to 0.31] |
| Change in WAZ | 0.52 [0.19 to 0.76] | 0.46 [0.26 to 0.74] | 0.51 [0.23 to 0.77] | 0.00 [-0.07 to 0.08] | -0.02 [-0.09 to 0.06] | -0.02 [-0.09 to 0.06] |
| WAZ at discharge | -3.04 [-3.39 to -2.60] | -3.02 [-3.48 to -2.67] | -2.99 [-3.40 to -2.60] | 0.01 [-0.09 to 0.12] | 0.00 [-0.11 to 0.11] | -0.01 [-0.13 to 0.10] |
| **Admitted with WAZ ≥ -3** | **n=535** | **n=571** | **n=578** |  |  |  |
| Weight gain (kg) | 0.7 [0.4 to 1.0] | 0.7 [0.4 to 0.9] | 0.7 [0.4 to 1.0] | 0.05 [0.00 to 0.10] | 0.00 [-0.00 to 0.10] | -0.0 [-0.1 to 0.0] |
| Rate of weight gain (g/kg/d) | 1.32 [0.81 to 2.16] | 1.17 [0.68 to 1.93] | 1.35 [0.73 to 2.35] | 0.13 [0.02 to 0.24] | -0.04 [-0.17 to 0.08] | -0.17 [-0.29 to -0.05] |
| Rate of weight gain in the first 2  weeks (g/kg/d) | 1.41 [0.00 to 3.09] | 1.22 [0.00 to 2.73] | 1.60 [0.20 to 3.32] | 0.13 [-0.07 to 0.44] | -0.15 [-0.49 to 0.05] | -0.34 [-0.62 to -0.05] |
| MUAC gain (cm) | 0.6 [0.3 to 0.9] | 0.6 [0.3 to 0.9] | 0.7 [0.4 to 0.9] | 0.0 [-0.0 to 0.1] | -0.0 [-0.1 to 0.0] | -0.0 [-0.1 to 0.0] |
| MUAC at discharge (cm) | 13.0 [12.7 to 13.5] | 13.0 [12.7 to 13.6] | 13.1 [12.8 to 13.6] | 0.0 [-0.1 to 0.0] | -0.1 [-0.1 to 0.0] | 0.0 [-0.1 to 0.0] |
| Length gain (cm) | 0.50 [0.20 to 1.10] | 0.50 [0.20 to 1.13] | 0.50 [0.20 to 1.03] | 0.0 [-0.0 to 0.1] | 0.0 [0.0 to 0.1] | 0.0 [0.0 to 0.1] |
| Change in WHZ | 0.68 [0.39 to 1.05] | 0.62 [0.30 to 0.97] | 0.67 [0.36 to 1.03] | 0.07 [0.01 to 0.13] | 0.02 [-0.05 to 0.08] | -0.05 [-0.12 to 0.01] |
| WHZ at discharge | -1.41 [-1.70 to -0.93] | -1.46 [-1.77 to -1.05] | -1.42 [-1.71 to -0.91] | 0.08 [0.02 to 0.15] | 0.01 [-0.06 to 0.07] | -0.08 [-0.14 to -0.01] |
| Change in HAZ | -0.26 [-0.49 to -0.12] | -0.25 [-0.52 to -0.11] | -0.27 [-0.49 to -0.12] | -0.01 [-0.05 to 0.02] | 0.00 [-0.03 to 0.04] | 0.01 [-0.02 to 0.05] |
| HAZ at discharge | -1.76 [-2.47 to -0.83] | -1.49 [-2.39 to -0.54] | -1.63 [-2.39 to -0.61] | -0.18 [-0.34 to -0.03] | -0.12 [-0.27 to 0.03] | 0.06 [-0.10 to 0.22] |
| Change in WAZ | 0.34 [0.12 to 0.57] | 0.29 [0.06 to 0.53] | 0.33 [0.08 to 0.58] | 0.05 [0.00 to 0.09] | 0.00 [-0.04 to 0.05] | -0.04 [-0.09 to 0.00] |
| WAZ at discharge | -1.81 [-2.21 to -1.30] | -1.78 [-2.25 to -1.22] | -1.72 [-2.15 to -1.19] | -0.03 [-0.11 to 0.06] | -0.08 [-0.17 to 0.00] | -0.06 [-0.14 to 0.03] |

Data are median [IQR]. Differences are presented as median difference with 95% confidence intervals. Differences are defined as experimental arm minus control (1-RUSF). Results are stratified by WAZ at admission and should be interpreted as sub-analyses.

MUAC = mid-upper arm circumference; WHZ = weight-for-height z-score; HAZ = height-for-age z-score; WAZ = weight-for-age z-score; RUTF = ready-to-use therapeutic food; RUSF = ready-to-use supplementary food.
