# Supplementary material for: Comparative effectiveness of lipid-nutrient spread supplementation regimens for moderate acute malnutrition in Ethiopia: The MODAM-MAM individually randomized controlled trial
Source: PLoS Med. 2026 Aug 11;23(8):e1005199. doi: 10.1371/journal.pmed.1005199 (PMC13475968; doi:10.1371/journal.pmed.1005199)
Supplement: Supplementary file 12 [file pmed.1005199.s011.docx]

**S11a Table: Outcomes among children treated for moderate acute malnutrition by study site across all treatment arms in intention-to-treat analysis**

|  | **Gode** | **Teltele** |  |
| --- | --- | --- | --- |
|  | n=1,195 | n=1,217 | Difference |
| Recovered | 1,044 [87.4%] | 1,030 [84.6%] | -2.7 [-5.5 to 0.0] |
| Not recovered | 151 [12.6%] | 187 [15.4%] | 2.7 [-0.0 to 5.5] |
| Deteriorated to SAM | 16 [1.3%] | 23 [1.9%] | 0.6 [-0.5 to 1.6] |
| Referred to Hospital | 20 [1.7%] | 14 [1.2%] | -0.5 [-1.5 to 0.4] |
| Remained with MAM | 36 [3.0%] | 117 [9.6%] | 6.6 [4.7 to 8.5] |
| Defaulted | 74 [6.2%] | 32 [2.6%] | -3.6 [-5.2 to -1.9] |
| Died | 5 [0.4%] | 1 [0.1%] | -0.3 [-0.7 to 0.1] |
| Length of stay (d) | 56 [42 to 98] | 42 [28 to 84] | 14 [0 to 14] |
| Total sachets provided | 84 [56 to 126] | 84 [56 to 112] | 14 [0 to 14] |
| Weight gain (kg) | 0.8 [0.5 to 1.1] | 0.6 [0.4 to 0.9] | 0.11 [0.10 to 0.20] |
| Rate of weight gain (g/kg/d) | 1.35 [0.84 to 2.25] | 1.41 [0.76 to 2.36] | -0.00 [-0.09 to 0.08] |
| Rate of weight gain in the first 2  weeks (g/kg/d) | 1.32 [0.00 to 2.90] | 1.93 [0.01 to 3.84] | -0.44 [-0.65 to -0.22] |
| MUAC gain (cm) | 0.6 [0.3 to 0.9] | 0.7 [0.4 to 0.9] | 0.0 [-0.1 to 0.0] |
| MUAC at discharge (cm) | 13.1 [12.8 to 13.9] | 12.9 [12.7 to 13.2] | 0.3 [0.2 to 0.3] |
| Length gain (cm) | 0.5 [0.2 to 1.0] | 0.5 [0.2 to 1.4] | -0.1 [-0.1 to -0.1] |
| Change in WHZ | 0.83 [0.47 to 1.23] | 0.64 [0.29 to 1.00] | 0.20 [0.15 to 0.25] |
| WHZ at discharge | -1.47 [-1.73 to -1.05] | -1.35 [-1.73 to -0.85] | -0.08 [-0.13 to -0.03] |
| Change in HAZ | -0.26 [-0.50 to -0.12] | -0.22 [-0.45 to -0.07] | -0.05 [-0.08 to -0.03] |
| HAZ at discharge | -2.12 [-3.33 to -0.98] | -2.33 [-3.29 to -1.25] | 0.09 [-0.05 to 0.22] |
| Change in WAZ | 0.41 [0.17 to 0.67] | 0.33 [0.07 to 0.56] | 0.09 [0.06 to 0.13] |
| WAZ at discharge | -2.12 [-2.73 to -1.44] | -2.09 [-2.75 to -1.50] | 0.02 [-0.06 to 0.09] |

Data are n (%) or median (IQR), as appropriate. Differences are presented as risk differences (percentage points) or median differences with 95% confidence intervals and are calculated as Gode minus Teltele.

MUAC = mid-upper arm circumference; WHZ = weight-for-height z-score; HAZ = height-for-age z-score; WAZ = weight-for-age z-score; MAM = moderate acute malnutrition; SAM = severe acute malnutrition

**S11b Table: Primary Outcomes for children treated for moderate acute malnutrition, stratified by study site in intention-to-treat analysis**

|  | **1-RUSF** | **1-RUTF** | **2-RUTF** | **1-RUSF vs. 1-RUTF** | **1-RUSF vs. 2-RUTF** | | **1-RUTF vs. 2-RUTF** |
| --- | --- | --- | --- | --- | --- | --- | --- |
|  |  |  |  | Difference | Difference | | Difference |
| **Gode Woreda** | **n=403** | **n=387** | **n=405** |  |  |  | |
| Recovered | 350 [86.9%] | 334 [86.3%] | 360 [88.9%] | -0.5 [-5.3 to 4.2] | 2.0 [-2.5 to 6.5] | 2.6 [-2.0 to 7.2] | |
| Not recovered | 53 [13.2%] | 53 [13.7%] | 45 [11.1%] | 0.5 [-4.2 to 5.3] | -2.0 [-6.5 to 2.5] | -2.6 [-7.2 to 2.0] | |
| Deteriorated to SAM | 9 [2.2%] | 6 [1.6%] | 1 [0.3%] | -0.7 [-2.6 to 1.2] | -2.0 [-3.5 to -0.5] | -1.3 [-2.6 to 0.0] | |
| Referred to Hospital | 3 [0.7%] | 8 [2.1%] | 9 [2.2%] | 1.3 [-0.3 to 3.0] | 1.5 [-0.2 to 3.1] | 0.2 [-1.9 to 2.2] | |
| Remained with MAM | 12 [3.0%] | 15 [3.9%] | 9 [2.2%] | 0.9 [-1.6 to 3.4] | -0.8 [-3.0 to 1.4] | -1.7 [-4.1 to 0.8] | |
| Defaulted | 27 [6.7%] | 23 [5.9%] | 24 [5.9%] | -0.8 [-4.2 to 2.6] | -0.8 [-4.1 to 2.6] | -0.0 [-3.3 to 3.3] | |
| Died | 2 [0.5%] | 1 [0.3%] | 2 [0.5%] | -0.2 [-1.1 to 0.6] | -0.0 [-1.0 to 1.0] | 0.2 [-0.6 to 1.1] | |
| Length of stay (d) | 56 [42 to 98] | 70 [42 to 98] | 56 [42 to 98] | 0 [0 to 0] | 0 [0 to 0] | 0 [0 to 0] | |
| Total sachets provided | 70 [56 to 98] | 70 [56 to 98] | 140 [84 to 196] | 0 [0 to 0] | -70 [-70 to -56] | -70 [-70 to -56] | |
| **Teltele Woreda** | **n=398** | **n=406** | **n=413** |  |  |  | |
| Recovered | 329 [82.7%] | 340 [83.7%] | 361 [87.4%] | 1.1 [-4.1 to 6.3] | 4.8 [-0.2 to 9.7] | 3.7 [-1.1 to 8.5] | |
| Not recovered | 69 [17.3%] | 66 [16.3%] | 52 [12.6%] | -1.1 [-6.3 to 4.1] | -4.8 [-9.7 to 0.2] | -3.7 [-8.5 to 1.1] | |
| Deteriorated to SAM | 7 [1.8%] | 7 [1.7%] | 9 [2.2%] | -0.0 [-1.8 to 1.8] | 0.4 [-1.5 to 2.3] | 0.5 [-1.4 to 2.4] | |
| Referred to Hospital | 3 [0.8%] | 4 [1.0%] | 7 [1.7%] | 0.2 [-1.1 to 1.5] | 0.9 [-0.6 to 2.5] | 0.7 [-0.9 to 2.3] | |
| Remained with MAM | 47 [11.8%] | 40 [9.9%] | 30 [7.3%] | -2.0 [-6.3 to 2.3] | -4.6 [-8.6 to -0.5] | -2.6 [-6.4 to 1.2] | |
| Defaulted | 12 [3.0%] | 14 [3.5%] | 6 [1.5%] | 0.4 [-2.0 to 2.9] | -1.6 [-3.6 to 0.5] | -2.0 [-4.1 to 0.1] | |
| Died | 0 [0.0%] | 1 [0.3%] | 0 [0.0%] | 0.3 [-0.2 to 0.7] | 0.0 [0.0 to 0.0] | -0.3 [-0.7 to 0.2] | |
| Length of stay (d) | 56 [28 to 84] | 56 [28 to 84] | 42 [28 to 70] | 0 [0 to 0] | 0 [0 to 0] | 0 [0 to 0] | |
| Total sachets provided | 56 [42 to 84] | 56 [42 to 84] | 112 [84 to 168] | 0 [0 to 0] | -42 [-56 to -42] | -49 [-56 to -42] | |

Data are n [%] or median [IQR], as appropriate. Effect estimates are presented as risk differences (percentage points) or median differences with 95% confidence intervals. Risk difference is calculated as experimental arm minus control (1-RUSF). Results are stratified by study site and should be interpreted as sub-analyses

MAM = moderate acute malnutrition; SAM = severe acute malnutrition; RUTF = ready-to-use therapeutic food; RUSF = ready-to-use supplementary food

**Supplemental Table S11c: Growth indicators for children treated for moderate acute malnutrition, stratified by study site in intention-to-treat analysis**

|  | **1-RUSF** | **1-RUTF** | **2-RUTF** | **1-RUSF vs. 1-RUTF** | **1-RUSF vs. 2-RUTF** | **1-RUTF vs. 2-RUTF** |
| --- | --- | --- | --- | --- | --- | --- |
|  |  |  |  | Difference | Difference | Difference |
| **Gode Woreda** | **n=403** | **n=386** | **n=405** |  |  |  |
| Weight gain (kg) | 0.80 [0.50 to 1.10] | 0.70 [0.40 to 1.03] | 0.80 [0.50 to 1.10] | 0.00 [0.00 to 0.10] | 0.00 [-0.00 to 0.10] | 0.00 [-0.10 to 0.00] |
| Rate of weight gain (g/kg/d) | 1.37 [0.88 to 2.20] | 1.26 [0.80 to 2.13] | 1.39 [0.85 to 2.38] | 0.09 [-0.04 to 0.23] | -0.01 [-0.16 to 0.13] | -0.11 [-0.26 to 0.03] |
| Rate of weight gain in the first 2  weeks (g/kg/d) | 1.21 [0.00 to 3.03] | 1.35 [0.00 to 2.79] | 1.48 [0.00 to 3.02] | 0.00 [-0.29 to 0.28] | -0.08 [-0.44 to 0.15] | -0.08 [-0.41 to 0.15] |
| MUAC gain (cm) | 0.7 [0.3 to 0.9] | 0.6 [0.3 to 0.9] | 0.6 [0.4 to 0.9] | 0.0 [-0.0 to 0.1] | 0.0 [-0.1 to 0.1] | 0.0 [-0.1 to 0.1] |
| MUAC at discharge (cm) | 13.1 [12.8 to 13.8] | 13.2 [12.8 to 13.9] | 13.1 [12.8 to 13.9] | -0.1 [-0.2 to 0.0] | 0.0 [-0.1 to 0.0] | 0.0 [-0.1 to 0.1] |
| Length gain (cm) | 0.5 [0.2 to 0.9] | 0.5 [0.1 to 1.1] | 0.5 [0.2 to 1.0] | 0.0 [-0.1 to 0.0] | 0.0 [-0.0 to 0.1] | 0.0 [0.0 to 0.1] |
| Change in WHZ | 0.87 [0.50 to 1.25] | 0.78 [0.43 to 1.14] | 0.84 [0.48 to 1.25] | 0.09 [0.01 to 0.17] | 0.03 [-0.05 to 0.11] | -0.06 [-0.14 to 0.02] |
| WHZ at discharge | -1.43 [-1.70 to -0.96] | -1.52 [-1.76 to -1.17] | -1.47 [-1.71 to -0.99] | 0.11 [-0.04 to 0.18] | 0.03 [-0.04 to 0.11] | -0.07 [-0.14 to -0.01] |
| Change in HAZ | -0.26 [-0.50 to -0.14] | -0.25 [-0.52 to -0.11] | -0.25 [-0.48 to -0.12] | -0.03 [-0.07 to 0.01] | -0.02 [-0.05 to 0.02] | 0.01 [-0.03 to 0.05] |
| HAZ at discharge | -2.41 [-3.35 to -1.33] | -2.13 [-3.26 to -0.85] | -2.18 [-3.38 to -0.86] | -0.31 [-0.55 to -0.06] | -0.23 [-0.48 to 0.02] | 0.07 [-0.19 to 0.33] |
| Change in WAZ | 0.43 [0.17 to 0.67] | 0.39 [0.15 to 0.66] | 0.42 [0.18 to 0.71] | 0.04 [-0.02 to 0.09] | 0.00 [-0.06 to 0.05] | -0.04 [-0.09 to 0.02] |
| WAZ at discharge | -2.22 [-2.76 to -1.53] | -2.10 [-2.72 to -1.38] | -2.04 [-2.72 to -1.38] | -0.10 [-0.24 to 0.04] | -0.14 [-0.27 to 0.00] | -0.03 [-0.18 to 0.11] |
| **Teltele Woreda** | **n=398** | **n=406** | **n=413** |  |  |  |
| Weight gain (kg) | 0.66 [0.40 to 0.90] | 0.60 [0.35 to 0.89] | 0.61 [0.40 to 0.90] | 0.04 [-0.00 to 0.10] | 0.00 [-0.04 to 0.09] | -0.01 [-0.10 to 0.00] |
| Rate of weight gain (g/kg/d) | 1.47 [0.78 to 2.38] | 1.28 [0.68 to 2.21] | 1.41 [0.83 to 2.46] | 0.17 [0.01 to 0.33] | -0.03 [-0.20 to 0.13] | -0.20 [-0.35 to -0.04] |
| Rate of weight gain in the first 2  weeks (g/kg/d) | 2.02 [0.19 to 3.97] | 1.65 [0.00 to 3.42] | 2.02 [0.46 to 4.02] | 0.32 [0.00 to 0.72] | -0.09 [-0.54 to 0.24] | -0.46 [-0.83 to -0.05] |
| MUAC gain (cm) | 0.6 [0.3 to 0.9] | 0.6 [0.4 to 0.9] | 0.7 [0.4 to 1.0] | 0.0 [-0.1 to 0.0] | -0.1 [-0.2 to 0.0] | -0.1 [-0.1 to 0.0] |
| MUAC at discharge (cm) | 12.9 [12.6 to 13.2] | 12.9 [12.7 to 13.2] | 13.0 [12.7 to 13.3] | 0.0 [-0.1 to 0.0] | -0.1 [-0.1 to 0.0] | 0.0 [-0.1 to 0.0] |
| Length gain (cm) | 0.6 [0.2 to 1.4] | 0.5 [0.2 to 1.5] | 0.5 [0.2 to 1.5] | 0.0 [0.0 to 0.1] | 0.0 [0.0 to 0.1] | 0.0 [-0.1 to 0.1] |
| Change in WHZ | 0.68 [0.31 to 1.05] | 0.59 [0.27 to 0.95] | 0.66 [0.30 to 0.99] | 0.08 [0.00 to 0.16] | 0.03 [-0.05 to 0.11] | -0.05 [-0.13 to 0.03] |
| WHZ at discharge | -1.37 [-1.70 to -0.83] | -1.40 [-1.83 to -0.95] | -1.30 [-1.70 to -0.79] | 0.08 [-0.01 to 0.16] | -0.04 [-0.13 to 0.05] | -0.12 [-0.20 to -0.03] |
| Change in HAZ | -0.23 [-0.46 to -0.08] | -0.20 [-0.44 to -0.07] | -0.24 [-0.45 to -0.07] | -0.03 [-0.07 to 0.01] | -0.02 [-0.06 to 0.03] | 0.01 [-0.03 to 0.05] |
| HAZ at discharge | -2.45 [-2.26 to -1.35] | -2.16 [-3.13 to -1.17] | -2.33 [-3.31 to -1.25] | -0.26 [-0.47 to -0.05] | -0.12 [-0.34 to 0.09] | 0.13 [-0.08 to 0.34] |
| Change in WAZ | 0.35 [0.06 to 0.58] | 0.20 [0.06 to 0.53] | 0.33 [0.09 to 0.57] | 0.04 [-0.01 to 0.10] | 0.00 [-0.05 to 0.06] | -0.04 [-0.09 to 0.01] |
| WAZ at discharge | -2.11 [-2.77 to -1.61] | -2.10 [-2.71 to -1.49] | -2.05 [-2.76 to -1.43] | -0.06 [-0.18 to 0.07] | -0.09 [-0.21 to 0.04] | -0.02 [-0.15 to 0.10] |

Data are median [IQR]. Differences are presented as median difference with 95% confidence intervals. Differences are defined as experimental arm minus control (1-RUSF). Results are stratified by study site and should be interpreted as sub-analyses. MUAC = mid-upper arm circumference; WHZ = weight-for-height z-score; HAZ = height-for-age z-score; WAZ = weight-for-age z-score; RUTF = ready-to-use therapeutic food; RUSF = ready-to-use supplementary food.
