# Supplementary material for: Comparative effectiveness of lipid-nutrient spread supplementation regimens for moderate acute malnutrition in Ethiopia: The MODAM-MAM individually randomized controlled trial
Source: PLoS Med. 2026 Aug 11;23(8):e1005199. doi: 10.1371/journal.pmed.1005199 (PMC13475968; doi:10.1371/journal.pmed.1005199)
Supplement: Supplementary file 13 [file pmed.1005199.s012.docx]

**S12 Table. Tests for interaction between treatment arm and sub-groups on recovery**

| **Subgroup Variable** | p-value |
| --- | --- |
| Age at admission (6-23 vs 24-59 months) | 0.50 |
| MUAC at admission (11.5-11.9 vs ≥12.0 cm) | 0.45 |
| WAZ at admission (< -3 vs ≥ −3) | 0.44 |
| Woreda (Teltele vs Gode) | 0.81 |

Interaction tests were conducted using logistic regression models with recovery as the dependent variable and including treatment arm, subgroup indicator, and treatment-by-subgroup interaction terms. P-values for interaction were obtained using joint Wald tests of the interaction terms and were used to assess evidence of effect modification across subgroups. Subgroup-specific treatment effect estimates and outcome distributions are presented in Tables S8–S11.

MUAC = mid-upper arm circumference; WAZ = weight-for-age z-score
