# Supplementary material for: Comparative effectiveness of lipid-nutrient spread supplementation regimens for moderate acute malnutrition in Ethiopia: The MODAM-MAM individually randomized controlled trial
Source: PLoS Med. 2026 Aug 11;23(8):e1005199. doi: 10.1371/journal.pmed.1005199 (PMC13475968; doi:10.1371/journal.pmed.1005199)
Supplement: Supplementary file 14 [file pmed.1005199.s013.docx]

**S13 Table: Estimated contribution of study rations (RUSF and RUTF) to recommended daily energy requirements among children with moderate acute malnutrition**

| **Weight range (kg)** | **Midpoint (kg)** | **Daily kcal need (100-130kcal/kg from midpoint)** | **40-60% of daily kcal** | **kcal from 1 RUSF sachet** | **kcal from 1 RUTF sachet** | **kcal from 2 RUTF sachets** |
| --- | --- | --- | --- | --- | --- | --- |
| 3.5-3.9 | 3.7 | 370-481 | 148-289 | 535 | 500 | 1000 |
| 4.0-5.4 | 4.7 | 470-611 | 188-367 | 535 | 500 | 1000 |
| 5.5-6.9 | 6.2 | 620-806 | 248-484 | 535 | 500 | 1000 |
| 7.0-8.4 | 7.7 | 770-1001 | 308-601 | 535 | 500 | 1000 |
| 8.5-9.4 | 9 | 900-1170 | 360-702 | 535 | 500 | 1000 |
| 9.5-10.4 | 10 | 1000-1300 | 400-780 | 535 | 500 | 1000 |
| 10.5-11.9 | 11.2 | 1120-1456 | 448-874 | 535 | 500 | 1000 |
| ≥ 12 | 12.5 | 1250-1625 | 500-975 | 535 | 500 | 1000 |

Values are estimated using midpoints of each weight range and assumed energy requirements of 100–130 kcal/kg/day, consistent with international guidance for children with moderate acute malnutrition. The target contribution of supplementary foods is assumed to be approximately 40–60% of total daily energy requirements. Energy content per sachet was assumed to be 535 kcal for RUSF and 500 kcal for RUTF. These values are illustrative and intended to contextualize dosing strategies rather than represent precise individual requirements.

RUTF=ready-to-use therapeutic food; RUSF=ready-to-use supplementary food; kcal=kilocalories
