# Supplementary material for: Comparative effectiveness of lipid-nutrient spread supplementation regimens for moderate acute malnutrition in Ethiopia: The MODAM-MAM individually randomized controlled trial
Source: PLoS Med. 2026 Aug 11;23(8):e1005199. doi: 10.1371/journal.pmed.1005199 (PMC13475968; doi:10.1371/journal.pmed.1005199)
Supplement: Supplementary file 15 [file pmed.1005199.s014.docx]

**S1 Fig: Time to recovery among all children treated for moderate acute malnutrition by treatment group**

Kaplan–Meier curves show time to recovery by treatment group. Differences between groups were assessed using log-rank tests. Numbers at risk are shown below the figure.

Log rank: across all groups, p=0.015; between 1 sachet RUSF/day vs 1 sachet RUTF/day, p=0.620; between 1 sachet RUSF/day vs 2 sachets RUTF/day, p=0.006; between 1 sachet RUTF/day vs 2 sachets RUTF/day, p=0.028.

RUTF=ready-to-use therapeutic food; RUSF=ready-to-use supplementary food
