# Supplementary material for: Comparative effectiveness of lipid-nutrient spread supplementation regimens for moderate acute malnutrition in Ethiopia: The MODAM-MAM individually randomized controlled trial
Source: PLoS Med. 2026 Aug 11;23(8):e1005199. doi: 10.1371/journal.pmed.1005199 (PMC13475968; doi:10.1371/journal.pmed.1005199)
Supplement: Supplementary file 16 [file pmed.1005199.s015.docx]

**S2 Fig: Growth velocity by baseline MUAC and treatment group**

|  |  |
| --- | --- |

Panel A shows weight gain velocity (g/kg/day) and Panel B shows MUAC gain velocity (mm/week), each plotted against MUAC at the start of the visit interval. Lines represent smoothed estimates of growth velocity across the observed range of MUAC values using locally weighted regression (LOESS), with shaded bands indicating 95% confidence intervals.

MUAC = mid–upper arm circumference; RUTF = ready-to-use therapeutic food; RUSF = ready-to-use supplementary food
