# Supplementary material for: Comparative effectiveness of lipid-nutrient spread supplementation regimens for moderate acute malnutrition in Ethiopia: The MODAM-MAM individually randomized controlled trial
Source: PLoS Med. 2026 Aug 11;23(8):e1005199. doi: 10.1371/journal.pmed.1005199 (PMC13475968; doi:10.1371/journal.pmed.1005199)
Supplement: Supplementary file 18 [file pmed.1005199.s017.docx]

Inclusivity in global research

PLOS’ policy on inclusivity in global research aims to improve transparency in the reporting of research performed outside of researchers’ own country or community and ensures that PLOS publications reporting global research adhere to high standards for research ethics and authorship. Authors of relevant research articles may be asked to complete the questionnaire below, which outlines ethical, cultural, and scientific considerations specific to inclusivity in global research. This questionnaire may be requested when researchers have travelled to a different country to conduct research, if research uses samples collected in another country, research with Indigenous populations or their lands, or if research is on cultural artefacts. Researchers travelling to another country solely to use laboratory equipment will not normally be required to complete the questionnaire. However, the questionnaire can be requested at the journal’s discretion for any submission – if you have been requested to complete this questionnaire by the PLOS journal you submitted to, please do so.

Please complete the questionnaire below and include this as a Supporting Information file with your manuscript. Note that if your paper is accepted for publication, this checklist will be published with your article in the supporting information files. Please ensure that you reference the checklist in the main body of your manuscript. We suggest adding a subsection ‘Inclusivity in global research’ to your Methods section and adding the following sentence: “Additional information regarding the ethical, cultural, and scientific considerations specific to inclusivity in global research is included in the Supporting Information (SX Checklist)”

The questions have been designed to be applicable to a wide range of study types, and there are subsections for both human subjects research and non-human subjects research. If any of the questions are not relevant to your research please mark them as “N/A” as appropriate.

**Ethical considerations, permits and authorship**

*This section is applicable to all research types.*

Provide details as to who granted permissions and/or consent for the study to take place in the Methods section of your manuscript. This should include the names of **all** ethics boards, governmental organizations, community leaders or other bodies that provided approval for the study. If individuals provided approval refer to these people by their role or title but do not list their name(s).

This is reported in the manuscript as requested.

If there were any deviations from the study protocol after approval was obtained please provide details of these changes in the Methods section of your manuscript.

This is reported in the manuscript as requested.

Did this study involve local collaborators that are residents of the country where the research was conducted or members of the community studied? If you do not have any authors from said communities, please provide an explanation for this below.

Local collaborators included Ethiopian investigators from the Ethiopia Public Health Institute, Federal Ministry of Health, Action Against Hunger Ethiopia, and UNICEF Ethiopia. Ethiopian collaborators contributed to study design, implementation, data collection, interpretation of findings, and manuscript preparation. Authors from Ethiopian institutions are included in the author byline in accordance with PLOS and ICMJE authorship criteria. Additional Ethiopian research and implementation staff contributed to study implementation and are acknowledged in the manuscript.

Everyone listed as an author should meet PLOS’ criteria for authorship and all individuals who meet these criteria should be included in the author byline, rather than the acknowledgements. For further information please see the journal’s Authorship Policy.

**Human subjects research (e.g. health research, medical research, cross-cultural psychology)**

Did you obtain written informed consent from a representative of the local community or region before the research took place? How did you establish who speaks for the community? Details of written informed consent obtained from study participants should be reported separately in the Methods section of your manuscript.

Prior to study implementation, approvals were obtained from the Ethiopia Public Health Institute Institutional Review Board (EPHI-IRB) and relevant governmental and health authorities, including the Federal Ministry of Health, Oromia, Amhara, and Somali Regional Health Bureaus, woreda health offices, and participating health facilities. Written letters of support and approval were obtained at federal, regional, zonal, and woreda, levels prior to study initiation. These governmental and health authorities are responsible for oversight of health services and health research within their respective jurisdictions and served as the recognized representatives for approving and supporting study implementation. Individual informed consent from caregivers of participating children was obtained separately, as described in the Methods section.

How did members of the local community provide input on the aims of the research investigation, its methodology, and its anticipated outcome(s)?

The study was developed in collaboration with Ethiopian partners, including the Ethiopia Public Health Institute (EPHI), the Federal Ministry of Health (FMOH), Ethiopian staff from Action Against Hunger, and UNICEF Ethiopia. Study objectives were informed by national and global policy questions regarding the management of moderate acute malnutrition and were designed to address operational challenges identified by implementing partners and government stakeholders.

During the study design phase, Action Against Hunger hosted a consultative project inception workshop in Addis Ababa in August 2022 in collaboration with UNICEF and the Federal Ministry of Health. Participants included representatives from government institutions, UN agencies, academic institutions, donors, and implementing partners. The workshop provided an opportunity to review formative research findings, discuss and refine the proposed study design and protocols, identify priority research questions, proactively address implementation challenges, and inform plans for dissemination and uptake of study findings.

Prior to implementation, the study protocol was also presented to the relevent Regional Health Bureaus and discussed with stakeholders at multiple administrative levels. Sub-national project launching workshops were conducted to engage federal, regional, zonal, woreda, and health facility stakeholders, build local ownership, and support implementation planning.

When engaging with the local community, how did you ensure that the informed consent documents and other materials could be understood by local stakeholders?

Study materials, including informed consent documents, were translated into relevant local languages and reviewed by Ethiopian research and program staff prior to implementation. Consent information was delivered verbally by trained local study staff in the participant's preferred language. For caregivers who were unable to read or write, consent procedures included verbal explanation and documentation through fingerprint signatures in accordance with approved ethical procedures.

Will the findings of the research be made available in an understandable format to stakeholders in the community where the study was conducted (e.g. via a presentation, summary report, copies of publications, etc.)? Please provide details of how this will be achieved.

Yes. Study findings have been and will continue to be shared with stakeholders through presentations and dissemination meetings involving the Ethiopia Public Health Institute, Federal Ministry of Health, UNICEF Ethiopia, Nutrition Cluster, national Technical Working Groups, regional and woreda health authorities, and Action Against Hunger Ethiopia.

A national dissemination workshop will be conducted in Addis Ababa with representatives from government, UN agencies, academic institutions, donors, and implementing partners. In addition, sub-national dissemination workshops will be held in the study regions with regional and local authorities, health workers, and other stakeholders. Dissemination materials and presentations will be adapted and translated as needed to ensure findings are accessible and understandable to local audiences.

Findings will also be disseminated through peer-reviewed publications, conference presentations, technical reports, and policy discussions intended to inform national and global guidance on the management of acute malnutrition.

**Non-human subjects research using specimens/ animals collected as part of the study, or those housed in archival collections. Examples include archaeology, paleontology, botany and zoology.**

Did the permission you obtained from a local authority to perform the study include an agreement on access to outputs and benefit sharing? This may include procedures to enable fair distribution of the benefits and resources arising from the research performed. Please include any details of Prior Informed Consent and Benefit Sharing Agreements obtained. These may be required by field-specific regulations, for example the Convention on Biological Diversity (CBD) and the associated Nagoya Protocol.

Not applicable.

If the material used in your study was imported, please A) provide the year it was imported and B) indicate whether permits were obtained to import/export the materials used, C) provide details of any permits obtained. If this information is not available, please indicate this.

Not applicable.

If you used archival specimens, please state how the material used in your study was acquired by the institute it is held in and provide details of any permits obtained for the original excavations/ sample collection. If this information is not available, please indicate this.

Not applicable.

How was the potential cultural significance of the materials collected in your study to local communities considered in your research design? Were Indigenous peoples and/or local researchers and institutions involved with archaeological excavations / collection of specimens? If so, please provide a description of their involvement.

Not applicable.

If your manuscript includes photographs of human remains please indicate whether authors obtained permission from descendants or affiliated cultural communities to do so.

Not applicable.
